# Methylomic markers of persistent childhood asthma: a longitudinal study of asthma-discordant monozygotic twins

**DOI:** 10.1186/s13148-015-0163-4

**Published:** 2015-12-18

**Authors:** Therese M. Murphy, Chloe C. Y. Wong, Louise Arseneault, Joe Burrage, Ruby Macdonald, Eilis Hannon, Helen L. Fisher, Antony Ambler, Terrie E. Moffitt, Avshalom Caspi, Jonathan Mill

**Affiliations:** University of Exeter Medical School, University of Exeter, Exeter, UK; MRC Social, Genetic & Developmental Psychiatry Centre, Institute of Psychiatry, Psychology & Neuroscience, King’s College London, London, UK; Department of Psychology and Neuroscience, Duke University, Durham, NC USA; Department of Psychiatry and Behavioral Sciences, Duke University Medical School, Durham, NC USA

**Keywords:** Asthma, DNA methylation, Epigenetics, Monozygotic twins

## Abstract

**Background:**

Asthma is the most common chronic inflammatory disorder in children. The aetiology of asthma pathology is complex and highly heterogeneous, involving the interplay between genetic and environmental risk factors that is hypothesized to involve epigenetic processes. Our aim was to explore whether methylomic variation in early childhood is associated with discordance for asthma symptoms within monozygotic (MZ) twin pairs recruited from the Environmental Risk (E-Risk) longitudinal twin study. We also aimed to identify differences in DNA methylation that are associated with asthma that develops in childhood and persists into early adulthood as these may represent useful prognostic biomarkers.

**Results:**

We examined genome-wide patterns of DNA methylation in buccal cell samples collected from 37 MZ twin pairs discordant for asthma at age 10. DNA methylation at individual CpG sites demonstrated significant variability within discordant MZ twin pairs with the top-ranked nominally significant differentially methylated position (DMP) located in the *HGSNAT* gene. We stratified our analysis by assessing DNA methylation differences in a sub-group of MZ twin pairs who remained persistently discordant for asthma at age 18. The top-ranked nominally significant DMP associated with persisting asthma is located in the vicinity of the *HLX* gene, which has been previously implicated in childhood asthma.

**Conclusions:**

We identified DNA methylation differences associated with childhood asthma in peripheral DNA samples from discordant MZ twin pairs. Our data suggest that differences in DNA methylation associated with childhood asthma which persists into early adulthood are distinct from those associated with asthma which remits.

**Electronic supplementary material:**

The online version of this article (doi:10.1186/s13148-015-0163-4) contains supplementary material, which is available to authorized users.

## Background

Asthma is a common, complex and highly heterogeneous disorder characterized by recurring episodes of breathlessness and wheezing [1]. Asthma affects approximately 235 million people worldwide and is the most prevalent chronic inflammatory disorder in children [2]. The natural history of childhood-onset asthma is complex; it is commonly characterized by periods of remission and relapse [3, 4]. The heterogeneous features of asthma may indicate sub-types of the disease with distinct molecular pathology [5, 6], and recent analyses suggest that remitting and persistent childhood-onset asthma is aetiologically distinct [7]. Currently, the diagnosis of asthma relies heavily on clinical features [1]. Understanding the molecular mechanisms involved in the onset and stability of asthma will facilitate the identification of children at increased risk of developing severe and persistent asthma in the future.

Asthma is known to involve complex interplay between genetic and environmental risk factors. Twin studies have demonstrated that the aetiology of asthma has a significant genetic contribution, with heritability estimates ranging from 35 to 80 % [8–10]. Genome-wide association studies (GWAS) have identified a small number of common risk variants robustly associated with asthma, including a gene-rich locus on chromosome 17q21 specific to childhood-onset disease [11]. Identified variants, however, account for only a small proportion of the estimated heritability [1, 11, 12]. Epidemiological research also highlights associations between specific environmental exposures (including tobacco smoke [4, 13] and microbial and viral agents [14, 15]) and the onset of asthma, with exposures at certain critical periods during early childhood being particularly important [1]. Recent studies have started to examine the role of epigenetic processes—acting to developmentally regulate gene expression via modifications to DNA, histone proteins and chromatin—in complex disease phenotypes [16] including asthma. A number of studies have identified locus-specific variation in DNA methylation associated with asthma pathogenesis [17–20]. One study linked differential DNA methylation in the vicinity of two candidate genes (*FOXP3* and *IFNγ*) with asthma in a study of *N* = 21 asthma-discordant adult monozygotic (MZ) twin pairs [18]. A recent longitudinal study observed asthma-related changes in DNA methylation at specific genetic loci including *IL4* and *IL4R* [17]. However, such published research examining epigenetic variation in asthma has primarily focused on candidate immune-related genes; few studies have taken a genome-wide approach [21]. Of note, a recent epigenome-wide methylation study (EWAS) identified significant DNA methylation changes at several loci associated with imunnunoglobulin E (IgE) levels, which are known to correlate positively with allergic diseases such as asthma [22]. However, most patient samples have been cross-sectional and thus it is unknown whether prior findings of methylomic differences are differentially associated with persistent or remitting asthma in early childhood.

The aim of the current study was to explore whether methylomic variation in early childhood is associated with discordance for asthma in 37 pairs of MZ twins. We were interested in identifying differences in DNA methylation that are associated with asthma that develops in childhood and persists into early adulthood as these may represent useful prognostic biomarkers. The use of disease-discordant MZ twins represents a powerful strategy in epigenetic epidemiology because identical twins are matched for genotype, age, sex, maternal environment, population cohort effects and exposure to many shared environmental factors during childhood [23]. We profiled DNA obtained from buccal swabs, which have previously been used as a surrogate for airway epithelial cells in DNA methylation studies [19].

## Results

### Brief overview of experimental approach

An overview of the methodological approach used in this study is given in Additional file 1: Figure S1. Briefly, we assessed genome-wide patterns of DNA methylation in asthma-discordant MZ twins and concordant unaffected MZ twins at age 10 using the Illumina 450K HumanMethylation microarray (450K array). Pre-processing, normalization and stringent quality control were performed as previously described [24] (see Methods for specific details). Our analyses focused on identifying differentially methylated positions (DMPs) associated with asthma in (i) all asthma-discordant MZ twins at age 10 (Additional file 1: Figure S1A) and (ii) a sub-group with persistent asthma who were discordant for asthma at age 10 and also at age 18 (Additional file 1: Figure S1B). Using DNA previously collected at age 5, we subsequently assessed longitudinal changes in DNA methylation (between ages 5 and 10) in persistent-asthma-discordant MZ twins (Additional file 1: Figure S1C). Finally, we examined epigenetic variation at top-ranked persistent-asthma-associated DMPs in (i) an asthma-remission group, comprising of MZ twin pairs discordant for asthma at age 10 but concordant for no asthma phenotype at 18, and (ii) concordant unaffected MZ twin pairs where neither twin had asthma at both ages 10 and 18 (Additional file 1: Figure S1D).

### Differentially methylated positions at age 10 associated with childhood asthma

**Fig. 1:**
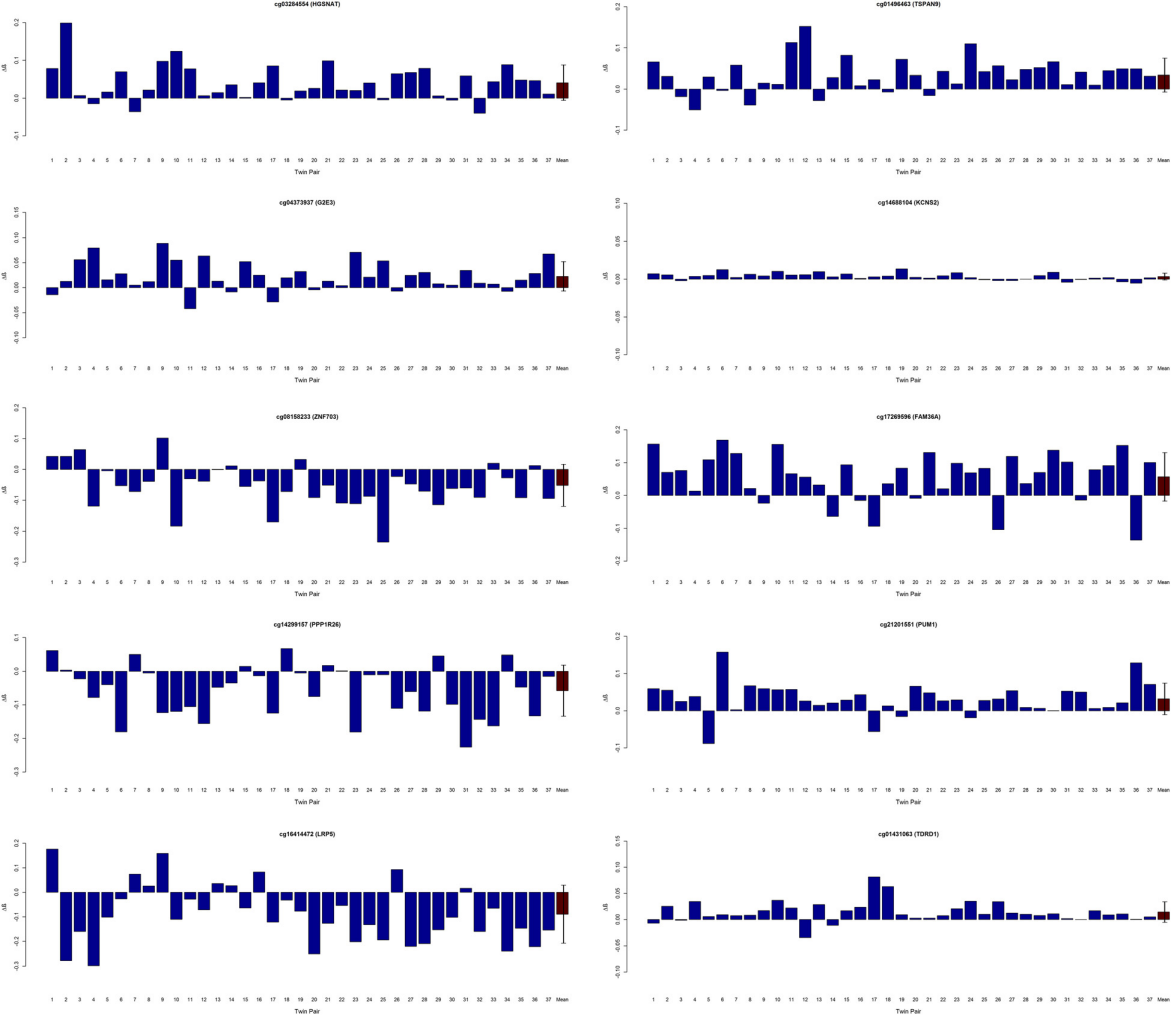
Graphs showing the difference in DNA methylation (∆*β*) at age 10 between each pair of monozygotic (MZ) twins discordant for asthma (affected twin − unaffected co-twin) for each of the ten top-ranked differentially methylated positions (DMPs). Mean within-twin pair ∆*β* across all 37 MZ twin pairs is highlighted in *red. Error bars* represent the ±standard deviation. Consistent within-twin pair differences in DNA methylation at age 10 are observed across asthma-discordant MZ twin pairs at the top ten ranked differentially methylated positions

Our primary focus was on within-pair DNA methylation differences detected at age 10, as an accurate diagnosis of asthma before age 5 is difficult [25, 26]. As expected, within-twin patterns of DNA methylation were highly correlated across all 37 MZ pairs (mean within-twin *r* for DNA methylation across all 450K array probes = 0.97) and no difference in overall mean DNA methylation (calculated by averaging across all probes on the 450K array) was observed between affected and unaffected twins (*P* = 0.81), indicating that childhood asthma is not associated with any global changes in DNA methylation. In contrast, DNA methylation at individual CpG sites demonstrated significant variability within discordant MZ twin pairs. Table 1 lists the top-ranked nominally significant DMPs associated with asthma at age 10 (listing all with a nominal *P* < 1 × 10^−4^). Although no DMP remained significant after Bonferroni multiple test correction, permutation analyses of the top-ranked loci revealed significant empirical *P* values. Consistent within-twin differences in DNA methylation were observed across the majority of discordant MZ twin pairs (*n* = 37) for these top-ranked DMPs (Fig. 1). The top-ranked DMP, cg03284554, located in the promoter region of the heparan-α-glucosaminide *N*-acetyltransferase (*HGSNAT*) gene on chromosome 8q21, was consistently hypermethylated in asthma-affected twins compared to their unaffected co-twin (mean Δ*β* = 0.041, *P* = 6.87E-06, permuted empirical *P* < 0.0001). Of note, *HGSNAT* gene expression is down-regulated in paediatric allergic asthma cases compared to controls [27]. Gene ontology (GO) term enrichment analysis of the age-10 DMPs (see Additional file 2: Table S1 for a list of included loci) identified 199 nominally significantly enriched terms (see Additional file 2: Table S2), including categories related to anion/ion transportation, regulation of ion transport (GO:0043269 (*P* = 0.0008), GO:0006811 (*P* = 0.003)) and regulation of anion transport (GO:0044070 (*P* = 0.0026)). Interestingly, changes in ion transport have previously been implicated in asthma pathogenesis [28].

**Table 1:** The top-ranked DMPs in asthma-discordant MZ twins

Probe ID	Co-twin mean	Asthma mean	Mean ∆*β*	*P* value	Empirical *P* value	Hg19	Relation to CpG island	Gene region feature category (UCSC)	Illumina gene annotation	Probe type	Gene annotation from GREAT (distance from TSS)
cg03284554	0.178	0.218	0.041	6.87E-06	<0.0001	Chr8:43113670	N_Shore	TSS1500	HGSNAT	II	HGSNAT (−1078)
cg01496463	0.761	0.795	0.034	1.80E-05	0.0002	Chr12:3262908		3′UTR	TSPAN9	II	PRMT8 (−207777), TSPAN9 (+206127)
cg04373937	0.836	0.859	0.023	4.26E-05	<0.0001	Chr14:29622576		Intergenic		II	G2E3 (−475503), PRKD1 (−155927)
cg14688104	0.035	0.038	0.003	4.29E-05	0.0001	Chr8:99508617	Island	1stExon; 5′UTR	KCNS2	I	KCNS2 (+192)
cg08158233	0.244	0.192	−0.052	5.36E-05	0.0001	Chr8:37470214		Intergenic		II	ZNF703 (−202244), KCNU1 (+709215)
cg17269596	0.581	0.637	0.056	5.49E-05	0.0004	Chr1:243061735	N_Shelf	Intergenic		II	FAM36A (−3526)
cg14299157	0.813	0.756	−0.058	5.59E-05	<0.0001	Chr9:137272770	N_Shelf	Intergenic		II	PPP1R26 (−238698), OLFM1 (+165861)
cg21201551	0.747	0.779	0.032	5.90E-05	0.0001	Chr1:31404820	S_Shelf	Intergenic		II	PUM1 (−93670), NKAIN1 (+80500)
cg16414472	0.503	0.414	−0.089	6.05E-05	0.0002	Chr11:67866681		Body	LRP5	I	PPP6R3 (−118080), LRP5 (+29998)
cg01431063	0.057	0.072	0.014	8.92E-05	<0.0001	Chr10:115850893	Island	Intergenic		II	TDRD1 (−78125), ADRB1 (+57098)
cg01048931	0.071	0.084	0.013	9.42E-05	<0.0001	Chr7:55639931	Island	Body	VOPP1	I	VOPP1 (+268)

Ranked by *P* value. Empirical *P* value = (number of permutations which are at least as significant as the true result (*P* < 0.0001) divided by the number of permutations performed (*n* =10000))

*DMPs* differentially methylated positions, *MZ* monozygotic, *GREAT* Genomic Regions Enrichment of Annotations Tool, *TSS* transcription start site, *UCSC* University of California Santa Cruz genome browser

### Methylomic profiling of MZ twins in a persistent-asthma sub-group

**Fig. 2:**
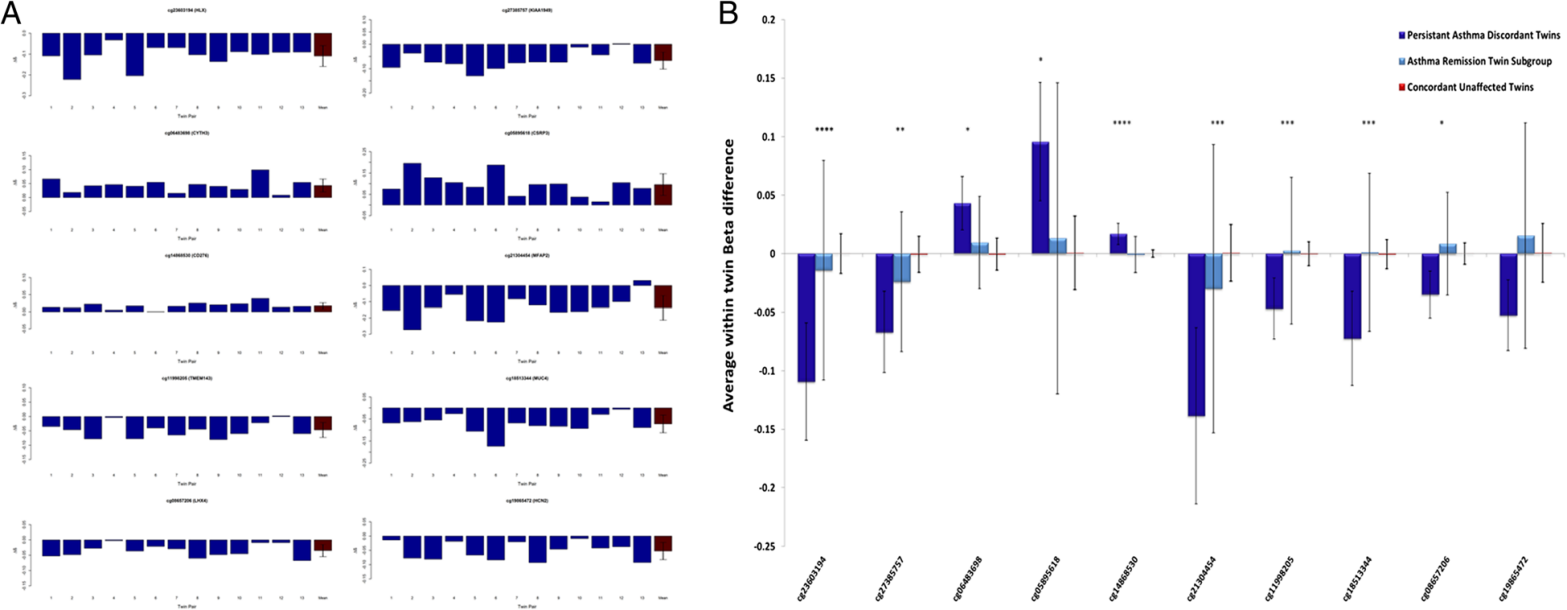
**a** Graphs showing the difference in DNA methylation (∆*β*) at age 10 between each pair of monozygotic (MZ) twins persistently discordant (at ages 10 and 18) for asthma (affected twin − unaffected co-twin) for each of the ten top-ranked differential methylated positions (DMPs). Mean within-twin pair ∆*β* across all 13 MZ twin pairs is highlighted in *red. Error bars* represent the ±standard deviation. Consistent within-twin pair differences in DNA methylation are observed across persistently discordant MZ twin pairs at the ten top-ranked DMPs. **b** Graph showing average within-twin beta difference of (i) persistent-asthma-discordant MZ twins at age 10, (ii) asthma-remission sub-group (twins discordant for asthma at age 10 but the affected twin had remitted at 18, *n* = 20 twin pairs) and the age-matched concordant unaffected MZ twins (19 twin pairs). Average within-twin differences in DNA methylation are significantly larger at nine of the ten top-ranked DMPs in asthma-discordant twins compared to both the asthma-remission sub-group and twins concordant for no asthma. Between group comparisons of average within-twin beta differences were examined using a one-way ANOVA. ****P < 0.0001, ***P < 0.001, **P < 0.01, *P < 0.05

**Fig. 3:**
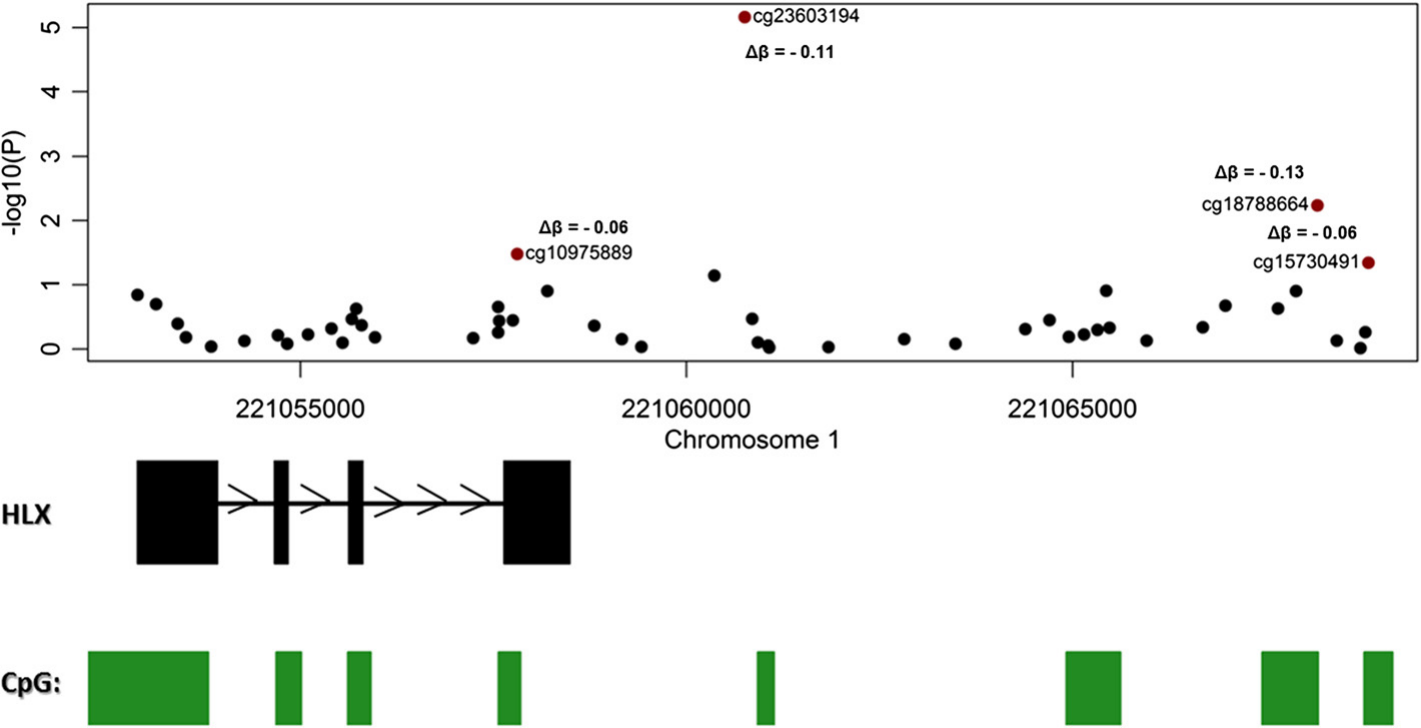
A number of probes annotated to the *HLX* gene were significantly differentially methylated in persistent-asthma twins compared to their unaffected co-twin. Asthma-associated differentially methylated positions are highlighted in *red. Green bars* denote the location of annotated CpG islands. Of note, asthma-associated DMPs overlap known CpG islands downstream of the *HLX* gene. ∆*β* = mean within-twin beta difference

Childhood asthma regularly remits in adolescence and early adulthood [3, 4], with remitting and persistent childhood asthma potentially having differing aetiology [7]. We therefore stratified our analysis by assessing age-10 DNA methylation differences in a sub-group of MZ twin pairs (*n* = 13 pairs) who remained persistently discordant for asthma at age 18. Table 2 and Fig. 2 show the top-ranked age-10 DMPs associated with asthma that persisted to age 18, which may represent useful childhood biomarkers of prognosis. Again, no DMP remained significant after Bonferroni correction, but empirical *P* values generated from permutation analyses suggest that these represent significant differences. The top-ranked DMP associated with persisting asthma (cg23603194, located downstream of the *HLX* gene) was consistently hypomethylated in asthma-affected twins compared with their unaffected co-twin (mean Δ*β* = −0.11, *P* = 6.87E-06, permuted empirical *P* = 0.0009). Interestingly, a number of additional probes (cg18788664, cg10975889, cg15730491) annotated to the *HLX* gene region were also differentially methylated in twins discordant for persistent asthma compared to their unaffected co-twin (see Fig. 3). Of note, genetic variation in this gene has been previously associated with the development of childhood asthma [29, 30] and cytokine secretion at birth [31]. We next tested the specificity of our persistent-asthma-associated DMPs, by comparing average within-twin DNA methylation differences at these loci in (i) an asthma-remission group, comprising of MZ twin pairs discordant for asthma at age 10 but concordant for no asthma phenotype at 18 (*n* = 20 twin pairs), and (ii) concordant unaffected MZ twin pairs where neither twin had asthma at both ages 10 and 18 (*n* = 19 twin pairs). At nine of the ten top-ranked DMPs, average within-twin differences in DNA methylation were significantly different between groups (see Fig. 2b), with post hoc pairwise comparisons indicating that average within-twin differences in DNA methylation are significantly larger at these top-ranked DMPs in the persistent-asthma-discordant twins compared to both the asthma-remission twin sub-group and twins concordant for no asthma. GO term enrichment analysis was undertaken on persistent age-10 asthma DMPs (nominal *P* < 0.001, see Additional file 2: Table S3 for a list of loci included in the analysis). We identified 142 enriched (nominal *P* < 0.05) terms (see Additional file 2: Table S4), including categories related to interleukin-2 (IL-2) processes (GO:0042094 (*P* = 0.0009), GO:0032623 (*P* = 0.0025)) and type I interferon production (GO:0032480 (*P* = 0.0039)). Of note, both IL-2 and interferon pathways play an important role in cell-mediated immunity [32, 33] and have previously been implicated in asthma pathogenesis [34, 35].

**Table 2:** The top-ranked DMPs in age-10 MZ twins persistently discordant for asthma

	Age 10					Age 5							
Probe ID	Co-twin mean	Asthma mean	Mean ∆*β*	*P* value	Empirical *P* value	Mean ∆*β*	*P* value	Relation to CpG island	Gene region feature category (UCSC)	Hg19	Illumina gene annotation	Probe type	Gene annotation from GREAT (distance from TSS)
cg23603194	0.289	0.180	−0.109	6.87E-06	0.0009	0.056	0.0519	N_Shore	Intergenic	Chr1:221060753		II	HLX (+8011), DUSP10 (+854762)
cg27385757	0.353	0.286	−0.067	2.16E-05	0.0012	0.029	0.1966	S_Shore	TSS1500; 3′UTR	Chr6:30656024	KIAA1949; NRM	II	PPP1R18 (−932)
cg06483698	0.805	0.848	0.043	2.64E-05	0.0007	−0.006	0.6847	N_Shore	Body	Chr7:6310290	CYTH3	II	CYTH3 (+1951), USP42 (+165741)
cg05895618	0.559	0.655	0.096	2.69E-05	0.0007	−0.026	0.5367		5′UTR	Chr11:19222395	CSRP3	II	CSRP3 (+1193), ZDHHC13 (+83704)
cg14868530	0.081	0.098	0.017	3.36E-05	0.0005	−0.008	0.0351	Island	5′UTR; 1stExon	Chr15:73976679	CD276	I	CD276 (+58)
cg21304454	0.530	0.392	−0.138	3.59E-05	0.0009	0.070	0.1223	N_Shelf	Body	Chr1:17302859	MFAP2	II	MFAP2 (+4313), CROCC (+54415)
cg11998205	0.853	0.806	−0.047	4.28E-05	0.0017	−0.033	0.0361	S_Shelf	Body	Chr19:48840527	TMEM143	I	EMP3 (+11899), TMEM143 (+26658)
cg18513344	0.210	0.137	−0.072	4.39E-05	0.0007	−0.022	0.3459		Body	Chr3:195531298	MUC4	II	MUC4 (+7545), MUC20 (+83546)
cg08657206	0.256	0.221	−0.035	5.55E-05	0.0009	−0.005	0.7971	N_Shore	Intergenic	Chr1:180197901		I	LHX4 (−1531)
cg19865472	0.897	0.844	−0.052	5.90E-05	0.0004	−0.065	0.1373	Island	3′UTR	Chr19:617133	HCN2	I	POLRMT (+16434), HCN2 (+27241)
cg26280666	0.0976	0.0717	−0.0259	6.13E-05	0.0009	−0.0115	0.4006	N_Shore	Body	Chr8:72755568	MSC	II	EYA1 (−486590), MSC (+1162)
cg13512987	0.5252	0.6452	0.1200	6.77E-05	0.0005	−0.0438	0.1137		5′UTR; 1stExon	Chr12:6898785	CD4	II	CD4 (+148)
cg24664347	0.7364	0.8161	0.0797	7.51E-05	0.0006	−0.0624	0.0007	S_Shore	TSS1500	Chr8:86841010	REXO1L2P	II	REXO1L1 (−51705), PSKH2 (+240840)
cg25346117	0.6694	0.7450	0.0756	8.06E-05	0.0004	0.0030	0.8688		Body	Chr1:116923256	ATP1A1	II	ATP1A1 (+7462), CD58 (+190458)
cg09587549	0.2320	0.2647	0.0327	9.75E-05	0.0005	−0.0006	0.9742	Island	5′UTR; 1stExon	Chr16:31214126	PYCARD	I	PYCARD (−30)
cg03225548	0.7651	0.6807	−0.0844	9.90E-05	0.0004	−0.0014	0.9638	N_Shelf	Body; 5′UTR	Chr18:71811234	FBXO15	II	TIMM21 (−4511), FBXO15 (+3865)
cg17130754	0.7563	0.6967	−0.0597	9.99E-05	0.0004	0.0478	0.0739		Intergenic	Chr10:3536341		II	PITRM1 (−321309), KLF6 (+291131)

Ranked by *P* value. Empirical *P* value = (number of permutations which are at least as significant as the true result (*P* < 0.0001) divided by the number of permutations performed (*n* = 8192))

*DMPs* differentially methylated positions, *MZ* monozygotic, *GREAT* Genomic Regions Enrichment of Annotations Tool, *TSS* transcription start site, *UCSC* University of California Santa Cruz genome browser

### Longitudinal DNA methylation changes in persistent-asthma MZ twins

DNA methylation at the top-ranked age-10 persistent-asthma DMPs (*P* < 0.0001) could be examined in matched buccal cell DNA collected at age 5 for 11 of the 13 persistently discordant MZ twin pairs (2 pairs lacked age-5 DNA methylation data). Although two of the top-ranked probes (cg14868530 (annotated to *CD276*) and cg11998205 (annotated to *TMEM143*)) were significantly differentially methylated in the same direction between affected twins and their unaffected co-twin at age 5 (*P* = 0.035 and *P* = 0.036, respectively), no significant differences at age 5 were observed for the other top-ranked loci, and there was no overall correlation in within-twin differences at ages 5 and 10 for the top-ranked persistent-asthma DMPs. These findings suggest that epigenetic variation associated with asthma persisting through adolescence is not detectable in very early childhood. Within our persistent-asthma sub-group, we therefore next examined persistent-asthma-associated intra-individual changes in DNA methylation between ages 5 and 10. For each probe, we calculated the change in DNA methylation from age 5 to age 10 (longitudinal ∆*β*) for each individual and examined the difference in longitudinal ∆*β* between affected twins and their unaffected co-twin. The top-ranked probe (cg26090469), located in *AFF4*, became significantly hypomethylated from age 5 to age 10 in twins who developed persistent asthma, compared to their unaffected co-twin (*P* = 1.41E-06, Table 3). GO term enrichment analysis was performed for the genes associated with the asthma-associated (nominal *P* < 0.001) intra-individual changes in DNA methylation identified between ages 5 and 10 (see Additional file 2: Table S5 for a list of genes included). This analysis identified 90 enriched (nominal *P* < 0.05) terms (see Additional file 2: Table S6), with the top-ranked categories being related to activin receptor signalling (GO:0032926 (*P* = 0.0006), GO:0032925 (*P* = 0.005)) and epithelium development (GO:0060429 (*P* = 0.0006)).

**Table 3:** The top-ranked CpG sites which show changes in DNA methylation levels overtime in the discordant MZ twins

Probe ID	Co-twin mean	Asthma mean	Mean ∆*β*	*P* value	Hg19	Relation to CpG island	Gene region feature category (UCSC)	Illumina gene annotation	Probe type	Gene annotation from GREAT (distance from TSS)
cg26090469	−0.0122	0.0341	0.0463	1.41E-06	Chr5:132271449		Body	AFF4	II	AFF4 (+27904), LEAP2 (+62092)
cg02181624	0.1204	−0.0649	−0.1854	1.68E-06	Chr6:168716070	N_Shelf	Body	DACT2	II	FRMD1 (−236232), DACT2 (+4331)
cg17199800	−0.0902	0.0620	0.1522	9.47E-06	Chr7:96632777	N_Shore	Body	DLX6AS	II	DLX6 (−2512)
cg21875946	0.0892	−0.0276	−0.1168	1.88E-05	Chr1:206663985		Body	IKBKE	II	RASSF5 (−16893), IKBKE (+20400)
cg15691003	0.0082	−0.0205	−0.0287	3.26E-05	Chr1:22351864	N_Shore	Body; TSS200	HSPC157	II	CDC42 (−27255), CELA3B (+48447)
cg02836325	−0.1285	−0.0153	0.1132	3.37E-05	Chr17:76403955		Body	PGS1	II	PGS1 (+29221), DNAH17 (+169520)
cg16789707	−0.0958	0.0404	0.1362	4.33E-05	Chr16:88767723	S_Shelf	Body	RNF166	II	SNAI3 (−14842), RNF166 (+5105)
cg04313071	0.1063	−0.0249	−0.1312	4.33E-05	Chr14:102685619		Body	WDR20	II	HSP90AA1 (−79534), MOK (+85911)
cg23847069	0.1153	−0.0057	−0.1210	4.79E-05	Chr17:2702204		Body	RAP1GAP2	I	RAP1GAP2 (+2473), OR1D5 (+264696)
cg24293948	0.0646	−0.0729	−0.1375	5.04E-05	Chr18:61670413	S_Shore	Intergenic		II	SERPINB8 (+33151), LINC00305 (+95460)
cg06367321	0.1110	−0.0015	−0.1125	5.14E-05	Chr16:30125232		TSS200; TSS1500	LOC100271831; GDPD3	I	GDPD3 (−355)
cg06655665	−0.1603	−0.0359	0.1243	5.45E-05	Chr2:38607629	S_Shelf	Intergenic		II	ATL2 (−3198)
cg25406011	0.1312	−0.0175	−0.1487	6.04E-05	Chr11:1254421	Island	Body	MUC5B	II	MUC5B (+10127), TOLLIP (+76470)
cg25494605	0.0873	−0.0652	−0.1525	6.55E-05	Chr5:173472097		TSS1500	HMP19	II	HMP19 (−626)
cg24664347	−0.1381	0.0087	0.1468	6.88E-05	Chr8:86841010	S_Shore	TSS1500	REXO1L2P	II	REXO1L1 (−51705), PSKH2 (+240840)
cg18184910	0.2047	0.0402	−0.1644	7.10E-05	Chr6:47624247		TSS200	GPR111	II	GPR111 (−78)
cg00840341	0.1094	0.0131	−0.0963	7.60E-05	Chr16:17609453		Intergenic		II	XYLT1 (−44716), AK310228 (+825170)
cg14141399	0.2139	−0.0312	−0.2451	7.66E-05	Chr19:52228048		TSS1500	HAS1	II	HAS1 (−828)
cg14179581	−0.1193	0.0758	0.1951	7.74E-05	Chr1:50881502	Island	Intergenic		II	DMRTA2 (+7616), ELAVL4 (+306909)
cg21209859	−0.0892	0.0351	0.1242	7.78E-05	Chr1:167682516	N_Shore	Intergenic		II	CREG1 (−159461), MPZL1 (−8670)
cg21187770	0.1265	−0.0432	−0.1697	8.57E-05	Chr2:26205876	S_Shore	TSS1500	KIF3C	I	KIF3C (−434)
cg01382502	−0.1489	−0.0279	0.1210	8.96E-05	Chr16:22252504		Body	EEF2K	II	POLR3E (−56236), EEF2K (+34913)
cg09900436	−0.1813	0.0363	0.2175	8.96E-05	Chr20:58630954		TSS200	C20orf197	II	CDH26 (+97484)
cg16606561	0.2193	0.0631	−0.1562	9.72E-05	Chr20:824641	N_Shore	5′UTR; TSS1500	FAM110A	II	FAM110A (+10286), ANGPT4 (+72318)
cg15048437	0.0067	−0.0159	−0.0226	9.85E-05	Chr19:37019614	Island	TSS1500	ZNF260	I	ZNF260 (−367)

Ranked by *P* value

*DMPs* differentially methylated positions, *MZ* monozygotic, *GREAT* Genomic Regions Enrichment of Annotations Tool, *TSS* transcription start site, *UCSC* University of California Santa Cruz

## Discussion

In this study, we first assessed genome-wide patterns of DNA methylation in buccal cell samples collected from 37 asthma-discordant MZ twin pairs at age 10. We identified a number of asthma-associated DMPs, characterized by consistent within-pair differences in DNA methylation. The top-ranked DMP (cg03284554) is located upstream of *HGSNAT*, which has been previously shown to be down-regulated in paediatric allergic asthma [27]. We next stratified our analysis by assessing DNA methylation differences in a sub-group of MZ twin pairs who remained persistently discordant for asthma 8 years later, at age 18. The top-ranked DMP associated with persisting asthma (cg23603194) is located 8 kb downstream of the transcriptional start site (TSS) of the *HLX* gene, which has been previously implicated in childhood asthma [29, 30]. Moreover, a number of additional probes (cg18788664, cg10975889, cg15730491) located in the proximity of the *HLX* gene were significantly differentially methylated in affected twins compared to their unaffected co-twin. Although the majority of these probes are located >8 kb downstream of the *HLX* TSS, their proximity to annotated CpG islands (see Fig. 3) suggests that they reside in potential regulatory regions. *HLX* has been identified as an important regulator of TH_1_ differentiation and a suppressor of TH_2_ commitment, mediating pathways that are known to be perturbed in asthma [29, 36]. The functional relevance of DNA methylation changes downstream of the *HLX* gene warrants further investigation.

Several other DMPs identified in the persistent-asthma sub-group analysis are located in the vicinity of genes that have previously been implicated in asthma. For example, cg13512987, located in the first exon of the cluster of differentiation 4 (*CD4*) gene, was significantly hypermethylated in asthma-affected twins compared to their unaffected co-twin. CD4 is a glycoprotein found on the surface of immune cells such as T helper cells, monocytes, macrophages and dendritic cells [37]. CD4+ T cells are increased in broncho–alveolar lavage fluid and bronchial biopsy specimens from asthmatic patients [38]. Similarly, cg14868530, which is hypermethylated in affected twins compared to their unaffected co-twin, is located in the first exon of *CD276*, a gene important for T cell proliferation [39]. Furthermore, cg18513344, which we found to be hypomethylated in affected twins, is associated with *MUC4*, a gene that encodes an airway mucin protein—a major constituent of mucus [40]. Interestingly, evaluation of airway biopsies from asthmatic patients have observed higher *MUC4* mRNA levels compared to normal healthy controls [41]. DNA methylation studies in other complex phenotypes report [42–44] similarly small absolute (<10 %) differences in DNA methylation to those observed in this study. The DNA methylation values reported in this study represent aggregate values across all cells from a given sample. A shift in DNA methylation of 5–10 % indicates that ~5–10 % of DNA molecules in the population assessed become fully methylated (or unmethylated) at that locus, with potentially large functional consequences in that specific cellular sub-population. Finally, GO analysis of differentially methylated loci associated with a persistent-asthma phenotype revealed an enrichment of immune-related pathways including IL-2 and interferon type I signalling pathways which have previously been implicated in asthma pathogenesis [34, 35]. Together, our data suggest that epigenetic variation is associated with childhood asthma, and that methylomic differences may potentially be useful markers for predicting the persistence of asthma at age 18.

A recent EWAS analysis identified robust DNA methylation changes at several loci associated with imunnunoglobulin E (IgE) levels, which are known to correlate positively with allergic diseases such as asthma [22]. Two of the 69 IgE-associated DMPs (cg11398517 (*P* = 0.008), cg16522484 (*P* = 0.04)) were nominally significantly associated with asthma in our primary asthma-discordant cohort (37 twin pairs). Also, 5 of the 69 IgE-associated DMPs were nominally associated with asthma in our persistently discordant MZ twins (cg08404225 (*P* = 0.003), cg05215575 (*P* = 0.014), cg09676390 (*P* = 0.019), cg25494227 (*P* = 0.02), cg17749520 (*P* = 0.029)). These DMPs were not associated with asthma in twin pairs where the affected twin had remission of symptoms by age 18, although several IgE-associated DMPs were cg17890764 (*P* = 0.003), cg07374928 (*P* = 0.01) and cg11398517 (*P* = 0.028); these DMPs may be associated with IgE levels in childhood asthma which remits in early adulthood. Although our findings require replication in larger independent prospective asthma cohorts, the data suggest that prognostic DNA methylation markers of persistent asthma may be identified. It would be interesting to combine epigenetic data with polygenic risk scores, which have previously been associated with life-course-persistent asthma [7].

DNA methylation at the top-ranked age-10 persistent-asthma DMPs (*P* < 0.0001) was examined in matched buccal cell DNA collected at age 5 for 11 of the 13 persistently discordant MZ twin pairs. Although there was no overall correlation in within-twin differences at ages 5 and 10 for the top-ranked persistent-asthma DMPs, we identified a number of age-10 DMPs that were also nominally significantly differentially methylated in the twins at age 5. These data suggest that DNA methylation of these loci could potentially represent early biomarkers for persistent asthma. We next examined asthma-associated intra-individual changes in DNA methylation between ages 5 and 10. The top-ranked longitudinal DMP (cg26090469), which was significantly hypomethylated from age 5 to age 10 in twins with persistent asthma compared to their unaffected co-twin, is located in the *AFF4* gene. Research suggests that AFF4, an important scaffold protein, may play a role in viral gene expression [45]. Similarly, cg25406011, located in exon 18 of the *MUC5B* gene, was significantly hypomethylated from age 5 to age 10 in twins with persistent asthma compared to their unaffected co-twin. *MUC5B* codes for the major gel-forming mucin in mucus. Recently, it has been shown that mouse Muc5b is required for mucociliary clearance, for controlling infections in the airways and for immune homeostasis in mouse lungs [46]. Our data suggests that site-specific longitudinal intra-individual DNA methylation changes in *MUC5B* may play a role in the pathogenesis and development of asthma. Genes annotated to the most DMPs between ages 5 and 10 in twins who develop persistent asthma highlighted significant GO term enrichment for activin receptor signalling and epithelium development. Notably, activin signalling has previously been implicated in airway remodelling in asthma [47].

Despite the power of the discordant MZ twin approach for epigenetic epidemiology, there are several limitations to this study. First, an event history calendar was used to record children’s history of chronic health conditions at ages 5 and 10 and a set of seven asthma symptoms were assessed to determine asthma status at age 18. However, the questions used in this study are well established and have been validated and used previously in studies of asthma and other childhood phenotypes [48–51]. Second, our primary and secondary analyses utilized a small cohort of 74 twin children (37 MZ pairs) and 26 twin children (13 MZ twin pairs), respectively. Despite the modest sample size, the cases were exhaustive of asthma-discordant pairs in our birth cohort which has been shown to represent the UK population [52]. Nevertheless, our analyses were relatively underpowered to detect small changes in DNA methylation. Although no probe reaches Bonferroni-corrected levels of significance, DNA methylation studies in other complex phenotypes report similarly small absolute differences. Given the known non-independence of DNA methylation across the probes represented on the 450K array [53], it is likely that conventional methods of global statistical significance (which assume statistical independence of the multiple tests) are not appropriate for these analyses [54]. As an alternative approach, we therefore report empirical *P* values (generated by randomly re-assigning disease status and re-analysing the data) for each of the top-ranked probes using permutation testing [54]. After up to 10,000 permutations, each of our top-ranked probes was found to be significantly associated with asthma at an empirical *P* < 0.0009. Third, methylomic analysis was performed on DNA from buccal cells, which may not represent the primary target tissue involved in asthma. However, buccal cells are an easily accessible tissue that encompasses a portion of the upper airway and have previously been proposed as a surrogate for airway epithelial cells [19]. Due to the limited availability of buccal cell DNA, technical validation, using bisulfite pyrosequencing, of our top-ranked DMPs was not performed in this study. Of note, however, we and others have previously validated small changes in DNA methylation identified from the 450K array using pyrosequencing, highlighting the reliability of the 450K array [43, 55–57]. Our analysis of asthma-concordant MZ twin pairs highlights the specificity of the differences we observed in disease-discordant twin pairs. Additionally, buccal cell RNA was not available for our MZ twin cohort and thus we were unable to directly examine the functional relevancy of asthma-associated DMPs on gene expression. One final caveat in this study is that chorionicity data were not available on these twins, a potential limitation given that whether or not MZ twins share a placenta may influence epigenomic and transcriptional differences mediated by subtle differences in the prenatal environment [58].

## Conclusions

In summary, we have identified DNA methylation differences associated with childhood asthma in peripheral DNA samples from discordant MZ twin pairs. The top-ranked persistent-asthma-associated DMP (cg23603194) identified in this study is downstream of the *HLX* gene that has been previously implicated in the pathogenesis of asthma. Our data suggest that differences in DNA methylation associated with childhood asthma which persists into early adulthood are distinct from those associated with childhood asthma which remits before early adulthood.

## Methods

### Study cohort

Participants were recruited from the Environmental Risk (E-Risk) longitudinal twin study, which tracks the development of a birth cohort of 1116 British twin pairs (*n* = 2232 individuals). The sample was drawn from a larger birth register of twins born in England and Wales in 1994–1995 [59]. Full details about the sample are reported elsewhere [60]. The sample includes 55 % MZ twin pairs. Sex is evenly distributed within zygosity (49 % male). The sample was originally assessed when the twins were aged 5, and follow-up home visits took place when the children were aged 7 (98 % participation), 10 (96 % participation), 12 (96 % participation) and 18 years (93 % participation). The Joint South London and Maudsley and the Institute of Psychiatry Research Ethics Committee approved each phase of the study. Prior to age 18, parents gave informed consent and children gave assent; at age 18, the participants themselves gave informed consent.

### Measures

At ages 5 and 10, information was collected from mothers about each child’s asthma using an event history calendar for recording children’s history of chronic health conditions. Methodological studies document that event history calendars improve accuracy of health information over conventional questionnaire instruments [48, 49]. A sub-sample of 56 Caucasian families (112 children, 56 twin pairs) was used for this study, comprising families who (a) had MZ twins, and (b) were discordant for asthma at age 10 (37 twin pairs) or (c) both twins did not have asthma (19 twin pairs). All MZ twins were Caucasian and born in the UK. Buccal cell samples were obtained from children during home visits at ages 5 and 10 (see Additional file 2: Table S7 for further details). Genomic DNA was extracted from buccal cells using a standard procedure [61].

When the children were 18 years old, asthmatic symptoms were further assessed in a private individual interview conducted by trained professionals. Three questions were taken from the European Community Respiratory Health Survey (ECRHS) II screening questionnaire (www.ecrhs.org) [51] related to asthma (“Have you been woken by an attack of shortness of breath at any time in the last 12 months?”, “Have you had an attack of asthma in the last 12 months?”, “Are you currently taking any medicine (including inhalers, aerosols or tablets) for asthma?”). A dichotomous variable representing children who reported no to all three questions and those who reported at least one definite asthma symptom was created. Follow-up phenotypic information was available for 36/37 discordant twin pairs at age 18. Thirteen MZ twin pairs discordant at age 10 remained discordant for asthma at age 18. Twenty MZ twin pairs discordant at age 10 became concordant unaffected at age 18, and three MZ twin pairs became concordant affected at age 18.

### Methylomic profiling

Genomic DNA (500 ng) was treated with sodium bisulfite using the EZ-96 DNA Methylation Kit (Zymo Research, CA, USA) following the manufacturers’ protocol. DNA methylation was quantified using the Infinium HumanMethylation450 BeadChip array (Illumina, Inc., San Diego, CA) as previously described [24]. Each twin pair was processed together on the same array to avoid batch effects. Genome Studio software (Illumina, Inc.) was used to extract signal intensities for each probe and perform initial quality control. Further quality control checks, quantile normalization and separate background adjustment of methylated and unmethylated intensities of type I and II probes were employed using the wateRmelon package in R (available from the bioconductor repository www.bioconductor.org) [24]. Samples with 5 % of sites with a detection *P* value >.05 or a bead count <3 in 5 % of samples were removed from the analysis. Non-specific probes and probes on the X and Y chromosomes were removed [62, 63]. The final analyses included 391,554 probes and included 31 asthma-discordant MZ pairs (62 individual samples) at age 5, 37 MZ pairs (74 individual samples) at age 10, and 19 MZ (38 individual samples) age-10 concordant unaffected pairs. Polymorphic single nucleotide polymorphism control probes (*n* = 65) located on the array were used to confirm monozygosity for all twin pairs included in the final analysis.

### Data analyses

All statistical analyses were performed using R statistical package (version 3.1.1). Our primary analyses employed a paired *t* test to identify DMPs between affected twins (children who were reported as having a history of asthma) and unaffected (children who had no reported history of asthma) twin children using DNA collected at age 10. Next, we identified a persistent-asthma sub-group (13 MZ twin pairs) of asthma-discordant MZ twins at age 10 who remained discordant for asthma at age 18. A paired *t* test was employed to identify DMPs between twins discordant (at both ages 10 and 18) for asthma using DNA collected at age 10. Probes were ranked according to *P* value. Analyses were repeated for buccal samples collected at age 5. The Beta value (*β*) is a ratio between methylated probe intensity and total probe intensities (sum of methylated and unmethylated probe intensities) and ranges from 0 to 1. Within the persistent-asthma sub-group, intra-individual changes in DNA methylation from ages 5 to 10 were calculated (longitudinal ∆*β*) and the difference in longitudinal ∆*β* between persistent-asthma-affected twins and their unaffected co-twin was examined using a paired *t* test. An empirical *P* value was calculated for top-ranked DMPs (*P* < 0.0001) in both the asthma-discordant (*n* = 37 twin pairs) and the persistent-asthma sub-group (*n* = 13 twin pairs) by first randomly assigning twin status (affected/unaffected) and performing a paired *t* test. This was repeated for up to 10,000 permutations. An empirical *P* value was calculated by dividing the number of permutations which are at least as significant as the true result (*P* < 0.0001) by the number of permutations performed.

The specificity of our persistent-asthma-associated DMPs was determined by examining within-twin DNA methylation differences at these loci in (i) an asthma-remission sub-group (twins discordant for asthma at age 10 but the affected twin had remitted at 18, *n* = 20 twin pairs) and (ii) concordant unaffected control MZ twins, with no history of asthma in either twin (*n* = 19 twin pairs). For the asthma-remission sub-group, the average within-twin beta difference (∆*β*) was calculated, at these loci, by taking the average within-twin beta difference (beta value of asthma-affected twin (age 10) − beta value of unaffected co-twin (age 10)). For the concordant unaffected control MZ twins, the average within-twin beta difference, at these loci, was calculated using permutation testing (*n* = 1000). Briefly, concordant unaffected twin pairs were randomly assigned a dichotomous variable (0 (twin 1) or 1 (twin 2)) and the within-twin beta difference (beta value of twin 1 − beta value of twin 2) was calculated for each twin pair at each probe. Next, the average within-twin beta difference (within-twin ∆*β*) was calculated across all twin pairs at each probe, and this was repeated 1000 times. The average within-twin ∆*β* was calculated by taking the average of the permutated within-twin ∆*β* values obtained at each DMP of interest. The average within-twin ∆*β* was then compared to (i) the average within-twin ∆*β* calculated from the persistent-asthma-discordant MZ twin pairs and (ii) the average within-twin ∆*β* calculated from the asthma-remission group at each probe of interest using a one-way analysis of variance (ANOVA). Post hoc pairwise comparisons (pairwise *t* test) were performed to identify which groups were significantly different from each other.

### Gene ontology term enrichment analysis

Gene ontology (GO) term enrichment analysis was performed on genes annotated (Illumina UCSC gene annotation) to age-10 DMPs, age-10 persistent-asthma DMPs and longitudinal DMPs (nominal *P* < 0.001), respectively, using the R package GOseqv1.18.1 (downloaded from Bioconductor [64]). GOseq can be used to correct for the number of Illumina 450K probes in each gene during GO term enrichment analysis. The number of probes per gene was calculated in our final dataset to create a probability weighting function, which was then used in the GO term enrichment analysis.

## Additional files

Additional file 1: Figure S1. Flow chart describing the methodological approach used in this study. Our analyses focused on identifying differentially methylated positions (DMPs) associated with asthma in (A) all asthma-discordant MZ twins at age 10 and (B) a sub-group with persistent asthma who were discordant for asthma at age 10 and also at age 18. Using DNA previously collected at age 5, we (C) subsequently assessed longitudinal changes in DNA methylation (between ages 5 and 10) in persistent-asthma-discordant MZ twins. Finally, we (D) examined epigenetic variation at top-ranked persistent-asthma-associated DMPs in an asthma-remission group, comprising of MZ twin pairs discordant for asthma at age 10 but concordant for no asthma phenotype at 18 and concordant unaffected MZ twin pairs where neither twin had asthma at both ages 10 and 18. Additional file 2: Tables S1–S7.
**Table S1.** The top-ranked DMPs (*P* < 0.001) in discordant MZ twin pairs at age 10. **Table S2.** Gene ontology enrichment analysis for age-10 asthma-associated DMPs. **Table S3.** The top-ranked DMPs at age 10 in persistent-asthma-discordant MZ twins (between 10 and 18 years). **Table S4.** Gene ontology enrichment analysis for persistent-asthma age-10 DMPs. **Table S5.** The top-ranked CpG sites which show changes in DNA methylation levels between 5 and 10 years of age in the asthma-discordant MZ twins. **Table S6.** Gene ontology enrichment analysis of longitudinal DMPs between 5 and 10 years of age. **Table S7.** Monozygotic twin group details.
