# Supplementary figures and images for: Methylomic markers of persistent childhood asthma: a longitudinal study of asthma-discordant monozygotic twins
Source: Clin Epigenetics. 2015 Dec 18;7:130. doi: 10.1186/s13148-015-0163-4 (PMC4684622; doi:10.1186/s13148-015-0163-4)

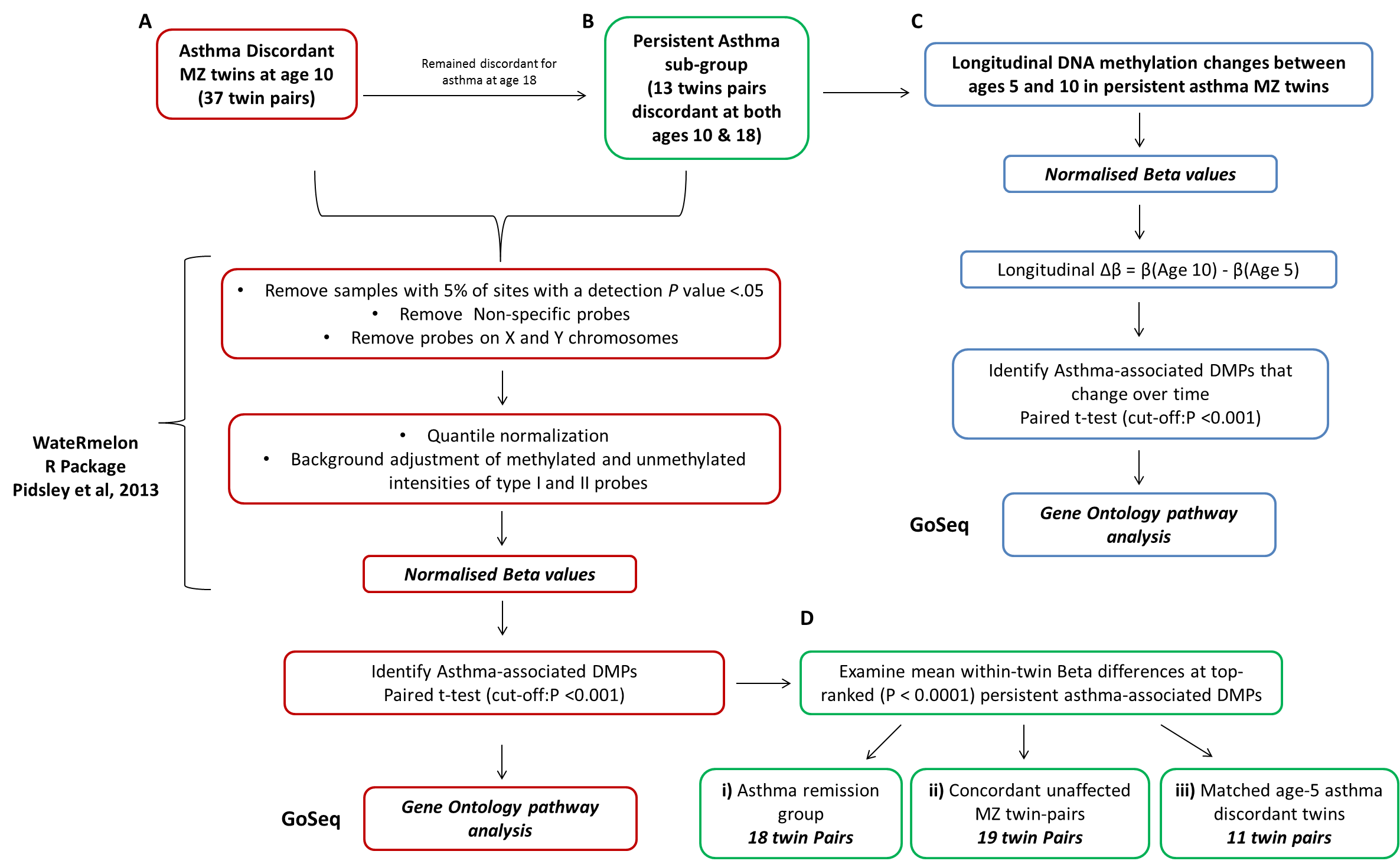

Supplement: Additional file 1: Figure S1. — Flow chart describing the methodological approach used in this study. Our analyses focused on identifying differentially methylated positions (DMPs) associated with asthma in (A) all asthma-discordant MZ twins at age 10 and (B) a sub-group with persistent asthma who were discordant for asthma at age 10 and also at age 18. Using DNA previously collected at age 5, we (C) subsequently assessed longitudinal changes in DNA methylation (between ages 5 and 10) in persistent-asthma-discordant MZ twins. Finally, we (D) examined epigenetic variation at top-ranked persistent-asthma-associated DMPs in an asthma-remission group, comprising of MZ twin pairs discordant for asthma at age 10 but concordant for no asthma phenotype at 18 and concordant unaffected MZ twin pairs where neither twin had asthma at both ages 10 and 18. [file 13148_2015_163_MOESM1_ESM.tif]
