# Supplementary material for: Methylomic markers of persistent childhood asthma: a longitudinal study of asthma-discordant monozygotic twins
Source: Clin Epigenetics. 2015 Dec 18;7:130. doi: 10.1186/s13148-015-0163-4 (PMC4684622; doi:10.1186/s13148-015-0163-4)
Supplement: Additional file 2: Tables S1–S7. — Table S1. The top-ranked DMPs (P < 0.001) in discordant MZ twin pairs at age 10. Table S2. Gene ontology enrichment analysis for age-10 asthma-associated DMPs. Table S3. The top-ranked DMPs at age 10 in persistent-asthma-discordant MZ twins (between 10 and 18 years). Table S4. Gene ontology enrichment analysis for persistent-asthma age-10 DMPs. Table S5. The top-ranked CpG sites which show changes in DNA methylation levels between 5 and 10 years of age in the asthma-discordant MZ twins. Table S6. Gene ontology enrichment analysis of longitudinal DMPs between 5 and 10 years of age. Table S7. Monozygotic twin group details. [file 13148_2015_163_MOESM2_ESM.docx]

| **Supplementary Table S1. The top-ranked DMPs (P < 0.001) in discordant MZ twin pairs at age 10** | | | | | | | |  |  | |  | |  | |  | |  | | |
| --- | --- | --- | --- | --- | --- | --- | --- | --- | --- | --- | --- | --- | --- | --- | --- | --- | --- | --- | --- |
| **Probe ID** | **Unaffected Co-Twin Mean** | **Asthma Twin Mean** | **Mean ∆β** | **P value** | **Hg19** | **Relation to CpG Island** | **Gene region feature category (UCSC)** | | | **SNPs in probe (+/- 10bp SBE)** | | **Illumina Gene Annotation** | | **Probe Type** | | **Gene Annotation from GREAT (Distance from TSS)** | | |  |
| cg03284554 | 0.178 | 0.218 | 0.041 | 6.87E-06 | Chr8:42994513 | N_Shore | TSS1500 | | |  | | HGSNAT | | II | | HGSNAT (-1078) | | |  |
| cg01496463 | 0.761 | 0.795 | 0.034 | 1.80E-05 | Chr12:3392647 |  | 3'UTR;3'UTR | | |  | | TSPAN9 | | II | | PRMT8 (-207777), TSPAN9 (+206127) | | |  |
| cg04373937 | 0.836 | 0.859 | 0.023 | 4.26E-05 | Chr14:30552825 |  |  | | | rs113008809 | |  | | II | | G2E3 (-475503), PRKD1 (-155927) | | |  |
| cg14688104 | 0.035 | 0.038 | 0.003 | 4.29E-05 | Chr8:99439441 | Island | 1stExon;5'UTR | | |  | | KCNS2 | | I | | KCNS2 (+192) | | |  |
| cg08158233 | 0.244 | 0.192 | -0.052 | 5.36E-05 | Chr8:37351056 |  |  | | |  | |  | | II | | ZNF703 (-202244), KCNU1 (+709215) | | |  |
| cg17269596 | 0.581 | 0.637 | 0.056 | 5.49E-05 | Chr1:244995112 | N_Shelf |  | | |  | |  | | II | | FAM36A (-3526) | | |  |
| cg14299157 | 0.813 | 0.756 | -0.058 | 5.59E-05 | Chr9:138132949 | N_Shelf |  | | |  | |  | | II | | PPP1R26 (-238698), OLFM1 (+165861) | | |  |
| cg21201551 | 0.747 | 0.779 | 0.032 | 5.90E-05 | Chr1:31632233 | S_Shelf |  | | |  | |  | | II | | PUM1 (-93670), NKAIN1 (+80500) | | |  |
| cg16414472 | 0.503 | 0.414 | -0.089 | 6.05E-05 | Chr11:68110105 |  | Body | | |  | | LRP5 | | I | | PPP6R3 (-118080), LRP5 (+29998) | | |  |
| cg01431063 | 0.057 | 0.072 | 0.014 | 8.92E-05 | Chr10:115860903 | Island |  | | |  | |  | | II | | TDRD1 (-78125), ADRB1 (+57098) | | |  |
| cg01048931 | 0.071 | 0.084 | 0.013 | 9.42E-05 | Chr7:55639931 | Island | Body | | |  | | VOPP1 | | I | | VOPP1 (+268) | | |  |
| cg24634420 | 0.053 | 0.047 | -0.005 | 0.00012 | Chr4:157892748 | Island | TSS1500 | | |  | | PDGFC | | I | | PDGFC (-203) | | |  |
| cg21013710 | 0.725 | 0.679 | -0.046 | 0.00016 | Chr11:15128655 |  |  | | |  | |  | | II | | INSC (-5314), CALCB (+33510) | | |  |
| cg14519115 | 0.132 | 0.115 | -0.017 | 0.00016 | Chr5:41510650 | Island | 1stExon;5'UTR | | |  | | PLCXD3 | | II | | PLCXD3 (+79) | | |  |
| cg01443755 | 0.563 | 0.617 | 0.054 | 0.00017 | Chr12:41221639 |  | 5'UTR | | |  | | CNTN1 | | II | | PDZRN4 (-360610), CNTN1 (+135282) | | |  |
| cg05593510 | 0.473 | 0.379 | -0.095 | 0.00018 | Chr5:176249224 | S_Shelf | Body | | |  | | UNC5A | | II | | UNC5A (+11665), HK3 (+77108) | | |  |
| cg26806713 | 0.796 | 0.843 | 0.047 | 0.00019 | Chr6:170451326 | N_Shore |  | | |  | |  | | II | | DLL1 (+148370), C6orf70 (+299606) | | |  |
| cg06597431 | 0.860 | 0.884 | 0.024 | 0.00019 | Chr12:117580007 |  |  | | |  | |  | | II | | TESC (-42757), FBXO21 (+48292) | | |  |
| cg01780466 | 0.049 | 0.056 | 0.007 | 0.00020 | Chr1:145477253 | Island | 1stExon | | |  | | LIX1L | | I | | LIX1L (+169) | | |  |
| cg20114113 | 0.772 | 0.809 | 0.037 | 0.00020 | Chr10:93391963 | N_Shore | Body | | |  | | PPP1R3C | | II | | PPP1R3C (+894) | | |  |
| cg07396904 | 0.901 | 0.873 | -0.028 | 0.00021 | Chr14:22103504 |  | TSS1500 | | |  | | OR10G2 | | II | | OR10G2 (-507) | | |  |
| cg10880599 | 0.700 | 0.748 | 0.048 | 0.00023 | Chr14:65408480 |  | Body | | |  | | GPX2 | | II | | GPX2 (+1050), CHURC1-FNTB (+27402) | | |  |
| cg01512265 | 0.204 | 0.164 | -0.040 | 0.00024 | Chr20:34359908 | Island | TSS200 | | |  | | PHF20 | | I | | PHF20 (-14) | | |  |
| cg16215033 | 0.872 | 0.843 | -0.029 | 0.00024 | Chr5:107196921 |  | 3'UTR | | |  | | FBXL17 | | II | | FER (-886601), EFNA5 (-190326) | | |  |
| cg24980904 | 0.169 | 0.138 | -0.031 | 0.00024 | Chr18:3248854 | S_Shore | 5'UTR | | |  | | MYL12A | | II | | MYL12B (-13256), MYL12A (+1327) | | |  |
| cg08900073 | 0.101 | 0.087 | -0.013 | 0.00024 | Chr11:118796721 | Island |  | | |  | |  | | II | | UPK2 (-30286), BCL9L (-15109) | | |  |
| cg17329292 | 0.135 | 0.110 | -0.025 | 0.00025 | Chr1:33895890 | N_Shore |  | | |  | |  | | II | | PHC2 (-54697), ZSCAN20 (-42341) | | |  |
| cg17967292 | 0.293 | 0.343 | 0.050 | 0.00025 | Chr11:42546581 |  |  | | |  | |  | | II | | API5 (-786923) | | |  |
| cg23548163 | 0.483 | 0.396 | -0.087 | 0.00026 | Chr19:46807119 | Island | 5'UTR;Body | | |  | | HIF3A | | II | | PPP5C (-43131), HIF3A (+6817) | | |  |
| cg10825530 | 0.287 | 0.250 | -0.037 | 0.00026 | Chr11:16023708 |  | Body | | |  | | SOX6 | | II | | SOX6 (+400704), INSC (+889739) | | |  |
| cg07799277 | 0.355 | 0.407 | 0.051 | 0.00028 | Chr5:98368443 |  |  | | |  | |  | | II | | CHD1 (-106206) | | |  |
| cg07095945 | 0.920 | 0.905 | -0.015 | 0.00028 | Chr15:85415281 |  | 3'UTR | | |  | | ALPK3 | | II | | SLC28A1 (-12631), ALPK3 (+55371) | | |  |
| cg26962002 | 0.911 | 0.930 | 0.019 | 0.00028 | Chr19:54963333 | S_Shelf | Body | | |  | | LENG8 | | I | | LENG8 (+3269), LENG9 (+11560) | | |  |
| cg15658784 | 0.181 | 0.147 | -0.034 | 0.00028 | Chr11:2951243 | Island | TSS1500 | | |  | | PHLDA2 | | I | | PHLDA2 (-594) | | |  |
| cg16928046 | 0.825 | 0.871 | 0.046 | 0.00029 | Chr17:45812243 | S_Shore | Body | | |  | | TBX21 | | II | | TBX21 (+1634), OSBPL7 (+86903) | | |  |
| cg16101278 | 0.087 | 0.104 | 0.017 | 0.00029 | Chr6:6648823 |  | Body | | | rs74598970 | | LY86 | | II | | RREB1 (-459262), LY86 (+59890) | | |  |
| cg20557069 | 0.708 | 0.645 | -0.063 | 0.00029 | Chr21:47646604 | N_Shore | Body | | |  | | LSS | | II | | C21orf56 (-42232), LSS (+2133) | | |  |
| cg02487026 | 0.088 | 0.072 | -0.016 | 0.00031 | Chr2:15701516 | Island | TSS200 | | |  | | NBAS | | II | | DDX1 (-30253) | | |  |
| cg25152058 | 0.854 | 0.803 | -0.051 | 0.00031 | Chr8:134222322 |  | Body | | |  | | WISP1 | | II | | WISP1 (+19041), NDRG1 (+87224) | | |  |
| cg02530860 | 0.470 | 0.510 | 0.039 | 0.00031 | Chr8:144371537 | Island |  | | | rs186848198 | |  | | II | | ZNF696 (-2021) | | |  |
| cg18850124 | 0.858 | 0.794 | -0.065 | 0.00031 | Chr12:123481960 | Island | Body | | |  | | PITPNM2 | | II | | ARL6IP4 (+17081), PITPNM2 (+113074) | | |  |
| cg16240781 | 0.711 | 0.754 | 0.043 | 0.00031 | Chr2:45234379 | N_Shore | Body | | |  | | SIX2 | | II | | SIX2 (+2162), SIX3 (+65343) | | |  |
| cg03212129 | 0.079 | 0.064 | -0.014 | 0.00032 | Chr19:34895470 | Island | Body | | |  | | PDCD2L | | I | | PDCD2L (+168) | | |  |
| cg17394304 | 0.670 | 0.723 | 0.052 | 0.00033 | Chr3:187453973 | N_Shore | 5'UTR;1stExon;TSS1500 | | |  | | BCL6 | | II | | RTP2 (-33629), BCL6 (+9539) | | |  |
| cg24512138 | 0.299 | 0.268 | -0.031 | 0.00033 | Chr3:133465360 | Island | Body | | | rs8177187 | | TF | | I | | TF (+561) | | |  |
| cg07514505 | 0.065 | 0.082 | 0.017 | 0.00034 | Chr13:96743754 | Island | 1stExon | | |  | | HS6ST3 | | II | | HS6ST3 (+662) | | |  |
| cg14845609 | 0.852 | 0.893 | 0.041 | 0.00034 | Chr13:113376010 |  | Body | | |  | | ATP11A | | I | | MCF2L (-247524), ATP11A (+31368) | | |  |
| cg06935608 | 0.623 | 0.676 | 0.052 | 0.00035 | Chr8:26309176 | S_Shore |  | | |  | |  | | II | | PNMA2 (+62306), BNIP3L (+68654) | | |  |
| cg07538777 | 0.853 | 0.876 | 0.023 | 0.00036 | Chr19:5717584 | N_Shelf | Body | | |  | | LONP1 | | II | | TMEM146 (-3103), LONP1 (+2591) | | |  |
| cg21057473 | 0.796 | 0.830 | 0.033 | 0.00036 | Chr11:5346163 |  | TSS1500 | | | rs116511164 | | OR51B2 | | II | | OR51B2 (-582) | | |  |
| cg11480873 | 0.089 | 0.067 | -0.022 | 0.00036 | Chr1:205744417 | Island | 1stExon;5'UTR | | |  | | RAB7L1 | | II | | RAB7L1 (+192) | | |  |
| cg24228040 | 0.104 | 0.089 | -0.015 | 0.00036 | Chr22:50496857 | Island |  | | |  | |  | | I | | MLC1 (+26923), PIM3 (+142715) | | |  |
| cg25426285 | 0.162 | 0.145 | -0.017 | 0.00036 | Chr12:65564395 | Island | 1stExon;1stExon | | |  | | LEMD3 | | II | | MSRB3 (-108371), LEMD3 (+1045) | | |  |
| cg02731042 | 0.049 | 0.043 | -0.006 | 0.00037 | Chr18:6415118 | S_Shore | TSS1500 | | |  | | L3MBTL4 | | I | | L3MBTL4 (-209) | | |  |
| cg25587481 | 0.744 | 0.771 | 0.027 | 0.00037 | Chr2:46396825 |  | Body | | |  | | PRKCE | | II | | EPAS1 (-127715), PRKCE (+517783) | | |  |
| cg27520104 | 0.823 | 0.860 | 0.037 | 0.00038 | Chr10:3172590 |  | Body | | |  | | PFKP | | I | | PITRM1 (+42442), PFKP (+62879) | | |  |
| cg02135821 | 0.806 | 0.772 | -0.035 | 0.00038 | Chr14:94545678 | N_Shore | Body | | |  | | DDX24 | | II | | IFI27L1 (-1960), DDX24 (+1879) | | |  |
| cg01309141 | 0.468 | 0.527 | 0.059 | 0.00038 | Chr20:17205850 | N_Shore |  | | |  | |  | | II | | PCSK2 (-1746) | | |  |
| cg00526336 | 0.845 | 0.872 | 0.026 | 0.00040 | Chr1:230415902 | S_Shore | 3'UTR | | |  | | GALNT2 | | I | | PGBD5 (+97488), GALNT2 (+212947) | | |  |
| cg25772143 | 0.585 | 0.510 | -0.075 | 0.00040 | Chr1:110168021 |  | TSS200;Body;Body | | |  | | AMPD2 | | II | | GSTM4 (-30676), AMPD2 (+4746) | | |  |
| cg09753064 | 0.818 | 0.867 | 0.048 | 0.00040 | Chr20:42788303 | Island | Body | | |  | | JPH2 | | II | | JPH2 (+27914), TOX2 (+244812) | | |  |
| cg24881867 | 0.826 | 0.853 | 0.027 | 0.00040 | Chr3:10150487 | S_Shore | TSS1500 | | |  | | C3orf24 | | II | | BRK1 (-6845), FANCD2 (+82375) | | |  |
| cg23238734 | 0.886 | 0.829 | -0.057 | 0.00041 | Chr17:78661607 |  | Body | | |  | | RPTOR | | II | | CHMP6 (-304033), RPTOR (+142983) | | |  |
| cg07235935 | 0.429 | 0.476 | 0.047 | 0.00041 | Chr4:115964661 |  | Body | | |  | | NDST4 | | II | | NDST4 (+70370), UGT8 (+445051) | | |  |
| cg02758870 | 0.119 | 0.104 | -0.015 | 0.00042 | Chr4:100871523 | Island | TSS200 | | |  | | LOC256880;H2AFZ | | I | | DNAJB14 (-3645), H2AFZ (-12) | | |  |
| cg04890406 | 0.132 | 0.175 | 0.043 | 0.00043 | Chr3:48647370 | Island | TSS1500 | | |  | | UQCRC1 | | I | | UQCRC1 (-273) | | |  |
| cg19019038 | 0.120 | 0.133 | 0.013 | 0.00044 | Chr5:56469576 | Island | TSS200 | | |  | | GPBP1 | | I | | MIER3 (-221623), GPBP1 (-40324) | | |  |
| cg05264501 | 0.753 | 0.791 | 0.038 | 0.00044 | Chr13:36787952 | N_Shore | Body | | |  | | SOHLH2 | | II | | DCLK1 (-82439), CCDC169-SOHLH2 (+84039) | | |  |
| cg23289779 | 0.640 | 0.692 | 0.053 | 0.00045 | Chr8:26720681 | N_Shore | Body | | |  | | ADRA1A | | II | | ADRA1A (+2240), DPYSL2 (+348973) | | |  |
| cg09503596 | 0.329 | 0.266 | -0.063 | 0.00045 | Chr11:94883722 | Island |  | | |  | |  | | II | | ENDOD1 (+60749), SESN3 (+80523) | | |  |
| cg07472772 | 0.133 | 0.122 | -0.011 | 0.00045 | Chr20:31071948 | Island | TSS1500 | | |  | | C20orf112 | | I | | ASXL1 (+125802), C20orf203 (+167834) | | |  |
| cg14582400 | 0.381 | 0.466 | 0.085 | 0.00045 | Chr13:112726118 | N_Shore |  | | |  | |  | | II | | SPACA7 (-304532), SOX1 (+4206) | | |  |
| cg13702982 | 0.821 | 0.859 | 0.037 | 0.00046 | Chr9:89762972 | N_Shore | TSS1500 | | |  | | C9orf170 | | II | | DAPK1 (-349783), GAS1 (-200869) | | |  |
| cg22643760 | 0.814 | 0.848 | 0.035 | 0.00047 | Chr19:36746845 | Island |  | | |  | |  | | II | | ZNF565 (-41280), ZFP14 (+123259) | | |  |
| cg12014008 | 0.286 | 0.256 | -0.030 | 0.00048 | Chr10:48439443 | S_Shore | TSS1500 | | |  | | GDF10 | | II | | GDF10 (-306) | | |  |
| cg10370687 | 0.850 | 0.870 | 0.020 | 0.00048 | Chr16:89317353 | S_Shelf |  | | |  | |  | | II | | ZNF778 (+33243), ANKRD11 (+239615) | | |  |
| cg05435882 | 0.866 | 0.849 | -0.017 | 0.00049 | Chr16:88311214 | Island |  | | |  | |  | | II | | ZNF469 (-182664), BANP (+326177) | | |  |
| cg20033731 | 0.067 | 0.049 | -0.018 | 0.00051 | Chr12:2161371 | N_Shore | TSS1500 | | |  | | CACNA1 | | II | | CACNA1C (-1044) | | |  |
| cg20606489 | 0.896 | 0.916 | 0.020 | 0.00051 | Chr6:168630308 | Island |  | | |  | |  | | I | | FRMD1 (-150470), DACT2 (+90093) | | |  |
| cg15737398 | 0.065 | 0.077 | 0.012 | 0.00052 | Chr10:90639829 | Island | TSS200 | | |  | | STAMBPL1 | | II | | STAMBPL1 (-196) | | |  |
| cg06331333 | 0.304 | 0.259 | -0.045 | 0.00052 | Chr8:145938697 | Island |  | | |  | |  | | II | | ARHGAP39 (-99810), ZNF251 (+42272) | | |  |
| cg14153876 | 0.206 | 0.190 | -0.015 | 0.00052 | Chr2:157292773 | S_Shore | 5'UTR;TSS200 | | |  | | GPD2 | | I | | GPD2 (-126) | | |  |
| cg11593949 | 0.736 | 0.775 | 0.039 | 0.00052 | Chr7:45927735 | N_Shore | TSS1500 | | |  | | IGFBP1 | | II | | IGFBP1 (-223) | | |  |
| cg04396312 | 0.876 | 0.844 | -0.031 | 0.00053 | Chr10:88702403 | Island | Body | | |  | | MMRN2 | | I | | MMRN2 (+15021), BMPR1A (+186008) | | |  |
| cg00569808 | 0.805 | 0.843 | 0.038 | 0.00054 | Chr16:87467986 |  | Body | | |  | | ZCCHC14 | | II | | MAP1LC3B (+42186), ZCCHC14 (+57473) | | |  |
| cg27565368 | 0.770 | 0.723 | -0.047 | 0.00054 | Chr19:29705446 | S_Shore | TSS1500 | | |  | | UQCRFS1 | | II | | UQCRFS1 (-1311) | | |  |
| cg03143886 | 0.054 | 0.075 | 0.021 | 0.00054 | Chr18:22006473 | Island | TSS200 | | |  | | IMPACT | | I | | IMPACT (-135) | | |  |
| cg06520296 | 0.277 | 0.220 | -0.058 | 0.00054 | Chr8:37350981 |  |  | | |  | |  | | II | | ZNF703 (-202319), KCNU1 (+709140) | | |  |
| cg26141291 | 0.689 | 0.648 | -0.040 | 0.00055 | Chr8:142201025 |  | Body | | |  | | DENND3 | | II | | PTK2 (-189614), SLC45A4 (+37647) | | |  |
| cg25798013 | 0.106 | 0.089 | -0.017 | 0.00055 | Chr19:19754582 | S_Shore | TSS200 | | |  | | GMIP | | II | | GMIP (-128) | | |  |
| cg04652270 | 0.608 | 0.553 | -0.054 | 0.00055 | Chr16:892048 | N_Shelf |  | | |  | |  | | II | | GNG13 (-41316), LMF1 (+128935) | | |  |
| cg25091367 | 0.780 | 0.745 | -0.036 | 0.00056 | Chr4:7289507 |  | Body | | | rs189874537 | | SORCS2 | | II | | SORCS2 (+95134), PSAPL1 (+147192) | | |  |
| cg11451506 | 0.720 | 0.643 | -0.077 | 0.00058 | Chr2:100210257 | Island | Body | | |  | | AFF3 | | II | | REV1 (-103778), AFF3 (+511787) | | |  |
| cg06098146 | 0.885 | 0.846 | -0.039 | 0.00058 | Chr12:133202698 |  | Body | | |  | | POLE | | II | | P2RX2 (+7296), POLE (+61246) | | |  |
| cg08857936 | 0.729 | 0.687 | -0.041 | 0.00058 | Chr4:188539183 |  |  | | |  | |  | | II | | FAT1 (-894197), ZFP42 (-377741) | | |  |
| cg03283282 | 0.086 | 0.061 | -0.025 | 0.00058 | Chr4:40579075 |  | 5'UTR | | |  | | RBM47 | | II | | RBM47 (+52807), CHRNA9 (+241607) | | |  |
| cg05883713 | 0.807 | 0.775 | -0.032 | 0.00058 | Chr14:23963992 |  |  | | | rs189832530 | |  | | II | | NGDN (+25095), ZFHX2 (+56865) | | |  |
| cg25031205 | 0.864 | 0.895 | 0.031 | 0.00060 | Chr1:1688976 | N_Shelf | Body | | |  | | NADK | | II | | SLC35E2 (-11539), NADK (+22531) | | |  |
| cg16718760 | 0.832 | 0.861 | 0.029 | 0.00061 | Chr3:55721067 |  | Body | | |  | | ERC2 | | II | | WNT5A (-199737), ERC2 (+781323) | | |  |
| cg22373415 | 0.063 | 0.054 | -0.009 | 0.00061 | Chr15:66996361 | Island | Body;1stExon | | |  | | SMAD6 | | I | | SMAD3 (-361833), SMAD6 (+1688) | | |  |
| cg12151656 | 0.299 | 0.342 | 0.043 | 0.00062 | Chr3:9850558 | N_Shore | TSS1500 | | |  | | TTLL3 | | II | | TTLL3 (-1085) | | |  |
| cg10575369 | 0.845 | 0.873 | 0.028 | 0.00063 | Chr1:32217598 |  | Body | | | rs181150861 | | BAI2 | | II | | COL16A1 (-47831), BAI2 (+12049) | | |  |
| cg21049361 | 0.213 | 0.177 | -0.035 | 0.00063 | Chr6:30655870 | S_Shore | TSS1500;TSS200;3'UTR | | |  | | KIAA1949;NRM | | II | | PPP1R18 (-778) | | |  |
| cg08643692 | 0.081 | 0.061 | -0.020 | 0.00064 | Chr17:43099350 | Island |  | | |  | |  | | II | | C1QL1 (-53707), DCAKD (+29627) | | |  |
| cg16832267 | 0.057 | 0.063 | 0.006 | 0.00064 | Chr2:18060102 | Island | 5'UTR | | |  | | KCNS3 | | I | | KCNS3 (+158) | | |  |
| cg14245910 | 0.113 | 0.102 | -0.011 | 0.00066 | Chr7:129845377 | Island | TSS200 | | |  | | TMEM209 | | I | | C7orf45 (-2326), TMEM209 (-40) | | |  |
| cg26968498 | 0.865 | 0.837 | -0.028 | 0.00066 | Chr4:26174157 |  |  | | |  | |  | | II | | RBPJ (-148271), C4orf52 (+258344) | | |  |
| cg21131024 | 0.388 | 0.434 | 0.046 | 0.00067 | Chr8:104153643 | S_Shore | TSS200;Body | | |  | | C8orf56;BAALC | | I | | BAALC (+723) | | |  |
| cg01886396 | 0.104 | 0.085 | -0.018 | 0.00069 | Chr2:131099816 | Island | 5'UTR;TSS1500;1stExon | | |  | | CCDC115;IMP4;CCDC115 | | II | | IMP4 (-672), CCDC115 (+105) | | |  |
| cg14641472 | 0.150 | 0.200 | 0.049 | 0.00070 | Chr9:132387471 | N_Shore | TSS1500 | | |  | | METTL11A | | II | | METTL11A (-963) | | |  |
| cg05096751 | 0.229 | 0.171 | -0.057 | 0.00070 | Chr12:115137775 | Island |  | | |  | |  | | II | | TBX3 (-15807) | | |  |
| cg03585734 | 0.736 | 0.691 | -0.045 | 0.00071 | Chr1:15598865 |  | Body | | |  | | FHAD1 | | II | | EFHD2 (-137525), TMEM51 (+118637) | | |  |
| cg01722343 | 0.180 | 0.152 | -0.029 | 0.00071 | Chr4:25235437 | N_Shore | TSS1500 | | |  | | PI4K2B | | I | | PI4K2B (-215) | | |  |
| cg25325932 | 0.416 | 0.449 | 0.033 | 0.00071 | Chr6:76783470 |  | TSS1500 | | | rs140358468 | | IMPG1 | | II | | IMPG1 (-1136) | | |  |
| cg24638849 | 0.172 | 0.146 | -0.026 | 0.00072 | Chr6:84419360 | Island | TSS1500 | | |  | | SNAP91 | | II | | SNAP91 (-514) | | |  |
| cg03387930 | 0.158 | 0.142 | -0.017 | 0.00072 | Chr20:56725833 | N_Shore | TSS200 | | |  | | C20orf85 | | II | | PMEPA1 (-440803), PPP4R1L (+158661) | | |  |
| cg25580306 | 0.041 | 0.045 | 0.003 | 0.00072 | Chr22:42017000 | Island | TSS1500;TSS200 | | |  | | XRCC6;PPPDE2 | | I | | XRCC6 (-294) | | |  |
| cg26035892 | 0.750 | 0.700 | -0.050 | 0.00072 | Chr2:174855415 |  |  | | |  | |  | | II | | SP3 (-24986), OLA1 (+257949) | | |  |
| cg05184377 | 0.131 | 0.100 | -0.032 | 0.00073 | Chr10:50976646 | Island |  | | |  | |  | | I | | OGDHL (-6222), AGAP8 (+269888) | | |  |
| cg12286963 | 0.085 | 0.070 | -0.015 | 0.00073 | Chr11:72433438 |  | Body;TSS200 | | |  | | ARAP1 | | II | | PDE2A (-47942), ARAP1 (+29995) | | |  |
| cg20703240 | 0.029 | 0.032 | 0.003 | 0.00075 | Chr5:177659585 | Island | 5'UTR;1stExon | | |  | | AGXT2L2 | | I | | AGXT2L2 (+217) | | |  |
| cg19599202 | 0.046 | 0.055 | 0.009 | 0.00075 | Chr12:121124787 | Island | TSS200 | | |  | | MLEC | | I | | MLEC (-161) | | |  |
| cg20900312 | 0.734 | 0.681 | -0.054 | 0.00075 | Chr14:77670584 |  | 5'UTR | | |  | | TMEM63C | | II | | TMEM63C (+22483), NGB (+67070) | | |  |
| cg12937438 | 0.102 | 0.116 | 0.014 | 0.00075 | Chr1:13840411 | Island |  | | | rs79216547 | |  | | II | | LRRC38 (-170) | | |  |
| cg18131548 | 0.271 | 0.304 | 0.034 | 0.00076 | Chr1:47696212 | N_Shore | TSS1500 | | |  | | TAL1 | | II | | TAL1 (-770) | | |  |
| cg16536918 | 0.645 | 0.703 | 0.058 | 0.00076 | Chr20:3065403 | S_Shore | TSS200 | | |  | | AVP | | II | | AVP (-34) | | |  |
| cg14061069 | 0.284 | 0.255 | -0.029 | 0.00077 | Chr19:46274453 | Island | Body | | |  | | DMPK | | II | | SIX5 (-1957) | | |  |
| cg05798126 | 0.796 | 0.768 | -0.028 | 0.00077 | Chr6:3089496 | N_Shore | Body | | |  | | RIPK1 | | II | | BPHL (-29429), RIPK1 (+12499) | | |  |
| cg16463395 | 0.187 | 0.157 | -0.030 | 0.00077 | Chr3:93781653 | Island | TSS1500;1stExon;5'UTR | | |  | | NSUN3;DHFRL1 | | I | | NSUN3 (-201), DHFRL1 (+413) | | |  |
| cg13109528 | 0.792 | 0.839 | 0.048 | 0.00078 | Chr1:228477767 | S_Shelf | Body | | |  | | OBSCN | | II | | OBSCN (+81907), TRIM11 (+116749) | | |  |
| cg08566882 | 0.728 | 0.691 | -0.038 | 0.00078 | Chr11:131348843 |  | Body | | |  | | NTM | | II | | SNX19 (-562462), NTM (-431868) | | |  |
| cg24984312 | 0.441 | 0.475 | 0.034 | 0.00078 | Chr11:116707209 | S_Shore | Body | | |  | | APOA1 | | II | | APOA1 (+1128), APOC3 (+6586) | | |  |
| cg24924779 | 0.102 | 0.091 | -0.011 | 0.00079 | Chr20:49639998 | Island | TSS1500 | | |  | | KCNG1 | | I | | KCNG1 (-324) | | |  |
| cg21212505 | 0.635 | 0.593 | -0.042 | 0.00080 | Chr7:117228958 |  | Body | | |  | | CFTR | | II | | CFTR (+108942), CTTNBP2 (+284602) | | |  |
| cg07502892 | 0.755 | 0.791 | 0.036 | 0.00080 | Chr14:69274280 |  |  | | |  | |  | | II | | ZFP36L1 (-11321), ACTN1 (+171802) | | |  |
| cg09895354 | 0.706 | 0.764 | 0.058 | 0.00081 | Chr7:149476924 |  | Body | | |  | | SSPO | | II | | ZNF862 (-58584), SSPO (+3794) | | |  |
| cg18954434 | 0.052 | 0.062 | 0.010 | 0.00081 | Chr22:46692881 | Island | TSS1500;5'UTR | | |  | | CN5H6.4;GTSE1 | | I | | GTSE1 (+244) | | |  |
| cg16361013 | 0.857 | 0.897 | 0.040 | 0.00081 | Chr2:241513650 | S_Shore | Body | | |  | | RNPEPL1 | | I | | CAPN10 (-12482), RNPEPL1 (+5647) | | |  |
| cg14212891 | 0.878 | 0.835 | -0.044 | 0.00082 | Chr1:212733199 | S_Shore |  | | |  | |  | | II | | ATF3 (-48770), NENF (+126971) | | |  |
| cg02433671 | 0.174 | 0.245 | 0.071 | 0.00082 | Chr19:11529856 | Island | Body | | |  | | RGL3 | | I | | RGL3 (+161) | | |  |
| cg10236857 | 0.395 | 0.338 | -0.057 | 0.00085 | Chr17:79825681 | N_Shore | 3'UTR | | |  | | ARHGDIA | | I | | P4HB (-7138), ARHGDIA (+3600) | | |  |
| cg23683254 | 0.394 | 0.318 | -0.076 | 0.00085 | Chr14:99584667 | Island |  | | |  | |  | | II | | BCL11B (+153154) | | |  |
| cg16098340 | 0.624 | 0.662 | 0.038 | 0.00085 | Chr12:65014995 |  | TSS1500;Body | | |  | | MIR548C;RASSF3 | | II | | RASSF3 (+10703), GNS (+138230) | | |  |
| cg20197989 | 0.064 | 0.072 | 0.008 | 0.00085 | Chr17:260058 | Island | TSS200 | | |  | | C17orf97 | | I | | RPH3AL (-57426), VPS53 (+358037) | | |  |
| cg06124883 | 0.746 | 0.782 | 0.036 | 0.00086 | Chr1:15525193 |  | 5'UTR | | |  | | TMEM51 | | II | | EFHD2 (-211197), TMEM51 (+44965) | | |  |
| cg25931491 | 0.968 | 0.950 | -0.018 | 0.00086 | Chr11:1422468 |  | Body | | | rs117014395 | | BRSK2 | | I | | BRSK2 (+11340), MOB2 (+79646) | | |  |
| cg02891200 | 0.785 | 0.828 | 0.042 | 0.00086 | Chr12:109826483 |  | TSS200 | | |  | | MYO1H | | II | | MYO1H (-40) | | |  |
| cg04037707 | 0.532 | 0.482 | -0.050 | 0.00087 | Chr7:154704915 | N_Shore |  | | |  | |  | | II | | PAXIP1 (+89766), DPP6 (+955139) | | |  |
| cg11320084 | 0.250 | 0.208 | -0.042 | 0.00087 | Chr1:185014181 | N_Shore | TSS1500 | | |  | | RNF2 | | II | | RNF2 (-369) | | |  |
| cg18239686 | 0.885 | 0.866 | -0.019 | 0.00087 | Chr11:68748210 |  | 1stExon | | |  | | MRGPRD | | II | | MRGPRD (+244) | | |  |
| cg16595261 | 0.174 | 0.127 | -0.047 | 0.00088 | Chr2:242823679 | Island |  | | |  | |  | | I | | CXXC11 (+11794) | | |  |
| cg25040466 | 0.146 | 0.108 | -0.038 | 0.00089 | Chr20:61636172 | Island |  | | |  | |  | | II | | BHLHE23 (+2214), SLC17A9 (+52174) | | |  |
| cg06524192 | 0.840 | 0.864 | 0.025 | 0.00089 | Chr16:7615568 |  | Body | | |  | | A2BP1 | | II | | RBFOX1 (+232818) | | |  |
| cg08597292 | 0.765 | 0.809 | 0.044 | 0.00090 | Chr6:89996028 |  | Body | | |  | | GABRR2 | | II | | GABRR1 (-68533), GABRR2 (+28938) | | |  |
| cg19820328 | 0.572 | 0.610 | 0.038 | 0.00091 | Chr10:125332630 |  |  | | |  | |  | | II | | GPR26 (-93240), BUB3 (+418871) | | |  |
| cg07572052 | 0.178 | 0.208 | 0.030 | 0.00091 | Chr1:149821168 | N_Shore | TSS1500 | | |  | | HIST2H2AA4;HIST2H2AA3 | | II | | HIST2H3C (-3012), HIST2H2AA3 (-1459), HIST2H2BC (+1171) | | |  |
| cg20151107 | 0.867 | 0.839 | -0.028 | 0.00092 | Chr10:126331425 |  | Body | | |  | | FAM53B | | II | | FAM53B (+101504), LHPP (+181085) | | |  |
| cg12064947 | 0.057 | 0.070 | 0.013 | 0.00092 | Chr15:41220983 | Island | TSS1500 | | |  | | DLL4 | | II | | DLL4 (-547) | | |  |
| cg17800654 | 0.450 | 0.543 | 0.093 | 0.00092 | Chr1:50888590 | Island | 5'UTR | | |  | | DMRTA2 | | I | | DMRTA2 (+528) | | |  |
| cg20416280 | 0.840 | 0.795 | -0.045 | 0.00093 | Chr17:11103904 |  |  | | | rs146920803 | |  | | II | | PIRT (-362487), SHISA6 (-40835) | | |  |
| cg12220663 | 0.717 | 0.677 | -0.041 | 0.00095 | Chr2:20194085 | S_Shelf | Body | | |  | | MATN3 | | II | | WDR35 (-4202) | | |  |
| cg26038547 | 0.817 | 0.846 | 0.029 | 0.00095 | Chr12:52398421 | N_Shelf |  | | |  | |  | | II | | GRASP (-2326) | | |  |
| cg01027209 | 0.886 | 0.910 | 0.024 | 0.00095 | Chr13:107029443 |  |  | | |  | |  | | II | | EFNB2 (+157944), DAOA (+910879) | | |  |
| cg24157814 | 0.809 | 0.782 | -0.027 | 0.00096 | Chr13:95116571 |  | Body | | |  | | DCT;DCT | | II | | DCT (+15364) | | |  |
| cg26543799 | 0.114 | 0.099 | -0.015 | 0.00097 | Chr2:203736570 | Island | TSS200;TSS1500 | | |  | | ICA1L;ICA1L | | I | | WDR12 (+40378), BMPR2 (+495521) | | |  |
| cg11466131 | 0.788 | 0.756 | -0.032 | 0.00097 | Chr22:37954778 | N_Shore |  | | |  | |  | | II | | CDC42EP1 (-1692) | | |  |
| cg09518518 | 0.814 | 0.833 | 0.019 | 0.00097 | Chr1:12703968 |  | TSS1500 | | |  | | AADACL4 | | II | | AADACL4 (-597) | | |  |
| cg03331715 | 0.847 | 0.815 | -0.033 | 0.00097 | Chr12:104689087 |  | Body;5'UTR | | |  | | TXNRD1 | | II | | EID3 (-8422), TXNRD1 (+79529) | | |  |
| cg03596016 | 0.174 | 0.140 | -0.034 | 0.00098 | Chr1:246952362 | Island | TSS1500 | | |  | | LOC149134 | | I | | SCCPDH (+64985), AHCTF1 (+142363) | | |  |
| cg05329976 | 0.734 | 0.774 | 0.040 | 0.00098 | Chr20:58295160 |  | TSS1500;Body | | |  | | PHACTR3 | | II | | PHACTR3 (+115558), SYCP2 (+212048) | | |  |
| cg06330055 | 0.881 | 0.862 | -0.018 | 0.00098 | Chr11:129564162 |  |  | | |  | |  | | I | | TMEM45B (-121578), BARX2 (+318282) | | |  |
| cg21809913 | 0.750 | 0.782 | 0.032 | 0.00099 | Chr14:101500052 |  | TSS200 | | |  | | MIR495 | | II | | DIO3 (-527635), RTL1 (-148869) | | |  |
| cg22234194 | 0.064 | 0.059 | -0.005 | 0.00099 | Chr11:45868879 | Island | Body;TSS200 | | |  | | CRY2 | | I | | CRY2 (-77) | | |  |
| cg05716075 | 0.834 | 0.802 | -0.032 | 0.00099 | Chr16:88476249 |  |  | | |  | |  | | II | | ZNF469 (-17629), BANP (+491212) | | |  |
| cg19376709 | 0.068 | 0.061 | -0.008 | 0.00100 | Chr1:149858318 | Island | TSS1500;TSS200 | | |  | | HIST2H2AC;HIST2H2BE | | I | | HIST2H2AC (-206), HIST2H2BE (-87) | | |  |
| Ranked by P value. DMPs, differentially methylated positions; MZ, monozygotic; GREAT, Genomic Regions Enrichment of Annotations Tool; TSS, transcription start site. SBE: Single base extension. ∆β: Change in DNA methylation (Affected twin β – Unaffected twin β). UCSC: University of California Santa Cruz genome browser. SNP: Single nucleotide polymorphism. | | | | | | | | | | | | | | | | | |  |  |

| **Supplementary Table S2. Gene Ontology enrichment analysis for age-10 asthma-associated DMPs** | |  |  |
| --- | --- | --- | --- |
| **GO Accession ID** | **GO Function** | **Ontology** | **P value** |
| GO:0000786 | nucleosome | CC | 0.00020 |
| GO:1990104 | DNA bending complex | CC | 0.00020 |
| GO:0044815 | DNA packaging complex | CC | 0.00034 |
| GO:0043269 | regulation of ion transport | BP | 0.00080 |
| GO:0042592 | homeostatic process | BP | 0.00134 |
| GO:0070069 | cytochrome complex | CC | 0.00141 |
| GO:0048821 | erythrocyte development | BP | 0.00145 |
| GO:0019725 | cellular homeostasis | BP | 0.00237 |
| GO:0044070 | regulation of anion transport | BP | 0.00258 |
| GO:0006695 | cholesterol biosynthetic process | BP | 0.00280 |
| GO:0003084 | positive regulation of systemic arterial blood pressure | BP | 0.00284 |
| GO:0006811 | ion transport | BP | 0.00313 |
| GO:1902495 | transmembrane transporter complex | CC | 0.00333 |
| GO:1990351 | transporter complex | CC | 0.00357 |
| GO:0035024 | negative regulation of Rho protein signal transduction | BP | 0.00383 |
| GO:0030018 | Z disc | CC | 0.00404 |
| GO:0061178 | regulation of insulin secretion involved in cellular response to glucose stimulus | BP | 0.00407 |
| GO:0007266 | Rho protein signal transduction | BP | 0.00458 |
| GO:0007369 | gastrulation | BP | 0.00460 |
| GO:0002562 | somatic diversification of immune receptors via germline recombination within a single locus | BP | 0.00483 |
| GO:0016444 | somatic cell DNA recombination | BP | 0.00483 |
| GO:0005251 | delayed rectifier potassium channel activity | MF | 0.00490 |
| GO:0055082 | cellular chemical homeostasis | BP | 0.00499 |
| GO:0034483 | heparan sulfate sulfotransferase activity | MF | 0.00523 |
| GO:0002200 | somatic diversification of immune receptors | BP | 0.00533 |
| GO:0035773 | insulin secretion involved in cellular response to glucose stimulus | BP | 0.00538 |
| GO:0045191 | regulation of isotype switching | BP | 0.00558 |
| GO:0031674 | I band | CC | 0.00585 |
| GO:0016126 | sterol biosynthetic process | BP | 0.00587 |
| GO:0022836 | gated channel activity | MF | 0.00641 |
| GO:0034765 | regulation of ion transmembrane transport | BP | 0.00673 |
| GO:0002440 | production of molecular mediator of immune response | BP | 0.00734 |
| GO:0090049 | regulation of cell migration involved in sprouting angiogenesis | BP | 0.00742 |
| GO:0043566 | structure-specific DNA binding | MF | 0.00785 |
| GO:0034762 | regulation of transmembrane transport | BP | 0.00799 |
| GO:0019229 | regulation of vasoconstriction | BP | 0.00864 |
| GO:0044665 | MLL1/2 complex | CC | 0.00940 |
| GO:0071339 | MLL1 complex | CC | 0.00940 |
| GO:0006972 | hyperosmotic response | BP | 0.00946 |
| GO:0060402 | calcium ion transport into cytosol | BP | 0.00962 |
| GO:2000179 | positive regulation of neural precursor cell proliferation | BP | 0.00971 |
| GO:0060401 | cytosolic calcium ion transport | BP | 0.00987 |
| GO:0046982 | protein heterodimerization activity | MF | 0.01037 |
| GO:0032993 | protein-DNA complex | CC | 0.01056 |
| GO:0002377 | immunoglobulin production | BP | 0.01072 |
| GO:0002700 | regulation of production of molecular mediator of immune response | BP | 0.01090 |
| GO:0030003 | cellular cation homeostasis | BP | 0.01093 |
| GO:1990204 | oxidoreductase complex | CC | 0.01131 |
| GO:0009798 | axis specification | BP | 0.01184 |
| GO:0034220 | ion transmembrane transport | BP | 0.01205 |
| GO:0061515 | myeloid cell development | BP | 0.01209 |
| GO:0021781 | glial cell fate commitment | BP | 0.01218 |
| GO:0035023 | regulation of Rho protein signal transduction | BP | 0.01252 |
| GO:0043537 | negative regulation of blood vessel endothelial cell migration | BP | 0.01267 |
| GO:0032489 | regulation of Cdc42 protein signal transduction | BP | 0.01279 |
| GO:0002637 | regulation of immunoglobulin production | BP | 0.01306 |
| GO:0002028 | regulation of sodium ion transport | BP | 0.01316 |
| GO:0006873 | cellular ion homeostasis | BP | 0.01320 |
| GO:0016525 | negative regulation of angiogenesis | BP | 0.01345 |
| GO:0005216 | ion channel activity | MF | 0.01392 |
| GO:0008203 | cholesterol metabolic process | BP | 0.01416 |
| GO:0009651 | response to salt stress | BP | 0.01437 |
| GO:0022838 | substrate-specific channel activity | MF | 0.01493 |
| GO:0015012 | heparan sulfate proteoglycan biosynthetic process | BP | 0.01503 |
| GO:0050880 | regulation of blood vessel size | BP | 0.01538 |
| GO:0033500 | carbohydrate homeostasis | BP | 0.01554 |
| GO:0042593 | glucose homeostasis | BP | 0.01554 |
| GO:0007588 | excretion | BP | 0.01556 |
| GO:0035150 | regulation of tube size | BP | 0.01570 |
| GO:0000791 | euchromatin | CC | 0.01616 |
| GO:0045907 | positive regulation of vasoconstriction | BP | 0.01674 |
| GO:0002052 | positive regulation of neuroblast proliferation | BP | 0.01684 |
| GO:0006812 | cation transport | BP | 0.01703 |
| GO:0030017 | sarcomere | CC | 0.01712 |
| GO:1990391 | DNA repair complex | CC | 0.01750 |
| GO:0051453 | regulation of intracellular pH | BP | 0.01790 |
| GO:0098660 | inorganic ion transmembrane transport | BP | 0.01807 |
| GO:0042310 | vasoconstriction | BP | 0.01818 |
| GO:0015267 | channel activity | MF | 0.01881 |
| GO:0022803 | passive transmembrane transporter activity | MF | 0.01881 |
| GO:0007204 | positive regulation of cytosolic calcium ion concentration | BP | 0.01898 |
| GO:0002712 | regulation of B cell mediated immunity | BP | 0.01900 |
| GO:0002889 | regulation of immunoglobulin mediated immune response | BP | 0.01900 |
| GO:0043534 | blood vessel endothelial cell migration | BP | 0.01944 |
| GO:0015672 | monovalent inorganic cation transport | BP | 0.01963 |
| GO:0098655 | cation transmembrane transport | BP | 0.01986 |
| GO:0051781 | positive regulation of cell division | BP | 0.02027 |
| GO:0044212 | transcription regulatory region DNA binding | MF | 0.02060 |
| GO:0048878 | chemical homeostasis | BP | 0.02066 |
| GO:0050801 | ion homeostasis | BP | 0.02087 |
| GO:0030641 | regulation of cellular pH | BP | 0.02091 |
| GO:0002204 | somatic recombination of immunoglobulin genes involved in immune response | BP | 0.02092 |
| GO:0002208 | somatic diversification of immunoglobulins involved in immune response | BP | 0.02092 |
| GO:0045190 | isotype switching | BP | 0.02092 |
| GO:0016125 | sterol metabolic process | BP | 0.02130 |
| GO:0005996 | monosaccharide metabolic process | BP | 0.02175 |
| GO:0006091 | generation of precursor metabolites and energy | BP | 0.02178 |
| GO:0045777 | positive regulation of blood pressure | BP | 0.02180 |
| GO:0006874 | cellular calcium ion homeostasis | BP | 0.02228 |
| GO:0055085 | transmembrane transport | BP | 0.02255 |
| GO:0000975 | regulatory region DNA binding | MF | 0.02257 |
| GO:0001067 | regulatory region nucleic acid binding | MF | 0.02257 |
| GO:0003018 | vascular process in circulatory system | BP | 0.02309 |
| GO:0044449 | contractile fiber part | CC | 0.02323 |
| GO:0050796 | regulation of insulin secretion | BP | 0.02340 |
| GO:0003014 | renal system process | BP | 0.02412 |
| GO:0006875 | cellular metal ion homeostasis | BP | 0.02412 |
| GO:0001707 | mesoderm formation | BP | 0.02424 |
| GO:0030201 | heparan sulfate proteoglycan metabolic process | BP | 0.02424 |
| GO:0019866 | organelle inner membrane | CC | 0.02463 |
| GO:0009950 | dorsal/ventral axis specification | BP | 0.02483 |
| GO:0071333 | cellular response to glucose stimulus | BP | 0.02491 |
| GO:0032412 | regulation of ion transmembrane transporter activity | BP | 0.02559 |
| GO:0005520 | insulin-like growth factor binding | MF | 0.02588 |
| GO:0009719 | response to endogenous stimulus | BP | 0.02629 |
| GO:0010596 | negative regulation of endothelial cell migration | BP | 0.02638 |
| GO:0055080 | cation homeostasis | BP | 0.02655 |
| GO:0055074 | calcium ion homeostasis | BP | 0.02656 |
| GO:0002042 | cell migration involved in sprouting angiogenesis | BP | 0.02660 |
| GO:0071331 | cellular response to hexose stimulus | BP | 0.02680 |
| GO:0006006 | glucose metabolic process | BP | 0.02690 |
| GO:0060191 | regulation of lipase activity | BP | 0.02731 |
| GO:0071326 | cellular response to monosaccharide stimulus | BP | 0.02752 |
| GO:0072503 | cellular divalent inorganic cation homeostasis | BP | 0.02763 |
| GO:0005547 | phosphatidylinositol-3,4,5-trisphosphate binding | MF | 0.02785 |
| GO:0048332 | mesoderm morphogenesis | BP | 0.02790 |
| GO:0051480 | cytosolic calcium ion homeostasis | BP | 0.02791 |
| GO:0034702 | ion channel complex | CC | 0.02799 |
| GO:0032228 | regulation of synaptic transmission, GABAergic | BP | 0.02842 |
| GO:2000648 | positive regulation of stem cell proliferation | BP | 0.02855 |
| GO:0005720 | nuclear heterochromatin | CC | 0.02856 |
| GO:0022898 | regulation of transmembrane transporter activity | BP | 0.02891 |
| GO:0005637 | nuclear inner membrane | CC | 0.02905 |
| GO:0016447 | somatic recombination of immunoglobulin gene segments | BP | 0.02958 |
| GO:0008076 | voltage-gated potassium channel complex | CC | 0.02978 |
| GO:0030016 | myofibril | CC | 0.03016 |
| GO:0031965 | nuclear membrane | CC | 0.03018 |
| GO:0046456 | icosanoid biosynthetic process | BP | 0.03022 |
| GO:1901570 | fatty acid derivative biosynthetic process | BP | 0.03022 |
| GO:0090276 | regulation of peptide hormone secretion | BP | 0.03053 |
| GO:0034705 | potassium channel complex | CC | 0.03149 |
| GO:0032844 | regulation of homeostatic process | BP | 0.03208 |
| GO:0016445 | somatic diversification of immunoglobulins | BP | 0.03212 |
| GO:0051932 | synaptic transmission, GABAergic | BP | 0.03225 |
| GO:0002791 | regulation of peptide secretion | BP | 0.03253 |
| GO:0002381 | immunoglobulin production involved in immunoglobulin mediated immune response | BP | 0.03259 |
| GO:0008217 | regulation of blood pressure | BP | 0.03270 |
| GO:0000018 | regulation of DNA recombination | BP | 0.03295 |
| GO:1902692 | regulation of neuroblast proliferation | BP | 0.03312 |
| GO:0090087 | regulation of peptide transport | BP | 0.03334 |
| GO:0001702 | gastrulation with mouth forming second | BP | 0.03427 |
| GO:2000177 | regulation of neural precursor cell proliferation | BP | 0.03427 |
| GO:0015075 | ion transmembrane transporter activity | MF | 0.03442 |
| GO:0051056 | regulation of small GTPase mediated signal transduction | BP | 0.03470 |
| GO:0043292 | contractile fiber | CC | 0.03491 |
| GO:0044427 | chromosomal part | CC | 0.03528 |
| GO:0072507 | divalent inorganic cation homeostasis | BP | 0.03545 |
| GO:0032409 | regulation of transporter activity | BP | 0.03600 |
| GO:0051260 | protein homooligomerization | BP | 0.03615 |
| GO:0030073 | insulin secretion | BP | 0.03625 |
| GO:0002312 | B cell activation involved in immune response | BP | 0.03664 |
| GO:0072089 | stem cell proliferation | BP | 0.03685 |
| GO:0001678 | cellular glucose homeostasis | BP | 0.03693 |
| GO:0005261 | cation channel activity | MF | 0.03758 |
| GO:0033209 | tumor necrosis factor-mediated signaling pathway | BP | 0.03772 |
| GO:0048333 | mesodermal cell differentiation | BP | 0.03777 |
| GO:0043627 | response to estrogen | BP | 0.03849 |
| GO:0008015 | blood circulation | BP | 0.03912 |
| GO:0033017 | sarcoplasmic reticulum membrane | CC | 0.03920 |
| GO:0032414 | positive regulation of ion transmembrane transporter activity | BP | 0.03975 |
| GO:0031663 | lipopolysaccharide-mediated signaling pathway | BP | 0.04006 |
| GO:0003013 | circulatory system process | BP | 0.04011 |
| GO:0070528 | protein kinase C signaling | BP | 0.04219 |
| GO:0010720 | positive regulation of cell development | BP | 0.04227 |
| GO:0005249 | voltage-gated potassium channel activity | MF | 0.04302 |
| GO:0006942 | regulation of striated muscle contraction | BP | 0.04303 |
| GO:0071889 | 14-3-3 protein binding | MF | 0.04317 |
| GO:0050661 | NADP binding | MF | 0.04377 |
| GO:0071322 | cellular response to carbohydrate stimulus | BP | 0.04462 |
| GO:0098662 | inorganic cation transmembrane transport | BP | 0.04472 |
| GO:0001708 | cell fate specification | BP | 0.04472 |
| GO:0006636 | unsaturated fatty acid biosynthetic process | BP | 0.04491 |
| GO:0019318 | hexose metabolic process | BP | 0.04535 |
| GO:0002718 | regulation of cytokine production involved in immune response | BP | 0.04581 |
| GO:0006310 | DNA recombination | BP | 0.04602 |
| GO:0055114 | oxidation-reduction process | BP | 0.04613 |
| GO:0055065 | metal ion homeostasis | BP | 0.04671 |
| GO:0008324 | cation transmembrane transporter activity | MF | 0.04682 |
| GO:0046578 | regulation of Ras protein signal transduction | BP | 0.04700 |
| GO:0044455 | mitochondrial membrane part | CC | 0.04717 |
| GO:0018298 | protein-chromophore linkage | BP | 0.04736 |
| GO:0034707 | chloride channel complex | CC | 0.04779 |
| GO:0030004 | cellular monovalent inorganic cation homeostasis | BP | 0.04791 |
| GO:0034703 | cation channel complex | CC | 0.04792 |
| GO:0034612 | response to tumor necrosis factor | BP | 0.04796 |
| GO:0005100 | Rho GTPase activator activity | MF | 0.04898 |
| GO:0008146 | sulfotransferase activity | MF | 0.04915 |
| GO:0050769 | positive regulation of neurogenesis | BP | 0.04930 |
| GO:0005746 | mitochondrial respiratory chain | CC | 0.04999 |
| Ranked by P value. GO: Gene Ontology, DMPs: Differentially Methylated Positions; BP: Biological process; | | |  |
| MF: Molecular Function; CC: Cellular Component | |  |  |

| **Supplementary Table S3. The top-ranked DMPs at age 10 in persistent asthma discordant MZ twins (between 10-18 years)** | | | | | | | |  |  | | |  |  | | |  |  | | |
| --- | --- | --- | --- | --- | --- | --- | --- | --- | --- | --- | --- | --- | --- | --- | --- | --- | --- | --- | --- |
| **Probe ID** | **Unaffected Co-Twin Mean** | **Asthma**  **Twin Mean** | **Mean ∆β** | **P value** | **Hg19** | **Relation to CpG Island** | **Gene region feature category (UCSC)** | | | **SNPs in probe (+/- 10bp SBE)** | **Illumina Gene Annotation** | | | **Probe Type** | **Gene Annotation from GREAT (Distance from TSS)** | | | |  |
| cg23603194 | 0.289 | 0.180 | -0.109 | 6.87E-06 | Chr1:221060753 | N_Shore |  | | |  |  | | | II | HLX (+8011), DUSP10 (+854762) | | | |  |
| cg27385757 | 0.353 | 0.286 | -0.067 | 2.16E-05 | Chr6:30656024 | S_Shore | TSS1500;3'UTR | | |  | KIAA1949;NRM | | | II | PPP1R18 (-932) | | | |  |
| cg06483698 | 0.805 | 0.848 | 0.043 | 2.64E-05 | Chr7:6310290 | N_Shore | Body | | |  | CYTH3 | | | II | CYTH3 (+1951), USP42 (+165741) | | | |  |
| cg05895618 | 0.559 | 0.655 | 0.096 | 2.69E-05 | Chr11:19222395 |  | 5'UTR | | |  | CSRP3 | | | II | CSRP3 (+1193), ZDHHC13 (+83704) | | | |  |
| cg14868530 | 0.081 | 0.098 | 0.017 | 3.36E-05 | Chr15:73976679 | Island | 5'UTR;1stExon | | |  | CD276 | | | I | CD276 (+58) | | | |  |
| cg21304454 | 0.530 | 0.392 | -0.138 | 3.59E-05 | Chr1:17302859 | N_Shelf | Body | | |  | MFAP2 | | | II | MFAP2 (+4313), CROCC (+54415) | | | |  |
| cg11998205 | 0.853 | 0.806 | -0.047 | 4.28E-05 | Chr19:48840527 | S_Shelf | Body | | |  | TMEM143 | | | I | EMP3 (+11899), TMEM143 (+26658) | | | |  |
| cg18513344 | 0.210 | 0.137 | -0.072 | 4.39E-05 | Chr3:195531298 |  | Body | | |  | MUC4 | | | II | MUC4 (+7545), MUC20 (+83546) | | | |  |
| cg08657206 | 0.256 | 0.221 | -0.035 | 5.55E-05 | Chr1:180197901 | N_Shore |  | | | rs7530324 |  | | | I | LHX4 (-1531) | | | |  |
| cg19865472 | 0.897 | 0.844 | -0.052 | 5.90E-05 | Chr19:617133 | Island | 3'UTR | | |  | HCN2 | | | I | POLRMT (+16434), HCN2 (+27241) | | | |  |
| cg26280666 | 0.098 | 0.072 | -0.026 | 6.13E-05 | Chr8:72755568 | N_Shore | Body | | |  | MSC | | | II | EYA1 (-486590), MSC (+1162) | | | |  |
| cg13512987 | 0.525 | 0.645 | 0.120 | 6.77E-05 | Chr12:6898785 |  | 5'UTR;1stExon | | |  | CD4 | | | II | CD4 (+148) | | | |  |
| cg24664347 | 0.736 | 0.816 | 0.080 | 7.51E-05 | Chr8:86841010 | S_Shore | TSS1500 | | |  | REXO1L2P | | | II | REXO1L1 (-51705), PSKH2 (+240840) | | | |  |
| cg25346117 | 0.669 | 0.745 | 0.076 | 8.06E-05 | Chr1:116923256 |  | Body | | |  | ATP1A1 | | | II | ATP1A1 (+7462), CD58 (+190458) | | | |  |
| cg09587549 | 0.232 | 0.265 | 0.033 | 9.75E-05 | Chr16:31214126 | Island | 5'UTR;1stExon | | |  | PYCARD | | | I | PYCARD (-30) | | | |  |
| cg03225548 | 0.765 | 0.681 | -0.084 | 9.90E-05 | Chr18:71811234 | N_Shelf | Body;5'UTR | | |  | FBXO15 | | | II | TIMM21 (-4511), FBXO15 (+3865) | | | |  |
| cg17130754 | 0.756 | 0.697 | -0.060 | 9.99E-05 | Chr10:3536341 |  |  | | |  |  | | | II | PITRM1 (-321309), KLF6 (+291131) | | | |  |
| cg00307818 | 0.882 | 0.911 | 0.029 | 0.000107 | Chr1:55463592 | S_Shore | TSS1500 | | |  | BSND | | | II | BSND (-1024) | | | |  |
| cg26078513 | 0.208 | 0.131 | -0.078 | 0.000109 | Chr17:27347260 | S_Shore |  | | |  |  | | | II | PIPOX (-22657), SEZ6 (-14180) | | | |  |
| cg08645720 | 0.104 | 0.125 | 0.021 | 0.000112 | Chr6:80247344 | Island | TSS200 | | |  | LCA5 | | | I | LCA5 (-198) | | | |  |
| cg08651763 | 0.055 | 0.064 | 0.009 | 0.000113 | Chr10:79686680 | S_Shore | Body;TSS1500 | | |  | LOC100128292;DLG5 | | | I | DLG5 (-333) | | | |  |
| cg19943238 | 0.755 | 0.683 | -0.072 | 0.000118 | Chr11:2018095 | Island | Body;TSS200 | | |  | H19;MIR675 | | | I | MRPL23 (+49594), IGF2 (+144245) | | | |  |
| cg03591753 | 0.511 | 0.568 | 0.057 | 0.000119 | Chr6:35659141 | S_Shelf | 5'UTR | | |  | FKBP5 | | | II | FKBP5 (-2423) | | | |  |
| cg20298273 | 0.100 | 0.116 | 0.015 | 0.000121 | Chr1:156897330 | Island | Body | | |  | C1orf92 | | | I | PEAR1 (+33808), ARHGEF11 (+117831) | | | |  |
| cg16609342 | 0.601 | 0.538 | -0.063 | 0.000123 | Chr22:48973245 | S_Shore | Body | | |  | FAM19A5 | | | II | FAM19A5 (+87958) | | | |  |
| cg14862981 | 0.181 | 0.145 | -0.036 | 0.000124 | Chr5:180617100 | Island |  | | |  |  | | | I | TRIM7 (+15076), OR2V2 (+35158) | | | |  |
| cg04611717 | 0.650 | 0.740 | 0.090 | 0.000125 | Chr12:132897840 | S_Shore | Body | | |  | GALNT9 | | | II | GALNT9 (+8064), NOC4L (+268848) | | | |  |
| cg22876561 | 0.099 | 0.121 | 0.022 | 0.000130 | Chr7:11013721 | Island | 1stExon;5'UTR | | |  | PHF14 | | | I | PHF14 (+223) | | | |  |
| cg26275360 | 0.787 | 0.740 | -0.047 | 0.000135 | Chr8:52852168 |  |  | | |  |  | | | II | PCMTD1 (-40434), ST18 (+470270) | | | |  |
| cg14592673 | 0.324 | 0.273 | -0.052 | 0.000142 | Chr17:48768755 |  | 3'UTR | | |  | ABCC3 | | | II | LUC7L3 (-28170), ABCC3 (+56538) | | | |  |
| cg01780466 | 0.048 | 0.059 | 0.011 | 0.000143 | Chr1:145477253 | Island | 1stExon | | | rs2282013 | LIX1L | | | I | LIX1L (+169) | | | |  |
| cg18109665 | 0.475 | 0.382 | -0.093 | 0.000147 | Chr3:9062474 |  | Body | | |  | SRGAP3 | | | II | RAD18 (-57316), SRGAP3 (+228836) | | | |  |
| cg14153876 | 0.206 | 0.184 | -0.022 | 0.000147 | Chr2:157292773 | S_Shore | 5'UTR;TSS200 | | |  | GPD2;GPD2 | | | I | GPD2 (-126) | | | |  |
| cg16832267 | 0.054 | 0.064 | 0.010 | 0.000151 | Chr2:18060102 | Island | 5'UTR | | |  | KCNS3 | | | I | KCNS3 (+158) | | | |  |
| cg23979876 | 0.553 | 0.479 | -0.074 | 0.000154 | Chr4:141299001 | S_Shelf | Body | | |  | SCOC | | | II | SCOC (+34405), CLGN (+49813) | | | |  |
| cg15090119 | 0.850 | 0.774 | -0.076 | 0.000162 | Chr17:80882894 | N_Shelf | Body | | |  | TBCD | | | I | ZNF750 (-84964), B3GNTL1 (+126791) | | | |  |
| cg03446244 | 0.354 | 0.280 | -0.075 | 0.000174 | Chr16:86229872 |  |  | | |  |  | | | I | FOXF1 (-314260), IRF8 (+297099) | | | |  |
| cg24813995 | 0.389 | 0.341 | -0.048 | 0.000181 | Chr6:30657882 | S_Shelf | Body | | |  | NRM | | | II | PPP1R18 (-2790) | | | |  |
| cg09875624 | 0.799 | 0.737 | -0.062 | 0.000182 | Chr11:3254504 | S_Shore | TSS1500 | | |  | MRGPRE | | | II | MRGPRE (-889) | | | |  |
| cg23758309 | 0.727 | 0.810 | 0.083 | 0.000183 | Chr12:97273300 |  |  | | |  |  | | | II | CDK17 (-478935), NEDD1 (-27700) | | | |  |
| cg03043709 | 0.966 | 0.957 | -0.008 | 0.000191 | Chr7:2046920 | S_Shelf | Body | | |  | MAD1L1 | | | I | MAD1L1 (+225662), ELFN1 (+298123) | | | |  |
| cg12570419 | 0.843 | 0.738 | -0.106 | 0.000200 | Chr9:138636968 | Island | Body | | |  | KCNT1 | | | II | KCNT1 (+42938), CAMSAP1 (+162036) | | | |  |
| cg26918471 | 0.132 | 0.114 | -0.018 | 0.000204 | Chr5:121297617 | Island | TSS200 | | |  | SRFBP1 | | | I | SRFBP1 (-38) | | | |  |
| cg03966010 | 0.874 | 0.905 | 0.031 | 0.000207 | Chr7:157402413 | N_Shelf | Body | | |  | PTPRN2 | | | II | DNAJB6 (+272704), PTPRN2 (+978068) | | | |  |
| cg04185123 | 0.814 | 0.762 | -0.051 | 0.000209 | Chr5:146812118 |  | Body | | |  | DPYSL3 | | | II | DPYSL3 (+77500), STK32A (+197540) | | | |  |
| cg21573796 | 0.836 | 0.806 | -0.030 | 0.000214 | Chr15:44394647 |  | Body | | |  | FRMD5 | | | II | MFAP1 (-277697), FRMD5 (+92781) | | | |  |
| cg15553814 | 0.765 | 0.837 | 0.072 | 0.000228 | Chr1:58450472 |  | 5'UTR | | |  | DAB1 | | | II | DAB1 (+265738) | | | |  |
| cg09820180 | 0.575 | 0.482 | -0.092 | 0.000234 | Chr6:140168822 |  |  | | |  |  | | | II | CITED2 (-473473) | | | |  |
| cg23654112 | 0.303 | 0.263 | -0.039 | 0.000235 | Chr16:2525928 | Island | 5'UTR | | |  | TBC1D24 | | | II | TBC1D24 (+782) | | | |  |
| cg26262152 | 0.291 | 0.229 | -0.062 | 0.000235 | Chr4:493729 | S_Shore | TSS1500;Body | | |  | ZNF721;PIGG | | | II | ZNF721 (-288), PIGG (+741) | | | |  |
| cg08489349 | 0.222 | 0.144 | -0.079 | 0.000238 | Chr17:656181 | Island | Body;TSS1500 | | |  | ELP2P;GEMIN4 | | | II | GEMIN4 (-681) | | | |  |
| cg16718760 | 0.809 | 0.858 | 0.049 | 0.000241 | Chr3:55721067 |  | Body | | |  | ERC2 | | | II | WNT5A (-199737), ERC2 (+781323) | | | |  |
| cg04999902 | 0.721 | 0.796 | 0.075 | 0.000249 | Chr17:47577854 | S_Shelf | Body | | |  | NGFR | | | II | NXPH3 (-75443), NGFR (+5200) | | | |  |
| cg13325066 | 0.771 | 0.706 | -0.065 | 0.000250 | Chr7:2499932 | Island |  | | |  |  | | | II | LFNG (-59546), CHST12 (+56738) | | | |  |
| cg08884928 | 0.703 | 0.622 | -0.081 | 0.000255 | Chr17:57431996 |  | Body | | |  | YPEL2 | | | II | DHX40 (-210889), YPEL2 (+22944) | | | |  |
| cg19151121 | 0.170 | 0.213 | 0.043 | 0.000262 | Chr11:133402184 |  | 1stExon | | |  | OPCML | | | I | OPCML (-589148), SPATA19 (+313207) | | | |  |
| cg12414174 | 0.406 | 0.340 | -0.066 | 0.000268 | Chr12:89748895 | S_Shore |  | | |  |  | | | II | DUSP6 (-2600) | | | |  |
| cg09949789 | 0.493 | 0.413 | -0.080 | 0.000268 | Chr4:6990245 | S_Shore | Body | | |  | TBC1D14 | | | II | TADA2B (-54910), TBC1D14 (+78751) | | | |  |
| cg11668844 | 0.110 | 0.168 | 0.058 | 0.000272 | Chr13:113655622 | N_Shore | Body | | |  | MCF2L | | | II | F7 (-104482), MCF2L (+32088) | | | |  |
| cg00369202 | 0.370 | 0.299 | -0.071 | 0.000278 | Chr6:33989844 |  | 3'UTR | | |  | GRM4 | | | II | MLN (-218052), GRM4 (+111791) | | | |  |
| cg02337355 | 0.285 | 0.360 | 0.076 | 0.000278 | Chr4:493782 | S_Shore | TSS1500;Body | | |  | ZNF721;PIGG | | | II | ZNF721 (-341), PIGG (+794) | | | |  |
| cg13307718 | 0.754 | 0.813 | 0.059 | 0.000281 | Chr11:85995223 |  |  | | |  |  | | | II | C11orf73 (-18029), EED (+39418) | | | |  |
| cg08970649 | 0.408 | 0.322 | -0.086 | 0.000283 | Chr8:18905201 |  |  | | |  |  | | | II | SH2D4A (-266285), PSD3 (-34006) | | | |  |
| cg08820507 | 0.692 | 0.739 | 0.047 | 0.000285 | Chr7:5278117 |  |  | | |  |  | | | II | SLC29A4 (-44443), WIPI2 (+48283) | | | |  |
| cg26725076 | 0.383 | 0.346 | -0.037 | 0.000294 | Chr12:132569963 | S_Shore | Body | | |  | EP400NL | | | II | DDX51 (+58916), EP400 (+135499) | | | |  |
| cg16098955 | 0.365 | 0.270 | -0.095 | 0.000294 | Chr2:55172992 |  | Body | | |  | EML6 | | | II | RTN4 (+104741), EML6 (+220844) | | | |  |
| cg14780276 | 0.524 | 0.441 | -0.082 | 0.000296 | Chr4:148743200 |  | Body | | |  | ARHGAP10 | | | II | ARHGAP10 (+89748), NR3C2 (+620471) | | | |  |
| cg08224888 | 0.397 | 0.312 | -0.085 | 0.000307 | Chr19:3969522 | N_Shore | Body | | |  | DAPK3 | | | II | DAPK3 (+304) | | | |  |
| cg09897131 | 0.635 | 0.733 | 0.098 | 0.000314 | Chr12:6578606 | N_Shore | Body | | |  | VAMP1 | | | II | VAMP1 (+1236), TAPBPL (+17430) | | | |  |
| cg06849501 | 0.604 | 0.521 | -0.083 | 0.000317 | Chr11:70458964 |  | Body | | |  | SHANK2 | | | II | CTTN (+214353), SHANK2 (+476877) | | | |  |
| cg09793619 | 0.786 | 0.825 | 0.039 | 0.000327 | Chr10:131650331 |  | Body | | |  | EBF3 | | | II | EBF3 (+111759), MGMT (+384878) | | | |  |
| cg27235340 | 0.740 | 0.789 | 0.048 | 0.000330 | Chr6:130716157 |  |  | | |  |  | | | II | TMEM200A (-42104), L3MBTL3 (+376430) | | | |  |
| cg01359987 | 0.820 | 0.859 | 0.038 | 0.000331 | Chr6:108281908 | S_Shelf |  | | |  |  | | | II | SEC63 (-2427) | | | |  |
| cg17489312 | 0.287 | 0.223 | -0.064 | 0.000343 | Chr1:9376039 |  | 5'UTR | | |  | SPSB1 | | | II | SLC25A33 (-223488), SPSB1 (+23099) | | | |  |
| cg14333539 | 0.569 | 0.457 | -0.112 | 0.000343 | Chr2:242499512 | S_Shore | Body | | |  | BOK | | | II | BOK (+1367), THAP4 (+77212) | | | |  |
| cg06388350 | 0.230 | 0.182 | -0.047 | 0.000343 | Chr4:95334135 |  |  | | |  |  | | | II | HPGDS (-70109), PDLIM5 (-38902) | | | |  |
| cg10839663 | 0.059 | 0.065 | 0.006 | 0.000345 | Chr1:113261613 | Island |  | | |  |  | | | I | PPM1J (-3664), FAM19A3 (-1575) | | | |  |
| cg25734864 | 0.755 | 0.828 | 0.073 | 0.000366 | Chr13:113812986 |  | 1stExon | | |  | PROZ | | | II | PROZ (+19) | | | |  |
| cg23167585 | 0.052 | 0.069 | 0.017 | 0.000369 | Chr6:20401190 | N_Shore | TSS1500 | | |  | E2F3 | | | II | E2F3 (-946) | | | |  |
| cg24162603 | 0.559 | 0.619 | 0.060 | 0.000384 | Chr3:127945858 |  | Body | | |  | EEFSEC | | | II | EEFSEC (+73546), DNAJB8 (+240232) | | | |  |
| cg04221650 | 0.799 | 0.853 | 0.054 | 0.000386 | Chr1:152681887 |  | 1stExon;3'UTR | | |  | LCE4A;LCE4A | | | II | LCE4A (+365) | | | |  |
| cg21944224 | 0.214 | 0.149 | -0.065 | 0.000387 | Chr3:184301211 | Island |  | | |  |  | | | II | VPS8 (-228719), EPHB3 (+21625) | | | |  |
| cg00534380 | 0.353 | 0.300 | -0.052 | 0.000389 | Chr2:101766586 | N_Shore | Body | | |  | TBC1D8 | | | II | TBC1D8 (+1259), RPL31 (+147896) | | | |  |
| cg26332740 | 0.300 | 0.399 | 0.099 | 0.000393 | Chr1:228196227 | Island | Body | | |  | WNT3A | | | I | ARF1 (-74133), WNT3A (+1505) | | | |  |
| cg26603910 | 0.800 | 0.741 | -0.059 | 0.000400 | Chr17:81014009 | S_Shore |  | | | rs11658970 |  | | | II | B3GNTL1 (-4324) | | | |  |
| cg09553242 | 0.214 | 0.180 | -0.034 | 0.000404 | Chr5:72597015 | N_Shore |  | | |  |  | | | II | TMEM174 (+127993), FOXD1 (+147336) | | | |  |
| cg17937070 | 0.849 | 0.873 | 0.023 | 0.000406 | Chr2:102766322 |  |  | | |  |  | | | II | IL1R1 (-4078) | | | |  |
| cg10427040 | 0.040 | 0.048 | 0.008 | 0.000410 | Chr17:74261306 | Island | 1stExon;5'UTR | | |  | FAM100B | | | I | RNF157 (-24917), PRPSAP1 (+88923) | | | |  |
| cg27181471 | 0.794 | 0.827 | 0.033 | 0.000418 | Chr1:4794111 |  | Body | | | rs442170 | AJAP1 | | | II | AJAP1 (+79007) | | | |  |
| cg05312305 | 0.095 | 0.069 | -0.026 | 0.000428 | Chr7:99156387 | Island | 1stExon;TSS200;5'UTR | | |  | ZNF655 | | | I | ZNF655 (-60) | | | |  |
| cg00620552 | 0.093 | 0.081 | -0.012 | 0.000430 | Chr2:48010097 | Island | TSS200 | | |  | MSH6 | | | I | MSH6 (-123) | | | |  |
| cg25907138 | 0.723 | 0.652 | -0.071 | 0.000431 | Chr2:206730974 |  |  | | |  |  | | | II | NRP2 (+183751), INO80D (+219931) | | | |  |
| cg00510149 | 0.335 | 0.245 | -0.090 | 0.000432 | Chr11:10674966 |  | 5'UTR;TSS1500 | | |  | MRVI1 | | | II | MRVI1 (-1119) | | | |  |
| cg21308632 | 0.564 | 0.482 | -0.082 | 0.000445 | Chr8:127964235 |  |  | | |  |  | | | II | POU5F1B (-463621), FAM84B (-393525) | | | |  |
| cg06036236 | 0.600 | 0.508 | -0.091 | 0.000445 | Chr1:872737 | S_Shore | Body | | |  | SAMD11 | | | II | SAMD11 (+11617), NOC2L (+21941) | | | |  |
| cg13847322 | 0.382 | 0.324 | -0.058 | 0.000448 | Chr9:137272766 |  | Body | | |  | RXRA | | | II | COL5A1 (-260885), RXRA (+54451) | | | |  |
| cg15859496 | 0.454 | 0.324 | -0.130 | 0.000449 | Chr12:118490276 |  | Body | | |  | WSB2 | | | II | WSB2 (+8674), RFC5 (+35771) | | | |  |
| cg00831247 | 0.788 | 0.819 | 0.031 | 0.000450 | Chr2:74780268 | N_Shore | 5'UTR | | |  | LOXL3 | | | II | DOK1 (-1243), LOXL3 (+793) | | | |  |
| cg14235811 | 0.169 | 0.115 | -0.054 | 0.000454 | Chr9:37903063 | N_Shore | 5'UTR;Body | | |  | MCART1 | | | II | MCART1 (+1286), DCAF10 (+102274) | | | |  |
| cg00084687 | 0.065 | 0.077 | 0.012 | 0.000455 | Chr7:92076896 | Island | 1stExon;5'UTR | | |  | GATAD1 | | | I | GATAD1 (+132) | | | |  |
| cg12734024 | 0.822 | 0.854 | 0.032 | 0.000456 | Chr6:2030637 |  | Body | | |  | GMDS | | | II | GMDS (+215230), FOXC1 (+419957) | | | |  |
| cg22791543 | 0.721 | 0.784 | 0.063 | 0.000460 | Chr5:127416532 | N_Shelf | Body | | |  | FLJ33630 | | | II | SLC12A2 (-2950) | | | |  |
| cg13728394 | 0.391 | 0.302 | -0.090 | 0.000468 | Chr3:44115791 |  |  | | |  |  | | | II | C3orf23 (-264152), ABHD5 (+383417) | | | |  |
| cg03127388 | 0.515 | 0.379 | -0.135 | 0.000469 | Chr1:204195149 |  | Body | | |  | PLEKHA6 | | | II | GOLT1A (-11930), PPP1R15B (+185794) | | | |  |
| cg05241431 | 0.717 | 0.777 | 0.060 | 0.000472 | Chr16:49895551 | S_Shelf |  | | |  |  | | | II | TMEM188 (-163637), ZNF423 (-34634) | | | |  |
| cg26682118 | 0.113 | 0.096 | -0.018 | 0.000474 | Chr3:135969063 | Island | TSS200 | | |  | PCCB | | | I | PCCB (-103) | | | |  |
| cg02224845 | 0.217 | 0.248 | 0.031 | 0.000478 | Chr7:143106001 |  | TSS200 | | |  | EPHA1 | | | I | EPHA1 (-17) | | | |  |
| cg04514308 | 0.767 | 0.705 | -0.062 | 0.000480 | Chr3:134077285 | N_Shelf | Body | | |  | AMOTL2 | | | II | RYK (-107700), AMOTL2 (+16120) | | | |  |
| cg20074340 | 0.634 | 0.513 | -0.121 | 0.000483 | Chr13:113651420 | S_Shore | Body | | |  | MCF2L | | | II | F7 (-108684), MCF2L (+27886) | | | |  |
| cg14763172 | 0.867 | 0.883 | 0.015 | 0.000485 | Chr19:6368852 | N_Shelf | 3'UTR | | |  | CLPP | | | II | ALKBH7 (-3591) | | | |  |
| cg13514538 | 0.922 | 0.898 | -0.025 | 0.000489 | Chr8:144942538 | Island | 1stExon | | |  | EPPK1 | | | I | NRBP2 (-19393), EPPK1 (+4895) | | | |  |
| cg18258412 | 0.684 | 0.589 | -0.095 | 0.000492 | Chr2:65803291 |  |  | | |  |  | | | II | MEIS1 (-859240), SPRED2 (-143636) | | | |  |
| cg02341578 | 0.702 | 0.634 | -0.068 | 0.000492 | Chr8:493635 | N_Shore | Body | | |  | C8orf42 | | | II | C8orf42 (+1695), FBXO25 (+136828) | | | |  |
| cg02103895 | 0.895 | 0.928 | 0.033 | 0.000493 | Chr6:127665881 | S_Shore | TSS1500 | | |  | ECHDC1 | | | II | ECHDC1 (-2330) | | | |  |
| cg05358815 | 0.049 | 0.058 | 0.010 | 0.000495 | Chr2:200324281 | Island | 5'UTR | | |  | SATB2 | | | I | SATB2 (+5549) | | | |  |
| cg22825191 | 0.601 | 0.486 | -0.115 | 0.000496 | Chr19:57610536 | N_Shore |  | | |  |  | | | II | PEG3 (-258443), USP29 (-20972) | | | |  |
| cg03862437 | 0.632 | 0.547 | -0.085 | 0.000498 | Chr3:194353432 | N_Shore | Body | | |  | TMEM44 | | | I | TMEM44 (+717) | | | |  |
| cg02774334 | 0.428 | 0.330 | -0.098 | 0.000506 | Chr1:156123500 |  | 5'UTR;1stExon | | |  | SEMA4A | | | II | SEMA4A (+179) | | | |  |
| cg15158086 | 0.444 | 0.373 | -0.071 | 0.000513 | Chr11:1221572 | Island |  | | |  |  | | | II | MUC5B (-22722), MUC2 (+146698) | | | |  |
| cg24474279 | 0.770 | 0.697 | -0.073 | 0.000522 | Chr1:173579110 |  | 3'UTR | | |  | ANKRD45 | | | II | KLHL20 (-104969), SLC9A11 (-6878) | | | |  |
| cg05135015 | 0.135 | 0.109 | -0.026 | 0.000523 | Chr1:153963304 | Island | Body | | |  | RPS27 | | | I | RAB13 (-4499), RPS27 (+66) | | | |  |
| cg07419575 | 0.136 | 0.108 | -0.028 | 0.000526 | Chr2:119605959 | N_Shore | TSS1500 | | |  | EN1 | | | II | EN1 (-201) | | | |  |
| cg07914457 | 0.548 | 0.602 | 0.053 | 0.000533 | Chr1:27241853 |  | TSS1500 | | |  | NR0B2 | | | II | NR0B2 (-1287) | | | |  |
| cg25072179 | 0.566 | 0.649 | 0.083 | 0.000534 | Chr11:57004934 |  | TSS200 | | |  | APLNR | | | II | APLNR (-8) | | | |  |
| cg20539142 | 0.350 | 0.247 | -0.103 | 0.000545 | Chr8:42623718 |  | TSS200 | | |  | CHRNA6 | | | II | CHRNA6 (+210) | | | |  |
| cg00387170 | 0.188 | 0.218 | 0.030 | 0.000545 | Chr6:41514002 | Island | TSS200 | | |  | FOXP4 | | | II | FOXP4 (-161) | | | |  |
| cg09706512 | 0.052 | 0.041 | -0.011 | 0.000547 | Chr3:88108503 | Island | TSS1500 | | |  | CGGBP1;CGGBP1 | | | I | CGGBP1 (-358) | | | |  |
| cg00136643 | 0.156 | 0.179 | 0.023 | 0.000548 | Chr12:72057993 | S_Shore | TSS1500;1stExon;5'UTR | | |  | ZFC3H1;THAP2 | | | I | ZFC3H1 (-245), THAP2 (+317) | | | |  |
| cg13911392 | 0.321 | 0.282 | -0.040 | 0.000550 | Chr19:10445124 | Island | TSS1500;Body | | |  | RAVER1;ICAM3 | | | II | RAVER1 (-811) | | | |  |
| cg01033209 | 0.820 | 0.855 | 0.035 | 0.000552 | Chr14:90531016 | S_Shelf | Body | | |  | KCNK13 | | | II | PSMC1 (-191877), KCNK13 (+2909) | | | |  |
| cg21987785 | 0.424 | 0.340 | -0.084 | 0.000561 | Chr19:36019198 |  | 5'UTR;1stExon | | |  | SBSN | | | II | SBSN (+54) | | | |  |
| cg04282082 | 0.345 | 0.246 | -0.099 | 0.000564 | Chr9:124988720 | Island | Body | | |  | LHX6;LHX6 | | | II | NDUFA8 (-66623), LHX6 (+2370) | | | |  |
| cg23329156 | 0.054 | 0.061 | 0.007 | 0.000574 | Chr3:190231815 | Island | TSS200 | | |  | IL1RAP | | | I | IL1RAP (-24) | | | |  |
| cg15817960 | 0.202 | 0.269 | 0.067 | 0.000577 | Chr12:54409599 | S_Shore | TSS1500 | | |  | HOXC4;HOXC6;HOXC5 | | | II | HOXC6 (-12594), HOXC8 (+6710) | | | |  |
| cg22379915 | 0.052 | 0.047 | -0.005 | 0.000579 | Chr17:39684287 | Island | 1stExon | | |  | KRT19 | | | I | KRT15 (-9018), KRT9 (+44022) | | | |  |
| cg21471199 | 0.580 | 0.687 | 0.107 | 0.000586 | Chr1:19218212 |  | TSS1500;Body | | |  | ALDH4A1 | | | II | TAS1R2 (-32058), ALDH4A1 (+11080) | | | |  |
| cg09672452 | 0.521 | 0.592 | 0.071 | 0.000592 | Chr15:55665236 |  | Body;TSS200 | | |  | CCPG1;MIR628 | | | II | CCPG1 (+35471), PIGB (+54104) | | | |  |
| cg14322224 | 0.173 | 0.251 | 0.079 | 0.000600 | Chr1:85931226 | S_Shore | TSS1500;5'UTR | | |  | DDAH1 | | | II | DDAH1 (-338) | | | |  |
| cg16332065 | 0.330 | 0.276 | -0.054 | 0.000606 | Chr4:46126253 |  | TSS200 | | |  | GABRG1 | | | II | GABRG1 (-172) | | | |  |
| cg19683780 | 0.725 | 0.787 | 0.061 | 0.000613 | Chr8:42907576 | N_Shelf |  | | |  |  | | | II | FNTA (-3865) | | | |  |
| cg20566657 | 0.408 | 0.349 | -0.059 | 0.000621 | Chr4:10021355 | S_Shore | Body | | |  | SLC2A9 | | | II | SLC2A9 (+1758), DRD5 (+238098) | | | |  |
| cg00182273 | 0.360 | 0.296 | -0.064 | 0.000622 | Chr16:66876847 | N_Shore | TSS1500 | | |  | CA7 | | | II | CA7 (-1434) | | | |  |
| cg01602001 | 0.568 | 0.525 | -0.043 | 0.000624 | Chr16:65104932 |  | 5'UTR | | |  | CDH11 | | | II | CDH11 (+50986) | | | |  |
| cg15721238 | 0.497 | 0.418 | -0.079 | 0.000624 | Chr3:196520408 |  | Body | | |  | PAK2 | | | II | SENP5 (-74318), PAK2 (+53681) | | | |  |
| cg23396834 | 0.121 | 0.104 | -0.017 | 0.000624 | Chr5:76383032 | Island | Body;TSS200 | | |  | LOC728723;ZBED3 | | | I | ZBED3 (-3) | | | |  |
| cg01218431 | 0.834 | 0.870 | 0.036 | 0.000625 | Chr13:32527522 | S_Shore | Body | | | rs916756 | EEF1DP3 | | | II | FRY (-77914), RXFP2 (+213844) | | | |  |
| cg14596967 | 0.400 | 0.472 | 0.072 | 0.000625 | Chr11:113956944 | S_Shelf | Body | | |  | ZBTB16;ZBTB16 | | | II | NNMT (-209590), ZBTB16 (+25657) | | | |  |
| cg04287289 | 0.047 | 0.053 | 0.007 | 0.000629 | Chr16:89883240 | Island | TSS200 | | |  | FANCA;FANCA | | | I | FANCA (-176) | | | |  |
| cg03147185 | 0.353 | 0.278 | -0.075 | 0.000633 | Chr2:97008030 |  | Body | | |  | NCAPH | | | II | NCAPH (+6547), NEURL3 (+163063) | | | |  |
| cg11961813 | 0.247 | 0.212 | -0.035 | 0.000633 | Chr17:40995546 |  | 3'UTR;TSS1500 | | |  | PSME3;AOC2 | | | II | AOC2 (-1062) | | | |  |
| cg01101677 | 0.752 | 0.688 | -0.064 | 0.000645 | Chr2:44313980 | N_Shore |  | | |  |  | | | II | LRPPRC (-90837), PPM1B (-82019) | | | |  |
| cg06367659 | 0.100 | 0.137 | 0.038 | 0.000647 | Chr22:50919817 | Island | TSS200 | | |  | ADM2 | | | I | ADM2 (-167) | | | |  |
| cg06713671 | 0.348 | 0.274 | -0.074 | 0.000656 | Chr19:5914158 | N_Shore | TSS200 | | |  | CAPS | | | II | CAPS (-34) | | | |  |
| cg21889914 | 0.756 | 0.681 | -0.076 | 0.000657 | Chr15:85504254 |  |  | | |  |  | | | II | PDE8A (-20950), SLC28A1 (+76342) | | | |  |
| cg20471615 | 0.917 | 0.869 | -0.048 | 0.000658 | Chr9:139328837 | Island | Body | | |  | INPP5E | | | II | INPP5E (+5418), PMPCA (+23722) | | | |  |
| cg04365721 | 0.567 | 0.676 | 0.110 | 0.000661 | Chr5:3600391 | Island | Body | | |  | IRX1 | | | II | IRX1 (+4224) | | | |  |
| cg24680415 | 0.758 | 0.688 | -0.070 | 0.000661 | Chr17:70358572 |  |  | | |  |  | | | II | SOX9 (+241412), SLC39A11 (+730280) | | | |  |
| cg15129052 | 0.646 | 0.583 | -0.063 | 0.000663 | Chr1:55416755 |  |  | | |  |  | | | II | DHCR24 (-63835), TMEM61 (-29709) | | | |  |
| cg08305189 | 0.097 | 0.105 | 0.008 | 0.000666 | Chr10:99257674 | N_Shore | TSS1500;Body | | |  | UBTD1;MMS19 | | | I | MMS19 (+691) | | | |  |
| cg02057917 | 0.639 | 0.586 | -0.053 | 0.000668 | Chr6:29323269 |  | 1stExon | | |  | OR5V1 | | | II | OR5V1 (+784) | | | |  |
| cg10396171 | 0.414 | 0.277 | -0.137 | 0.000668 | Chr16:605214 | Island |  | | |  |  | | | I | PIGQ (-14753), SOLH (+27359) | | | |  |
| cg27449360 | 0.167 | 0.197 | 0.030 | 0.000674 | Chr4:57688308 | S_Shore | TSS1500 | | |  | SPINK2 | | | II | SPINK2 (-416) | | | |  |
| cg06955214 | 0.854 | 0.878 | 0.025 | 0.000676 | Chr7:157968683 |  | Body | | |  | PTPRN2 | | | I | PTPRN2 (+411798), DNAJB6 (+838974) | | | |  |
| cg23118464 | 0.238 | 0.192 | -0.045 | 0.000677 | Chr2:100721844 | S_Shore | 5'UTR | | |  | AFF3;AFF3 | | | I | AFF3 (+200) | | | |  |
| cg21187770 | 0.550 | 0.419 | -0.131 | 0.000679 | Chr2:26205876 | S_Shore | TSS1500 | | |  | KIF3C | | | I | KIF3C (-434) | | | |  |
| cg16300553 | 0.488 | 0.364 | -0.124 | 0.000681 | Chr8:8131077 |  |  | | |  |  | | | II | SGK223 (+108179), LOC100132396 (+347219) | | | |  |
| cg20869658 | 0.604 | 0.670 | 0.066 | 0.000682 | Chr2:178481033 | N_Shelf | 1stExon;3'UTR | | |  | TTC30A | | | II | TTC30B (-63510), TTC30A (+2660) | | | |  |
| cg07345919 | 0.159 | 0.180 | 0.021 | 0.000698 | Chr6:8102919 | S_Shore | TSS200 | | |  | EEF1E1 | | | I | EEF1E1 (-92) | | | |  |
| cg08995061 | 0.664 | 0.740 | 0.076 | 0.000701 | Chr2:174129462 |  | Body | | |  | ZAK | | | II | CDCA7 (-90098), ZAK (+188898) | | | |  |
| cg17182240 | 0.502 | 0.386 | -0.117 | 0.000709 | Chr11:126768919 |  | Body | | |  | KIRREL3 | | | II | KIRREL3 (+101846), ST3GAL4 (+543380) | | | |  |
| cg03504496 | 0.916 | 0.894 | -0.021 | 0.000715 | Chr22:40289577 |  | 5'UTR | | |  | ENTHD1 | | | I | GRAP2 (-7508), CACNA1I (+322820) | | | |  |
| cg14383238 | 0.174 | 0.218 | 0.044 | 0.000718 | Chr14:77647983 | N_Shore | TSS200 | | |  | TMEM63C | | | I | TMEM63C (-118) | | | |  |
| cg03967448 | 0.475 | 0.361 | -0.114 | 0.000721 | Chr6:3527443 |  |  | | |  |  | | | II | SLC22A23 (-70651), PXDC1 (+224802) | | | |  |
| cg15717853 | 0.118 | 0.101 | -0.018 | 0.000724 | Chr1:182573596 | Island | TSS200 | | |  | RGS16 | | | I | RGS16 (-49) | | | |  |
| cg04674803 | 0.087 | 0.068 | -0.020 | 0.000725 | Chr13:28395390 |  |  | | |  |  | | | I | PDX1 (-98777), GSX1 (+28611) | | | |  |
| cg27130075 | 0.771 | 0.814 | 0.043 | 0.000736 | Chr13:110935325 |  | Body | | |  | COL4A1 | | | II | IRS2 (-496412), COL4A1 (+24170) | | | |  |
| cg13245228 | 0.440 | 0.373 | -0.067 | 0.000739 | Chr8:143537415 | S_Shore |  | | |  |  | | | II | TSNARE1 (-52873), BAI1 (-7961) | | | |  |
| cg01404197 | 0.111 | 0.132 | 0.021 | 0.000747 | Chr11:113644652 | Island | TSS1500 | | |  | ZW10 | | | II | ZW10 (-168) | | | |  |
| cg01147107 | 0.173 | 0.229 | 0.056 | 0.000749 | Chr1:47696505 | Island | TSS1500 | | |  | TAL1 | | | II | TAL1 (-1063) | | | |  |
| cg10408284 | 0.794 | 0.865 | 0.071 | 0.000752 | Chr17:48912327 |  | TSS1500 | | |  | WFIKKN2 | | | I | WFIKKN2 (-277) | | | |  |
| cg19551485 | 0.350 | 0.242 | -0.108 | 0.000753 | Chr3:55693182 |  | Body;3'UTR | | |  | C3orf51;ERC2 | | | II | WNT5A (-171852), ERC2 (+809208) | | | |  |
| cg18725076 | 0.099 | 0.124 | 0.025 | 0.000764 | Chr5:11904912 | S_Shore | TSS1500 | | |  | CTNND2 | | | I | CTNND2 (-803) | | | |  |
| cg14224937 | 0.103 | 0.085 | -0.018 | 0.000768 | Chr19:52873101 | Island | TSS200 | | |  | ZNF880 | | | I | ZNF880 (-68) | | | |  |
| cg26917132 | 0.056 | 0.066 | 0.010 | 0.000769 | Chr16:87425759 | Island | TSS200 | | |  | FBXO31;MAP1LC3B | | | I | MAP1LC3B (-41) | | | |  |
| cg04370737 | 0.121 | 0.144 | 0.023 | 0.000772 | Chr14:93799041 | Island | 5'UTR;TSS1500 | | |  | BTBD7;KIAA1409 | | | I | UNC79 (-523) | | | |  |
| cg12521736 | 0.444 | 0.351 | -0.093 | 0.000773 | Chr10:773141 | S_Shore |  | | |  |  | | | II | DIP2C (-37534), LARP4B (+158560) | | | |  |
| cg04969878 | 0.632 | 0.701 | 0.069 | 0.000774 | Chr12:72079240 | N_Shore | TSS1500 | | |  | TMEM19 | | | II | TMEM19 (-637) | | | |  |
| cg21222826 | 0.216 | 0.249 | 0.033 | 0.000776 | Chr3:69062762 | Island | 1stExon;5'UTR | | |  | C3orf64;C3orf64 | | | I | C3orf64 (+11) | | | |  |
| cg00030427 | 0.366 | 0.289 | -0.078 | 0.000779 | Chr6:169562540 |  |  | | |  |  | | | II | THBS2 (+91596), SMOC2 (+720710) | | | |  |
| cg02542218 | 0.404 | 0.319 | -0.085 | 0.000780 | Chr7:131630566 | S_Shore |  | | |  |  | | | II | PODXL (-389191), PLXNA4 (+630756) | | | |  |
| cg21452942 | 0.490 | 0.396 | -0.094 | 0.000788 | Chr17:78056120 |  | Body | | |  | CCDC40 | | | II | GAA (-19234), CCDC40 (+45690) | | | |  |
| cg13524037 | 0.551 | 0.639 | 0.088 | 0.000789 | Chr6:32904074 |  | Body | | |  | HLA-DMB | | | II | HLA-DMB (+4772), PSMB9 (+82137) | | | |  |
| cg06581938 | 0.435 | 0.408 | -0.027 | 0.000791 | Chr7:64403228 |  |  | | |  |  | | | II | ZNF273 (+39609), ZNF117 (+48185) | | | |  |
| cg23959976 | 0.545 | 0.611 | 0.066 | 0.000791 | Chr19:41187143 | N_Shelf | Body | | |  | NUMBL | | | II | NUMBL (+9412), LTBP4 (+84003) | | | |  |
| cg15392819 | 0.434 | 0.320 | -0.114 | 0.000792 | Chr1:180585679 |  |  | | |  |  | | | II | ACBD6 (-113658), XPR1 (-15466) | | | |  |
| cg19778822 | 0.501 | 0.383 | -0.118 | 0.000794 | Chr18:11269050 |  |  | | |  |  | | | II | GNAL (-420085), PIEZO2 (-120290) | | | |  |
| cg07870920 | 0.763 | 0.684 | -0.079 | 0.000802 | Chr4:121569769 |  |  | | | . |  | | | II | MAD2L1 (-581757), PRDM5 (+274243) | | | |  |
| cg19826026 | 0.216 | 0.290 | 0.073 | 0.000805 | Chr12:15114393 |  | 5'UTR | | |  | ARHGDIB | | | II | ARHGDIB (+168) | | | |  |
| cg26112639 | 0.456 | 0.511 | 0.055 | 0.000806 | Chr1:247580106 |  | TSS1500;5'UTR | | |  | NLRP3 | | | II | NLRP3 (+649) | | | |  |
| cg07882831 | 0.060 | 0.072 | 0.012 | 0.000806 | Chr17:72209802 | Island | TSS1500;1stExon | | |  | MGC16275;TTYH2 | | | I | TTYH2 (+107) | | | |  |
| cg13168317 | 0.642 | 0.576 | -0.065 | 0.000806 | Chr8:2177425 |  |  | | | rs4875908 |  | | | I | MYOM2 (+184268) | | | |  |
| cg13630114 | 0.466 | 0.395 | -0.071 | 0.000808 | Chr9:102208423 |  |  | | |  |  | | | II | NR4A3 (-375713), SEC61B (+223854) | | | |  |
| cg22215728 | 0.089 | 0.066 | -0.023 | 0.000818 | Chr3:61236652 | Island | 5'UTR | | |  | FHIT | | | II | FHIT (+480) | | | |  |
| cg17280828 | 0.457 | 0.428 | -0.029 | 0.000819 | Chr1:149284924 | N_Shore | Body | | |  | LOC388692 | | | II | PPIAL4B (-268078), PPIAL4F (+478910) | | | |  |
| cg10991752 | 0.741 | 0.675 | -0.066 | 0.000823 | Chr6:155845314 |  |  | | |  |  | | | II | NOX3 (-68278) | | | |  |
| cg22620614 | 0.856 | 0.889 | 0.033 | 0.000833 | Chr11:70794709 |  | Body | | |  | SHANK2 | | | II | SHANK2 (+141132), CTTN (+550098) | | | |  |
| cg00671823 | 0.371 | 0.322 | -0.050 | 0.000837 | Chr16:27558770 | N_Shelf | Body | | |  | GTF3C1 | | | II | GTF3C1 (+2480), IL21R (+120192) | | | |  |
| cg01950845 | 0.061 | 0.075 | 0.014 | 0.000837 | Chr15:93632730 | Island | TSS1500 | | |  | RGMA | | | I | RGMA (-15515) | | | |  |
| cg07026454 | 0.045 | 0.051 | 0.006 | 0.000840 | Chr8:145582391 | Island | 5'UTR;TSS1500;1stExon | | |  | GPR172A;FBXL6 | | | I | C8ORFK29 (-3818), FBXL6 (-209), GPR172A (+116) | | | |  |
| cg02436605 | 0.642 | 0.566 | -0.076 | 0.000859 | Chr8:146125223 | N_Shore | 5'UTR | | |  | ZNF250;ZNF250 | | | II | COMMD5 (-46773), ZNF250 (+1622) | | | |  |
| cg26342177 | 0.815 | 0.844 | 0.029 | 0.000862 | Chr22:36113512 |  | TSS1500 | | |  | APOL5 | | | II | APOL5 (-406) | | | |  |
| cg20319604 | 0.831 | 0.783 | -0.048 | 0.000864 | Chr2:44428936 |  | Body;5'UTR | | |  | PPM1B | | | II | SLC3A1 (-73660), PPM1B (+32937) | | | |  |
| cg13079429 | 0.705 | 0.776 | 0.071 | 0.000865 | Chr11:28781311 |  |  | | |  |  | | | II | METTL15 (+651514) | | | |  |
| cg18159583 | 0.069 | 0.081 | 0.012 | 0.000875 | Chr7:87505730 | Island | TSS200;1stExon;5'UTR | | |  | SLC25A40;DBF4 | | | I | SLC25A40 (-39), DBF4 (+187) | | | |  |
| cg21540530 | 0.555 | 0.625 | 0.070 | 0.000879 | Chr2:188583082 |  |  | | |  |  | | | II | GULP1 (-573313), TFPI (-163864) | | | |  |
| cg19434199 | 0.930 | 0.904 | -0.026 | 0.000886 | Chr21:48018608 | Island | 3'UTR | | |  | S100B | | | I | S100B (+6426), DIP2A (+139747) | | | |  |
| cg00638020 | 0.546 | 0.395 | -0.151 | 0.000886 | Chr7:158281138 | N_Shore | Body | | |  | PTPRN2 | | | II | PTPRN2 (+99343) | | | |  |
| cg05433152 | 0.770 | 0.803 | 0.033 | 0.000894 | Chr3:45299618 |  |  | | |  |  | | | II | LARS2 (-130456), TMEM158 (-31805) | | | |  |
| cg27364650 | 0.507 | 0.595 | 0.087 | 0.000897 | Chr3:43899898 |  |  | | |  |  | | | II | C3orf23 (-480045), ABHD5 (+167524) | | | |  |
| cg14945086 | 0.646 | 0.712 | 0.065 | 0.000902 | Chr18:2657780 | S_Shore | Body | | |  | SMCHD1 | | | II | EMILIN2 (-189247), SMCHD1 (+1895) | | | |  |
| cg06234342 | 0.113 | 0.084 | -0.029 | 0.000906 | Chr6:111279552 | N_Shore | TSS1500 | | |  | GTF3C6 | | | II | GTF3C6 (-210) | | | |  |
| cg22443212 | 0.414 | 0.497 | 0.083 | 0.000930 | Chr17:78253912 |  | Body | | |  | RNF213 | | | II | ENDOV (-135054), RNF213 (+19246) | | | |  |
| cg00014301 | 0.846 | 0.815 | -0.031 | 0.000932 | Chr2:3327027 |  | Body | | |  | TSSC1 | | | II | MYT1L (-991983), TRAPPC12 (-56418) | | | |  |
| cg24333469 | 0.199 | 0.167 | -0.032 | 0.000935 | Chr22:21386894 | Island | TSS200 | | |  | SLC7A4 | | | I | SLC7A4 (-48) | | | |  |
| cg17878881 | 0.884 | 0.835 | -0.049 | 0.000938 | Chr12:125349072 | S_Shore | TSS1500 | | |  | SCARB1 | | | II | SCARB1 (-554) | | | |  |
| cg23968676 | 0.088 | 0.075 | -0.013 | 0.000942 | Chr11:118973105 | S_Shore | TSS1500 | | |  | DPAGT1 | | | II | C2CD2L (-4987), DPAGT1 (-321) | | | |  |
| cg12080391 | 0.134 | 0.103 | -0.030 | 0.000943 | Chr12:5018984 | Island | TSS200 | | |  | KCNA1 | | | I | KCNA1 (-88) | | | |  |
| cg10355989 | 0.537 | 0.568 | 0.032 | 0.000947 | Chr2:9143982 | Island | TSS200 | | |  | MBOAT2 | | | I | MBOAT2 (-107) | | | |  |
| cg03544499 | 0.151 | 0.100 | -0.051 | 0.000948 | Chr9:85677016 | Island | Body | | |  | RASEF | | | II | RASEF (+1026) | | | |  |
| cg01577760 | 0.461 | 0.361 | -0.100 | 0.000948 | Chr11:67776123 |  | 1stExon;5'UTR | | |  | ALDH3B1 | | | I | UNC93B1 (-4531), ALDH3B1 (-1666) | | | |  |
| cg18713687 | 0.590 | 0.695 | 0.105 | 0.000954 | Chr3:195489789 | Island | Body | | |  | MUC4 | | | II | MUC20 (+42037), MUC4 (+49054) | | | |  |
| cg03766851 | 0.574 | 0.487 | -0.087 | 0.000955 | Chr2:47141740 | N_Shore | Body;5'UTR | | |  | MCFD2 | | | II | MCFD2 (+1510), SOCS5 (+215642) | | | |  |
| cg01173332 | 0.582 | 0.500 | -0.082 | 0.000959 | Chr5:1672185 |  |  | | |  |  | | | II | LPCAT1 (-148110), MRPL36 (+127770) | | | |  |
| cg07743144 | 0.830 | 0.862 | 0.033 | 0.000961 | Chr6:31855706 | N_Shore | Body | | |  | EHMT2 | | | II | SLC44A4 (-8884), EHMT2 (+9757) | | | |  |
| cg06440646 | 0.138 | 0.164 | 0.026 | 0.000964 | Chr4:146403418 | Island | TSS1500;5'UTR | | |  | SMAD1 | | | I | SMAD1 (+468) | | | |  |
| cg18983818 | 0.552 | 0.459 | -0.093 | 0.000966 | Chr17:21176176 | N_Shelf |  | | |  |  | | | I | TMEM11 (-58269), MAP2K3 (-11791) | | | |  |
| cg25784220 | 0.055 | 0.046 | -0.009 | 0.000969 | Chr19:58609602 | Island | Body1stExon;5'UTR | | |  | ZSCAN18 | | | I | ZSCAN18 (+20190), ZNF135 (+38996) | | | |  |
| cg04405075 | 0.800 | 0.748 | -0.052 | 0.000973 | Chr20:2796872 |  | TSS200 | | |  | LOC100288797 | | | I | TMEM239 (-103) | | | |  |
| cg21466315 | 0.413 | 0.365 | -0.048 | 0.000975 | Chr3:133294854 | S_Shore | Body | | |  | CDV3;CDV3;CDV3 | | | II | CDV3 (+2421), TOPBP1 (+85882) | | | |  |
| cg15380607 | 0.122 | 0.137 | 0.014 | 0.000975 | Chr6:138188545 | Island | TSS200 | | |  | TNFAIP3 | | | I | TNFAIP3 (-35) | | | |  |
| cg25990402 | 0.486 | 0.579 | 0.093 | 0.000979 | Chr6:41302256 |  | TSS1500 | | |  | NCR2 | | | II | NCR2 (-1271) | | | |  |
| cg19737468 | 0.121 | 0.106 | -0.015 | 0.000981 | Chr14:24701538 | N_Shore | 1stExon;5'UTR;TSS1500;TSS200 | | |  | NEDD8;GMPR2 | | | I | GMPR2 (-564), NEDD8-MDP1 (+37) | | | |  |
| cg04733933 | 0.648 | 0.742 | 0.093 | 0.000983 | Chr4:7203222 |  | Body | | |  | SORCS2 | | | II | SORCS2 (+8849), PSAPL1 (+233477) | | | |  |
| cg06808763 | 0.638 | 0.678 | 0.040 | 0.000988 | Chr14:106859695 | S_Shore |  | | | rs79159065 |  | | | II | ADAM6 (-421338) | | | |  |
| cg03785728 | 0.394 | 0.317 | -0.077 | 0.000988 | Chr20:17674195 |  | TSS200 | | | rs68085938 | BANF2 | | | II | BANF2 (-124) | | | |  |
| cg18506124 | 0.424 | 0.346 | -0.078 | 0.000991 | Chr19:35629603 | N_Shelf | TSS200;TSS1500 | | |  | FXYD1 | | | II | FXYD7 (-4550), LGI4 (-3426), FXYD1 (-128) | | | |  |
| cg10926432 | 0.786 | 0.831 | 0.044 | 0.000991 | Chr3:133289741 | N_Shelf |  | | |  |  | | | II | CDV3 (-2692) | | | |  |
| cg20460166 | 0.310 | 0.253 | -0.056 | 0.000994 | Chr1:54423202 |  | Body | | |  | LRRC42 | | | II | HSPB11 (-11915), LDLRAD1 (+60600) | | | |  |
| cg06159269 | 0.384 | 0.275 | -0.109 | 0.000994 | Chr1:8767347 | S_Shelf | 5'UTR | | |  | RERE | | | II | RERE (+110351), SLC45A1 (+382958) | | | |  |
| cg06059461 | 0.304 | 0.243 | -0.061 | 0.000997 | Chr15:89960503 | S_Shore |  | | |  |  | | | II | POLG (-82478), RHCG (+79295) | | | |  |
| Ranked by P value. DMPs, differentially methylated positions; MZ, monozygotic; GREAT, Genomic Regions Enrichment of Annotations Tool; TSS, transcription start site. . SBE: Single base extension. ∆β: Change in DNA methylation (Affected twin β – Unaffected twin β). UCSC: University of California Santa Cruz genome browser. SNP: Single nucleotide polymorphism. | | | | | | | | | | | | | | | | | |  |  |

| **Supplementary Table S4. Gene Ontology enrichment analysis for persistent asthma age-10 DMPs** | | |  |  |
| --- | --- | --- | --- | --- |
| **GO Accession ID** | **GO Function** | **Ontology** | | **P value** |
| GO:0042094 | interleukin-2 biosynthetic process | BP | | 0.0009 |
| GO:0046164 | alcohol catabolic process | BP | | 0.0014 |
| GO:0032623 | interleukin-2 production | BP | | 0.0025 |
| GO:1901616 | organic hydroxy compound catabolic process | BP | | 0.0034 |
| GO:0032480 | negative regulation of type I interferon production | BP | | 0.0039 |
| GO:0005890 | sodium:potassium-exchanging ATPase complex | CC | | 0.0044 |
| GO:0006821 | chloride transport | BP | | 0.0046 |
| GO:0052548 | regulation of endopeptidase activity | BP | | 0.0050 |
| GO:0090533 | cation-transporting ATPase complex | CC | | 0.0051 |
| GO:1902495 | transmembrane transporter complex | CC | | 0.0054 |
| GO:1990351 | transporter complex | CC | | 0.0058 |
| GO:0030159 | receptor signaling complex scaffold activity | MF | | 0.0068 |
| GO:0052547 | regulation of peptidase activity | BP | | 0.0069 |
| GO:0016641 | oxidoreductase activity, acting on the CH-NH2 group of donors, oxygen as acceptor | MF | | 0.0070 |
| GO:0042157 | lipoprotein metabolic process | BP | | 0.0071 |
| GO:0005254 | chloride channel activity | MF | | 0.0074 |
| GO:0051607 | defense response to virus | BP | | 0.0075 |
| GO:0008047 | enzyme activator activity | MF | | 0.0076 |
| GO:0035872 | nucleotide-binding domain, leucine rich repeat containing receptor signaling pathway | BP | | 0.0080 |
| GO:0050718 | positive regulation of interleukin-1 beta secretion | BP | | 0.0085 |
| GO:0036445 | neuronal stem cell division | BP | | 0.0087 |
| GO:0055057 | neuroblast division | BP | | 0.0087 |
| GO:0016638 | oxidoreductase activity, acting on the CH-NH2 group of donors | MF | | 0.0088 |
| GO:0050716 | positive regulation of interleukin-1 secretion | BP | | 0.0088 |
| GO:0005253 | anion channel activity | MF | | 0.0091 |
| GO:0043124 | negative regulation of I-kappaB kinase/NF-kappaB signaling | BP | | 0.0096 |
| GO:0050688 | regulation of defense response to virus | BP | | 0.0100 |
| GO:0050706 | regulation of interleukin-1 beta secretion | BP | | 0.0103 |
| GO:0017145 | stem cell division | BP | | 0.0103 |
| GO:0034702 | ion channel complex | CC | | 0.0104 |
| GO:0015079 | potassium ion transmembrane transporter activity | MF | | 0.0105 |
| GO:0010950 | positive regulation of endopeptidase activity | BP | | 0.0105 |
| GO:0050704 | regulation of interleukin-1 secretion | BP | | 0.0106 |
| GO:0005216 | ion channel activity | MF | | 0.0109 |
| GO:0046174 | polyol catabolic process | BP | | 0.0111 |
| GO:0006461 | protein complex assembly | BP | | 0.0112 |
| GO:0042347 | negative regulation of NF-kappaB import into nucleus | BP | | 0.0113 |
| GO:0070271 | protein complex biogenesis | BP | | 0.0114 |
| GO:0022838 | substrate-specific channel activity | MF | | 0.0118 |
| GO:0045076 | regulation of interleukin-2 biosynthetic process | BP | | 0.0126 |
| GO:0061036 | positive regulation of cartilage development | BP | | 0.0130 |
| GO:0050702 | interleukin-1 beta secretion | BP | | 0.0138 |
| GO:0048333 | mesodermal cell differentiation | BP | | 0.0139 |
| GO:0032663 | regulation of interleukin-2 production | BP | | 0.0146 |
| GO:0010952 | positive regulation of peptidase activity | BP | | 0.0146 |
| GO:0098533 | ATPase dependent transmembrane transport complex | CC | | 0.0150 |
| GO:0015698 | inorganic anion transport | BP | | 0.0153 |
| GO:0015267 | channel activity | MF | | 0.0155 |
| GO:0022803 | passive transmembrane transporter activity | MF | | 0.0155 |
| GO:0009595 | detection of biotic stimulus | BP | | 0.0162 |
| GO:0071804 | cellular potassium ion transport | BP | | 0.0163 |
| GO:0071805 | potassium ion transmembrane transport | BP | | 0.0163 |
| GO:0051251 | positive regulation of lymphocyte activation | BP | | 0.0166 |
| GO:0034707 | chloride channel complex | CC | | 0.0172 |
| GO:0050701 | interleukin-1 secretion | BP | | 0.0172 |
| GO:0050829 | defense response to Gram-negative bacterium | BP | | 0.0175 |
| GO:0042073 | intraciliary transport | BP | | 0.0179 |
| GO:1902476 | chloride transmembrane transport | BP | | 0.0181 |
| GO:0043281 | regulation of cysteine-type endopeptidase activity involved in apoptotic process | BP | | 0.0182 |
| GO:0016780 | phosphotransferase activity, for other substituted phosphate groups | MF | | 0.0183 |
| GO:0050870 | positive regulation of T cell activation | BP | | 0.0184 |
| GO:0065003 | macromolecular complex assembly | BP | | 0.0185 |
| GO:0015108 | chloride transmembrane transporter activity | MF | | 0.0193 |
| GO:0032731 | positive regulation of interleukin-1 beta production | BP | | 0.0197 |
| GO:0044282 | small molecule catabolic process | BP | | 0.0201 |
| GO:1900117 | regulation of execution phase of apoptosis | BP | | 0.0206 |
| GO:0022607 | cellular component assembly | BP | | 0.0210 |
| GO:0030901 | midbrain development | BP | | 0.0224 |
| GO:0002696 | positive regulation of leukocyte activation | BP | | 0.0226 |
| GO:2000116 | regulation of cysteine-type endopeptidase activity | BP | | 0.0228 |
| GO:0051249 | regulation of lymphocyte activation | BP | | 0.0237 |
| GO:0008076 | voltage-gated potassium channel complex | CC | | 0.0240 |
| GO:0070663 | regulation of leukocyte proliferation | BP | | 0.0241 |
| GO:0002832 | negative regulation of response to biotic stimulus | BP | | 0.0243 |
| GO:0005097 | Rab GTPase activator activity | MF | | 0.0243 |
| GO:0005096 | GTPase activator activity | MF | | 0.0247 |
| GO:0043687 | post-translational protein modification | BP | | 0.0249 |
| GO:0030220 | platelet formation | BP | | 0.0251 |
| GO:0036344 | platelet morphogenesis | BP | | 0.0251 |
| GO:0005267 | potassium channel activity | MF | | 0.0251 |
| GO:0050671 | positive regulation of lymphocyte proliferation | BP | | 0.0252 |
| GO:0032732 | positive regulation of interleukin-1 production | BP | | 0.0253 |
| GO:0032946 | positive regulation of mononuclear cell proliferation | BP | | 0.0258 |
| GO:2001238 | positive regulation of extrinsic apoptotic signaling pathway | BP | | 0.0258 |
| GO:0005887 | integral component of plasma membrane | CC | | 0.0258 |
| GO:0050867 | positive regulation of cell activation | BP | | 0.0260 |
| GO:0034705 | potassium channel complex | CC | | 0.0260 |
| GO:0031348 | negative regulation of defense response | BP | | 0.0266 |
| GO:0002831 | regulation of response to biotic stimulus | BP | | 0.0270 |
| GO:0034332 | adherens junction organization | BP | | 0.0270 |
| GO:0050715 | positive regulation of cytokine secretion | BP | | 0.0276 |
| GO:0070665 | positive regulation of leukocyte proliferation | BP | | 0.0277 |
| GO:0030162 | regulation of proteolysis | BP | | 0.0283 |
| GO:0043280 | positive regulation of cysteine-type endopeptidase activity involved in apoptotic process | BP | | 0.0288 |
| GO:0016620 | oxidoreductase activity, acting on the aldehyde or oxo group of donors, NAD or NADP as acceptor | MF | | 0.0296 |
| GO:0009303 | rRNA transcription | BP | | 0.0301 |
| GO:0032088 | negative regulation of NF-kappaB transcription factor activity | BP | | 0.0305 |
| GO:0006497 | protein lipidation | BP | | 0.0313 |
| GO:0032648 | regulation of interferon-beta production | BP | | 0.0313 |
| GO:0038023 | signaling receptor activity | MF | | 0.0326 |
| GO:0021527 | spinal cord association neuron differentiation | BP | | 0.0347 |
| GO:0071222 | cellular response to lipopolysaccharide | BP | | 0.0349 |
| GO:0032608 | interferon-beta production | BP | | 0.0349 |
| GO:2001056 | positive regulation of cysteine-type endopeptidase activity | BP | | 0.0355 |
| GO:0098661 | inorganic anion transmembrane transport | BP | | 0.0359 |
| GO:0050775 | positive regulation of dendrite morphogenesis | BP | | 0.0360 |
| GO:0048103 | somatic stem cell division | BP | | 0.0361 |
| GO:0042345 | regulation of NF-kappaB import into nucleus | BP | | 0.0362 |
| GO:0042348 | NF-kappaB import into nucleus | BP | | 0.0362 |
| GO:0005249 | voltage-gated potassium channel activity | MF | | 0.0365 |
| GO:0042158 | lipoprotein biosynthetic process | BP | | 0.0366 |
| GO:0042102 | positive regulation of T cell proliferation | BP | | 0.0366 |
| GO:0003690 | double-stranded DNA binding | MF | | 0.0367 |
| GO:0019048 | modulation by virus of host morphology or physiology | BP | | 0.0375 |
| GO:0009065 | glutamine family amino acid catabolic process | BP | | 0.0377 |
| GO:0001710 | mesodermal cell fate commitment | BP | | 0.0377 |
| GO:0071813 | lipoprotein particle binding | MF | | 0.0381 |
| GO:0071814 | protein-lipid complex binding | MF | | 0.0381 |
| GO:0015075 | ion transmembrane transporter activity | MF | | 0.0381 |
| GO:0072331 | signal transduction by p53 class mediator | BP | | 0.0386 |
| GO:2001236 | regulation of extrinsic apoptotic signaling pathway | BP | | 0.0390 |
| GO:0071219 | cellular response to molecule of bacterial origin | BP | | 0.0396 |
| GO:0015026 | coreceptor activity | MF | | 0.0411 |
| GO:0002026 | regulation of the force of heart contraction | BP | | 0.0420 |
| GO:0044085 | cellular component biogenesis | BP | | 0.0420 |
| GO:0031226 | intrinsic component of plasma membrane | CC | | 0.0420 |
| GO:0006813 | potassium ion transport | BP | | 0.0422 |
| GO:0051345 | positive regulation of hydrolase activity | BP | | 0.0424 |
| GO:0097191 | extrinsic apoptotic signaling pathway | BP | | 0.0437 |
| GO:0032651 | regulation of interleukin-1 beta production | BP | | 0.0444 |
| GO:0004872 | receptor activity | MF | | 0.0445 |
| GO:0048708 | astrocyte differentiation | BP | | 0.0446 |
| GO:0008509 | anion transmembrane transporter activity | MF | | 0.0448 |
| GO:0015103 | inorganic anion transmembrane transporter activity | MF | | 0.0448 |
| GO:0005545 | 1-phosphatidylinositol binding | MF | | 0.0457 |
| GO:0043085 | positive regulation of catalytic activity | BP | | 0.0461 |
| GO:0016903 | oxidoreductase activity, acting on the aldehyde or oxo group of donors | MF | | 0.0468 |
| GO:0032102 | negative regulation of response to external stimulus | BP | | 0.0473 |
| GO:0061333 | renal tubule morphogenesis | BP | | 0.0485 |
| GO:0019005 | SCF ubiquitin ligase complex | CC | | 0.0490 |
| GO:0072332 | intrinsic apoptotic signaling pathway by p53 class mediator | BP | | 0.0495 |
| GO:0090263 | positive regulation of canonical Wnt signaling pathway | BP | | 0.0500 |
| Ranked by P value. GO: Gene Ontology, DMPs: Differentially Methylated Positions; BP: Biological process; MF: Molecular Function; CC: Cellular Component | | | |  |
|  | |  | |  |

| **Supplementary Table S5. The top-ranked CpG sites which show changes in DNA methylation levels between 5-10 years of age in the asthma discordant MZ twins** | | | | | | | | | | | |  |  |  |  |  |
| --- | --- | --- | --- | --- | --- | --- | --- | --- | --- | --- | --- | --- | --- | --- | --- | --- |
| **Probe ID** | **Unaffected Co-Twin Mean** | **Asthma**  **Twin Mean** | **Mean ∆β** | **P value** | **Hg19** | **Relation to CpG Island** | **Gene region feature category (UCSC)** | **SNPs in probe (+/- 10bp SBE)** | **Illumina Gene Annotation** | **Probe Type** | **Gene Annotation from GREAT (Distance from TSS)** | | | | | |
| cg26090469 | -0.0122 | 0.0341 | 0.0463 | 1.41E-06 | Chr5:132271449 |  | Body |  | AFF4 | II | AFF4 (+27904), LEAP2 (+62092) | | | | | |
| cg02181624 | 0.1204 | -0.0649 | -0.1854 | 1.68E-06 | Chr6:168716070 | N_Shelf | Body |  | DACT2 | II | FRMD1 (-236232), DACT2 (+4331) | | | | | |
| cg17199800 | -0.0902 | 0.0620 | 0.1522 | 9.47E-06 | Chr7:96632777 | N_Shore | Body |  | DLX6AS | II | DLX6 (-2512) | | | | | |
| cg21875946 | 0.0892 | -0.0276 | -0.1168 | 1.88E-05 | Chr1:206663985 |  | Body |  | IKBKE | II | RASSF5 (-16893), IKBKE (+20400) | | | | | |
| cg15691003 | 0.0082 | -0.0205 | -0.0287 | 3.26E-05 | Chr1:22351864 | N_Shore | Body;TSS200 |  | HSPC157 | II | CDC42 (-27255), CELA3B (+48447) | | | | | |
| cg02836325 | -0.1285 | -0.0153 | 0.1132 | 3.37E-05 | Chr17:76403955 |  | Body |  | PGS1 | II | PGS1 (+29221), DNAH17 (+169520) | | | | | |
| cg16789707 | -0.0958 | 0.0404 | 0.1362 | 4.33E-05 | Chr16:88767723 | S_Shelf | Body |  | RNF166 | II | SNAI3 (-14842), RNF166 (+5105) | | | | | |
| cg04313071 | 0.1063 | -0.0249 | -0.1312 | 4.33E-05 | Chr14:102685619 |  | Body |  | WDR20 | II | HSP90AA1 (-79534), MOK (+85911) | | | | | |
| cg23847069 | 0.1153 | -0.0057 | -0.1210 | 4.79E-05 | Chr17:2702204 |  | Body |  | RAP1GAP2 | I | RAP1GAP2 (+2473), OR1D5 (+264696) | | | | | |
| cg24293948 | 0.0646 | -0.0729 | -0.1375 | 5.04E-05 | Chr18:61670413 | S_Shore |  |  |  | II | SERPINB8 (+33151), LINC00305 (+95460) | | | | | |
| cg06367321 | 0.1110 | -0.0015 | -0.1125 | 5.14E-05 | Chr16:30125232 |  | TSS200;TSS1500 |  | LOC100271831;GDPD3 | I | GDPD3 (-355) | | | | | |
| cg06655665 | -0.1603 | -0.0359 | 0.1243 | 5.45E-05 | Chr2:38607629 | S_Shelf |  |  |  | II | ATL2 (-3198) | | | | | |
| cg25406011 | 0.1312 | -0.0175 | -0.1487 | 6.04E-05 | Chr11:1254421 | Island | Body |  | MUC5B | II | MUC5B (+10127), TOLLIP (+76470) | | | | | |
| cg25494605 | 0.0873 | -0.0652 | -0.1525 | 6.55E-05 | Chr5:173472097 |  | TSS1500 |  | HMP19 | II | HMP19 (-626) | | | | | |
| cg24664347 | -0.1381 | 0.0087 | 0.1468 | 6.88E-05 | Chr8:86841010 | S_Shore | TSS1500 |  | REXO1L2P | II | REXO1L1 (-51705), PSKH2 (+240840) | | | | | |
| cg18184910 | 0.2047 | 0.0402 | -0.1644 | 7.10E-05 | Chr6:47624247 |  | TSS200 | rs1485784 | GPR111 | II | GPR111 (-78) | | | | | |
| cg00840341 | 0.1094 | 0.0131 | -0.0963 | 7.60E-05 | Chr16:17609453 |  |  |  |  | II | XYLT1 (-44716), AK310228 (+825170) | | | | | |
| cg14141399 | 0.2139 | -0.0312 | -0.2451 | 7.66E-05 | Chr19:52228048 |  | TSS1500 |  | HAS1 | II | HAS1 (-828) | | | | | |
| cg14179581 | -0.1193 | 0.0758 | 0.1951 | 7.74E-05 | Chr1:50881502 | Island |  |  |  | II | DMRTA2 (+7616), ELAVL4 (+306909) | | | | | |
| cg21209859 | -0.0892 | 0.0351 | 0.1242 | 7.78E-05 | Chr1:167682516 | N_Shore |  |  |  | II | CREG1 (-159461), MPZL1 (-8670) | | | | | |
| cg21187770 | 0.1265 | -0.0432 | -0.1697 | 8.57E-05 | Chr2:26205876 | S_Shore | TSS1500 |  | KIF3C | I | KIF3C (-434) | | | | | |
| cg01382502 | -0.1489 | -0.0279 | 0.1210 | 8.96E-05 | Chr16:22252504 |  | Body |  | EEF2K | II | POLR3E (-56236), EEF2K (+34913) | | | | | |
| cg09900436 | -0.1813 | 0.0363 | 0.2175 | 8.96E-05 | Chr20:58630954 |  | TSS200 |  | C20orf197 | II | CDH26 (+97484) | | | | | |
| cg16606561 | 0.2193 | 0.0631 | -0.1562 | 9.72E-05 | Chr20:824641 | N_Shore | 5'UTR;TSS1500 |  | FAM110A | II | FAM110A (+10286), ANGPT4 (+72318) | | | | | |
| cg15048437 | 0.0067 | -0.0159 | -0.0226 | 9.85E-05 | Chr19:37019614 | Island | TSS1500 |  | ZNF260 | I | ZNF260 (-367) | | | | | |
| cg03814957 | 0.0804 | 0.0126 | -0.0678 | 0.0001012 | Chr5:149493099 |  | TSS200 |  | CSF1R | II | CSF1R (-165) | | | | | |
| cg25971649 | 0.0052 | -0.0026 | -0.0078 | 0.000105 | Chr1:117075504 | N_Shore | Body | rs1034919 | CD58 | I | CD58 (+38210), ATP1A1 (+159710) | | | | | |
| cg26280349 | 0.0687 | -0.0060 | -0.0748 | 0.0001079 | Chr22:22011436 | N_Shore |  |  |  | II | PPIL2 (-8836), SDF2L1 (+14895) | | | | | |
| cg01898628 | 0.1824 | -0.0556 | -0.2380 | 0.000108 | Chr17:41836261 | S_Shelf | TSS200 |  | SOST | II | SOST (-106) | | | | | |
| cg16736279 | 0.1436 | 0.0329 | -0.1107 | 0.0001085 | Chr7:3995563 |  | Body |  | SDK1 | II | FOXK1 (-726366), SDK1 (+654484) | | | | | |
| cg26116326 | 0.1312 | -0.0130 | -0.1442 | 0.0001242 | Chr5:173191648 |  |  |  |  | II | BOD1 (-147983), CPEB4 (-123682) | | | | | |
| cg04800320 | 0.0199 | -0.0180 | -0.0379 | 0.0001316 | Chr8:10755899 |  | Body |  | XKR6 | II | SOX7 (-167878), XKR6 (+302975) | | | | | |
| cg18358754 | 0.0069 | -0.0012 | -0.0081 | 0.0001326 | Chr5:154317884 | Island | TSS200 |  | GEMIN5 | I | MRPL22 (-2748), GEMIN5 (-109) | | | | | |
| cg00168634 | -0.1458 | -0.0114 | 0.1344 | 0.0001333 | Chr4:154572830 |  |  |  |  | II | TLR2 (-32610), KIAA0922 (+185333) | | | | | |
| cg12414174 | 0.1114 | -0.0074 | -0.1188 | 0.0001364 | Chr12:89748895 | S_Shore |  |  |  | II | DUSP6 (-2600) | | | | | |
| cg13827287 | -0.0647 | 0.0082 | 0.0729 | 0.0001365 | Chr12:54730461 |  | TSS1500;Body |  | MIR148B;COPZ1 | II | COPZ1 (+11551), GPR84 (+27808) | | | | | |
| cg20727637 | 0.1553 | 0.0166 | -0.1387 | 0.0001376 | Chr4:1762881 | N_Shelf |  |  |  | I | FGFR3 (-32157), TACC3 (+39665) | | | | | |
| cg04602696 | 0.0913 | -0.0747 | -0.1660 | 0.0001489 | Chr16:88846723 | S_Shore | Body |  | FAM38A | II | PIEZO1 (+4648), CTU2 (+73833) | | | | | |
| cg26233253 | 0.1497 | 0.0205 | -0.1292 | 0.0001492 | Chr22:31032613 | S_Shore | 1stExon |  | SLC35E4 | II | SLC35E4 (+821) | | | | | |
| cg10403171 | 0.0618 | 0.0075 | -0.0543 | 0.0001497 | Chr15:91500781 | Island | Body |  | RCCD1 | I | UNC45A (+22567), PRC1 (+37022) | | | | | |
| cg23645885 | 0.0973 | -0.0022 | -0.0995 | 0.0001585 | Chr6:3247958 |  |  |  |  | I | TUBB2B (-19991), SLC22A23 (+208834) | | | | | |
| cg02722672 | -0.0875 | 0.0143 | 0.1018 | 0.0001589 | Chr4:2967106 | S_Shore | Body |  | GRK4 | I | NOP14 (-1989), GRK4 (+1764) | | | | | |
| cg23624705 | -0.0926 | 0.0668 | 0.1594 | 0.0001604 | Chr1:92952655 | S_Shore | TSS1500 |  | GFI1 | II | GFI1 (-1028) | | | | | |
| cg13719901 | 0.1494 | 0.0154 | -0.1339 | 0.0001635 | Chr3:46608139 | S_Shore | 5'UTR;TSS200 |  | LRRC2 | II | TDGF1 (-7905), RTP3 (+68655) | | | | | |
| cg13303203 | 0.0736 | -0.0122 | -0.0858 | 0.0001637 | Chr17:4125596 | S_Shore | Body |  | ANKFY1 | II | ANKFY1 (+41677), CYB5D2 (+79135) | | | | | |
| cg18290981 | 0.1262 | -0.0302 | -0.1564 | 0.0001638 | Chr11:63789520 |  | Body |  | MACROD1 | II | FLRT1 (-81841), OTUB1 (+36196) | | | | | |
| cg18944047 | 0.0000 | 0.0149 | 0.0149 | 0.0001678 | Chr17:72322143 | Island | TSS1500 |  | KIF19 | I | KIF19 (-207) | | | | | |
| cg00158122 | -0.0189 | 0.0652 | 0.0841 | 0.0001692 | Chr10:101290029 | Island |  |  |  | I | NKX2-3 (-2660) | | | | | |
| cg18739537 | -0.0989 | 0.0083 | 0.1072 | 0.0001693 | Chr6:44232189 | N_Shore | Body |  | NFKBIE | II | SLC35B2 (-6907), NFKBIE (+1335) | | | | | |
| cg12828656 | -0.1746 | -0.0384 | 0.1362 | 0.0001807 | Chr5:132577108 |  | Body |  | FSTL4 | II | HSPA4 (+189447), FSTL4 (+371114) | | | | | |
| cg09711814 | 0.0196 | -0.0521 | -0.0716 | 0.0001816 | Chr7:157519166 |  | Body |  | PTPRN2 | II | DNAJB6 (+389457), PTPRN2 (+861315) | | | | | |
| cg26235369 | -0.1197 | -0.0101 | 0.1096 | 0.0001874 | Chr5:173286145 |  |  |  |  | II | BOD1 (-242480), CPEB4 (-29185) | | | | | |
| cg18354462 | 0.1568 | -0.0118 | -0.1686 | 0.0001941 | Chr11:118826751 |  | TSS1500 |  | UPK2 | II | UPK2 (-256) | | | | | |
| cg22513328 | -0.0265 | 0.0112 | 0.0378 | 0.0001965 | Chr7:106267699 |  |  |  |  | II | NAMPT (-342062), PIK3CG (-238224) | | | | | |
| cg19678392 | 0.0668 | -0.0254 | -0.0922 | 0.0002036 | Chr7:94953810 | Island | 1stExon;5'UTR |  | PON1 | I | PON1 (+73) | | | | | |
| cg12354056 | 0.0245 | -0.0153 | -0.0398 | 0.0002059 | Chr3:186136503 |  |  |  |  | II | DGKG (-56481), CRYGS (+125663) | | | | | |
| cg15575009 | 0.0807 | -0.0168 | -0.0975 | 0.0002207 | Chr1:48179471 | S_Shelf |  |  |  | II | FOXD2 (+277783), LOC388630 (+283090) | | | | | |
| cg04432606 | -0.0087 | 0.0181 | 0.0268 | 0.0002223 | Chr2:179315409 | Island | Body;TSS200;1stExon;5'UTR;TSS1500 |  | MIR548N;PRKRA;DFNB59 | II | DFNB59 (-753), PRKRA (+548) | | | | | |
| cg16483426 | -0.0138 | 0.0005 | 0.0142 | 0.0002281 | Chr12:110151678 | Island | TSS1500 |  | C12orf34 | I | TRPV4 (+119533), MVK (+140179) | | | | | |
| cg04185123 | 0.0847 | -0.0092 | -0.0939 | 0.0002356 | Chr5:146812118 |  | Body |  | DPYSL3 | II | DPYSL3 (+77500), STK32A (+197540) | | | | | |
| cg19674797 | 0.0975 | -0.0391 | -0.1366 | 0.0002363 | Chr20:60886077 | Island | Body |  | LAMA5 | I | ADRM1 (+8051), LAMA5 (+56290) | | | | | |
| cg03675083 | -0.0806 | 0.0475 | 0.1281 | 0.0002374 | Chr3:194064806 |  | 5'UTR |  | CPN2 | II | CPN2 (+7250), HES1 (+210876) | | | | | |
| cg06748511 | 0.0232 | -0.0056 | -0.0288 | 0.0002378 | Chr16:67693572 | Island | TSS1500;Body |  | PARD6A;ACD | I | PARD6A (-1278), ACD (+1145) | | | | | |
| cg24585210 | 0.1325 | 0.0213 | -0.1112 | 0.000239 | Chr3:107597020 |  | TSS200 |  | LOC151658 | II | CD47 (+212914), BBX (+355238) | | | | | |
| cg06058681 | -0.0788 | 0.0235 | 0.1023 | 0.000239 | Chr1:38757813 |  |  |  |  | I | POU3F1 (-245364), RRAGC (+567526) | | | | | |
| cg19440035 | 0.1324 | 0.0098 | -0.1227 | 0.0002412 | Chr19:6333701 |  | TSS200 |  | ACER1 | II | ACER1 (-62) | | | | | |
| cg04609640 | 0.2185 | -0.0745 | -0.2930 | 0.0002455 | Chr1:45254092 | S_Shore | TSS1500 |  | BEST4 | II | BEST4 (-667) | | | | | |
| cg00188654 | 0.1341 | -0.0016 | -0.1357 | 0.0002475 | Chr16:3696402 |  |  |  |  | II | SLX4 (-34818), DNASE1 (-6537) | | | | | |
| cg08732659 | -0.1503 | -0.0381 | 0.1121 | 0.0002508 | Chr16:54973966 | S_Shore |  |  |  | I | IRX6 (-384504), IRX5 (+8856) | | | | | |
| cg19547319 | -0.0565 | 0.0380 | 0.0944 | 0.0002518 | Chr10:102987993 | Island | Body;TSS1500 |  | LBX1;FLJ41350 | I | LBX1 (+723) | | | | | |
| cg07762181 | -0.0693 | 0.0090 | 0.0782 | 0.0002575 | Chr4:56660143 | Island |  |  |  | II | NMU (-157679), EXOC1 (-59672) | | | | | |
| cg18846804 | 0.0340 | -0.0669 | -0.1009 | 0.0002584 | Chr19:55966376 | S_Shelf | Body |  | ISOC2 | II | SHISA7 (-12147), ISOC2 (+6672) | | | | | |
| cg10048215 | -0.0604 | 0.0440 | 0.1043 | 0.0002753 | Chr12:12938383 | N_Shore | Body;TSS200 |  | APOLD1 | II | DDX47 (-27757), APOLD1 (+59533) | | | | | |
| cg03398481 | 0.1090 | 0.0240 | -0.0851 | 0.0002754 | Chr15:81666671 |  | TSS1500 |  | TMC3 | II | TMC3 (-254) | | | | | |
| cg13926833 | 0.1149 | 0.0146 | -0.1003 | 0.0002765 | Chr6:109274429 |  | Body |  | ARMC2 | I | ARMC2 (+104811), SESN1 (+141278) | | | | | |
| cg23351271 | 0.0916 | 0.0068 | -0.0848 | 0.000282 | Chr8:145644123 | S_Shelf |  |  |  | II | SLC39A4 (-1851) | | | | | |
| cg01280841 | -0.0772 | 0.0312 | 0.1083 | 0.0002943 | Chr7:100181701 | N_Shore | Body |  | LRCH4 | II | SAP25 (-10432), LRCH4 (+2074) | | | | | |
| cg23337845 | 0.0591 | 0.0031 | -0.0560 | 0.0002945 | Chr4:170641374 |  | 3'UTR |  | CLCN3 | II | C4orf27 (+37718), CLCN3 (+99703) | | | | | |
| cg21618455 | 0.0662 | -0.0519 | -0.1181 | 0.0002949 | Chr3:123950018 |  | Body |  | KALRN | II | UMPS (-499194), KALRN (+136461) | | | | | |
| cg23654219 | 0.0918 | -0.0314 | -0.1232 | 0.0003006 | Chr6:31127527 | S_Shore | Body |  | TCF19 | II | CCHCR1 (-1962), TCF19 (+1225) | | | | | |
| cg09969018 | 0.0088 | -0.0198 | -0.0286 | 0.0003012 | Chr8:30891709 |  | TSS1500;5'UTR |  | PURG;WRN | I | PURG (-1393), WRN (+932) | | | | | |
| cg21471199 | -0.1418 | 0.0585 | 0.2003 | 0.0003016 | Chr1:19218212 |  | TSS1500;Body |  | ALDH4A1 | II | TAS1R2 (-32058), ALDH4A1 (+11080) | | | | | |
| cg09292226 | -0.1083 | -0.0099 | 0.0984 | 0.0003029 | Chr6:1392250 | N_Shore | Body |  | FOXF2 | II | FOXC1 (-218430), FOXF2 (+2182) | | | | | |
| cg01741626 | -0.0969 | 0.0652 | 0.1621 | 0.0003032 | Chr16:4364093 | Island |  |  |  | I | TFAP4 (-41093), GLIS2 (-18122) | | | | | |
| cg24866491 | -0.0265 | 0.0092 | 0.0357 | 0.000304 | Chr7:108210991 | S_Shore | TSS1500;5'UTR |  | THAP5;DNAJB9 | II | THAP5 (-825), DNAJB9 (+803) | | | | | |
| cg16208959 | -0.0550 | 0.0456 | 0.1006 | 0.0003059 | Chr3:128296058 |  | TSS1500 |  | C3orf27 | II | GATA2 (-84029), RPN1 (+73660) | | | | | |
| cg11039913 | -0.0514 | 0.0381 | 0.0895 | 0.0003064 | Chr6:31024501 |  | Body | rs116322068 | HCG22 | II | MUC22 (+50773), C6orf15 (+55830) | | | | | |
| cg23603194 | 0.1023 | -0.0543 | -0.1566 | 0.0003105 | Chr1:221060753 | N_Shore |  |  |  | II | HLX (+8011), DUSP10 (+854762) | | | | | |
| cg09944027 | 0.0592 | -0.0682 | -0.1274 | 0.0003155 | Chr18:60264247 | Island |  |  |  | II | PHLPP1 (-118424), ZCCHC2 (+73590) | | | | | |
| cg19111712 | 0.0149 | -0.0165 | -0.0314 | 0.00032 | Chr11:1301065 | N_Shore | Body |  | TOLLIP | I | TOLLIP (+29826), MUC5B (+56771) | | | | | |
| cg13540981 | -0.0353 | 0.0832 | 0.1186 | 0.000331 | Chr6:32011506 |  | Body |  | TNXB | II | CYP21A2 (+5414), TNXB (+65644) | | | | | |
| cg07688779 | -0.0072 | 0.0172 | 0.0244 | 0.0003311 | Chr15:37391207 | N_Shore | 1stExon;Body;5'UTR |  | MEIS2 | I | MEIS2 (+1533) | | | | | |
| cg10681065 | -0.1543 | 0.0067 | 0.1610 | 0.0003329 | Chr7:100239172 |  | 5'UTR;1stExon |  | TFR2 | II | TFR2 (+28) | | | | | |
| cg16233074 | 0.2057 | -0.0091 | -0.2148 | 0.0003362 | Chr17:42445042 | S_Shelf |  |  |  | II | FAM171A2 (-3808) | | | | | |
| cg13614229 | 0.0360 | -0.0567 | -0.0927 | 0.0003373 | Chr12:131217218 |  |  |  |  | I | RIMBP2 (-214757), STX2 (+106592) | | | | | |
| cg08141959 | 0.1167 | 0.0357 | -0.0810 | 0.0003377 | Chr13:106809125 |  |  |  |  | II | EFNB2 (+378262), DAOA (+690561) | | | | | |
| cg11668844 | -0.0263 | 0.0331 | 0.0594 | 0.0003434 | Chr13:113655622 | N_Shore | Body |  | MCF2L | II | F7 (-104482), MCF2L (+32088) | | | | | |
| cg15810415 | 0.1436 | 0.0262 | -0.1174 | 0.0003439 | Chr4:111397134 | N_Shore | TSS200 |  | ENPEP | II | ENPEP (-94) | | | | | |
| cg00035268 | 0.0342 | -0.0403 | -0.0745 | 0.0003465 | Chr6:168225394 | N_Shore | Body |  | C6orf124 | II | MLLT4 (-2276) | | | | | |
| cg09300114 | -0.0161 | 0.0196 | 0.0357 | 0.0003493 | Chr17:73084329 | Island | 5'UTR |  | SLC16A5 | II | SLC16A5 (+275) | | | | | |
| cg11681597 | -0.1485 | -0.0102 | 0.1383 | 0.0003495 | Chr1:249116587 | N_Shelf | Body |  | SH3BP5L | II | LOC646627 (-213437), ZNF672 (-15942) | | | | | |
| cg00585790 | -0.1563 | -0.0475 | 0.1088 | 0.0003506 | Chr2:109204845 |  | 1stExon;5'UTR |  | LIMS1 | II | LIMS1 (-66422), GCC2 (+139269) | | | | | |
| cg18720687 | -0.0528 | 0.0031 | 0.0559 | 0.0003507 | Chr17:855312 |  | Body |  | NXN | II | GLOD4 (-169742), NXN (+27685) | | | | | |
| cg18780259 | -0.0377 | 0.0237 | 0.0615 | 0.0003536 | Chr19:19006582 | Island | 5'UTR;Body |  | GDF1;LASS1 | I | CERS1 (+370) | | | | | |
| cg16606638 | -0.0316 | 0.0364 | 0.0680 | 0.0003581 | Chr12:89918221 | N_Shore | Body;1stExon |  | WDR51B;GALNT4 | II | GALNT4 (+361) | | | | | |
| cg04365973 | 0.0800 | -0.0001 | -0.0801 | 0.0003629 | Chr16:717556 | N_Shore | TSS1500;Body |  | RHOT2;WDR90 | I | RHOT2 (-526) | | | | | |
| cg05812089 | -0.1359 | -0.0070 | 0.1289 | 0.000363 | Chr11:19736648 | S_Shore | TSS1500;Body |  | LOC100126784;NAV2 | II | NAV2 (+1768), DBX1 (+445221) | | | | | |
| cg20940153 | -0.0239 | 0.0028 | 0.0267 | 0.0003691 | Chr8:11059066 | Island | TSS200 |  | XKR6 | II | XKR6 (-192) | | | | | |
| cg24708332 | -0.0140 | 0.0305 | 0.0445 | 0.0003724 | Chr2:102794246 |  | 3'UTR |  | IL1R1 | II | IL1RL2 (-9186), IL1R1 (+23846) | | | | | |
| cg24046475 | -0.1372 | 0.0102 | 0.1474 | 0.0003784 | Chr8:86371819 | N_Shelf |  |  |  | II | CA2 (-4311) | | | | | |
| cg02998936 | -0.0232 | 0.0134 | 0.0366 | 0.0003797 | Chr17:76338383 | Island |  |  |  | II | SOCS3 (+17774), TMEM235 (+110993) | | | | | |
| cg06897927 | -0.0206 | 0.0050 | 0.0256 | 0.000384 | Chr10:124895500 | Island | TSS200 |  | HMX3 | I | HMX3 (-66) | | | | | |
| cg16075139 | 0.0737 | 0.0134 | -0.0603 | 0.0003845 | Chr11:2406923 | Island | Body |  | CD81 | I | CD81 (+8377), TRPM5 (+37351) | | | | | |
| cg09940915 | 0.1121 | -0.0819 | -0.1939 | 0.0003846 | Chr1:22925447 | N_Shore | Body |  | EPHA8 | II | C1QA (-37670), EPHA8 (+35444) | | | | | |
| cg03145274 | -0.0688 | 0.0476 | 0.1164 | 0.0003883 | Chr1:8383899 | N_Shore | TSS1500 |  | SLC45A1 | I | SLC45A1 (-490) | | | | | |
| cg19523819 | 0.0909 | 0.0157 | -0.0752 | 0.0003901 | Chr8:125741259 | Island | TSS1500 |  | MTSS1 | II | MTSS1 (-530) | | | | | |
| cg16444641 | 0.1892 | -0.0608 | -0.2500 | 0.0003931 | Chr2:10567628 | S_Shore | 3'UTR |  | HPCAL1 | II | ODC1 (+20824), HPCAL1 (+124589) | | | | | |
| cg07946458 | -0.0312 | 0.0014 | 0.0326 | 0.0003932 | Chr21:38338892 | Island | 5'UTR |  | HLCS | II | HLCS (+14371), SIM2 (+266902) | | | | | |
| cg17861161 | 0.0915 | -0.0245 | -0.1160 | 0.0003978 | Chr1:3184434 | N_Shore | Body |  | PRDM16 | II | ARHGEF16 (-186712), PRDM16 (+198693) | | | | | |
| cg13487992 | -0.0571 | 0.0104 | 0.0675 | 0.000401 | Chr14:23511078 |  | TSS1500 |  | PSMB11 | II | PSMB11 (-297) | | | | | |
| cg27470307 | 0.0211 | -0.0081 | -0.0293 | 0.0004022 | Chr11:61106785 | Island | Body |  | DAK | I | DDB1 (-6120), CYBASC3 (+17575) | | | | | |
| cg07937443 | -0.0713 | 0.0281 | 0.0994 | 0.0004056 | Chr4:174442543 | N_Shore |  |  |  | II | SCRG1 (-121927), HAND2 (+8834) | | | | | |
| cg19903196 | -0.1970 | -0.0424 | 0.1545 | 0.0004088 | Chr1:212429231 |  |  |  |  | II | INTS7 (-220230), PPP2R5A (-29647) | | | | | |
| cg24432632 | -0.1123 | 0.0440 | 0.1564 | 0.0004146 | Chr10:3935314 |  |  |  |  | II | AKR1E2 (-933087), KLF6 (-107842) | | | | | |
| cg26209676 | 0.1351 | 0.0225 | -0.1126 | 0.0004225 | Chr19:56154382 | Island | TSS1500;1stExon;Body |  | ZNF581 | II | CCDC106 (-4571), ZNF581 (-603) | | | | | |
| cg02831604 | -0.1074 | 0.0384 | 0.1458 | 0.0004229 | Chr15:76628725 | N_Shore | TSS1500 |  | ISL2 | II | ISL2 (-421) | | | | | |
| cg02850003 | -0.0086 | 0.0023 | 0.0109 | 0.0004261 | Chr14:60558592 | Island | TSS200 |  | C14orf135 | I | C14orf135 (-36) | | | | | |
| cg23511157 | 0.1507 | 0.0137 | -0.1369 | 0.0004274 | Chr7:123241752 |  |  |  |  | II | NDUFA5 (-43795), ASB15 (-7359) | | | | | |
| cg24483352 | 0.0196 | -0.0137 | -0.0333 | 0.0004314 | Chr8:911188 | N_Shelf |  |  |  | II | DLGAP2 (-538380), C8orf42 (-415858) | | | | | |
| cg08107075 | 0.0430 | -0.0275 | -0.0705 | 0.0004314 | Chr7:24606883 |  |  |  |  | II | MPP6 (-6201), NPY (+283077) | | | | | |
| cg21117699 | -0.0650 | 0.0552 | 0.1202 | 0.0004315 | Chr7:31474609 |  |  |  |  | II | PPP1R17 (-252021), NEUROD6 (-94072) | | | | | |
| cg26058474 | 0.1279 | -0.0180 | -0.1459 | 0.0004337 | Chr2:241975035 | Island | Body |  | SNED1 | I | SNED1 (+36781), PASK (+113883) | | | | | |
| cg06259881 | 0.0048 | -0.0187 | -0.0235 | 0.0004446 | Chr16:54321165 | Island | TSS1500 |  | IRX3 | I | IRX3 (-788) | | | | | |
| cg02148324 | 0.1035 | -0.0183 | -0.1218 | 0.0004486 | Chr7:150925493 | S_Shore | TSS1500 |  | ABCF2 | II | CHPF2 (-4091), ABCF2 (-1177) | | | | | |
| cg14777519 | -0.0788 | 0.0404 | 0.1191 | 0.0004516 | Chr3:47890012 | N_Shore | Body;TSS1500 |  | DHX30;MIR1226 | II | DHX30 (+45614), MAP4 (+240756) | | | | | |
| cg07821676 | 0.0723 | -0.0954 | -0.1676 | 0.0004579 | Chr12:124945965 |  | Body |  | NCOR2 | II | NCOR2 (+106044), ZNF664 (+488204) | | | | | |
| cg04446653 | -0.1229 | 0.0064 | 0.1292 | 0.0004586 | Chr3:181500537 |  |  |  |  | II | SOX2 (+70826) | | | | | |
| cg00429081 | -0.0073 | 0.0105 | 0.0179 | 0.0004667 | Chr19:10764351 | Island | TSS1500;Body |  | ILF3;LOC147727 | I | ILF3 (-585) | | | | | |
| cg00116699 | -0.1035 | 0.0170 | 0.1205 | 0.0004684 | Chr2:240205260 |  | Body |  | HDAC4 | II | HDAC4 (+117382), TWIST2 (+448588) | | | | | |
| cg18250832 | 0.0846 | -0.0103 | -0.0949 | 0.0004693 | Chr2:232395463 | S_Shore | TSS1500 |  | NMUR1 | II | NMUR1 (-282) | | | | | |
| cg12208691 | 0.1618 | 0.0137 | -0.1481 | 0.0004723 | Chr1:193052736 |  | Body |  | TROVE2 | II | UCHL5 (-23548), GLRX2 (+21871) | | | | | |
| cg04408162 | 0.0794 | 0.0169 | -0.0624 | 0.0004805 | Chr8:140643009 |  | Body |  | KCNK9 | II | COL22A1 (-716774), KCNK9 (+72289) | | | | | |
| cg15553814 | -0.0310 | 0.0625 | 0.0935 | 0.000482 | Chr1:58450472 |  | 5'UTR |  | DAB1 | II | DAB1 (+265738) | | | | | |
| cg01507173 | 0.1471 | 0.0208 | -0.1264 | 0.0004885 | Chr2:113816662 |  | 5'UTR;TSS200 |  | IL1F5 | II | IL36RN (-22) | | | | | |
| cg22320035 | -0.0245 | 0.0368 | 0.0613 | 0.0004938 | Chr22:20764816 | S_Shelf |  |  |  | II | ZNF74 (+16337), SCARF2 (+27329) | | | | | |
| cg01484915 | -0.1322 | -0.0037 | 0.1286 | 0.000497 | Chr10:45458834 | S_Shelf | 5'UTR |  | RASSF4 | II | RASSF4 (+3616), C10orf10 (+15495) | | | | | |
| cg26120971 | -0.0598 | 0.0042 | 0.0640 | 0.0004988 | Chr8:118676025 |  |  |  |  | II | MED30 (+143061), EXT1 (+448032) | | | | | |
| cg25022327 | 0.1637 | -0.0060 | -0.1697 | 0.0005012 | Chr11:128812462 |  | 5'UTR |  | TP53AIP1 | II | TP53AIP1 (-4674) | | | | | |
| cg11977326 | -0.0082 | 0.0035 | 0.0116 | 0.0005031 | Chr1:44820970 | Island | TSS200 |  | ERI3 | I | ERI3 (-32) | | | | | |
| cg13872831 | 0.0130 | -0.0003 | -0.0133 | 0.0005057 | Chr3:50377988 | Island | 1stExon |  | RASSF1 | I | RASSF1 (+378) | | | | | |
| cg18063493 | 0.0529 | -0.0080 | -0.0609 | 0.0005059 | Chr17:43196221 | N_Shelf | Body |  | PLCD3 | II | PLCD3 (+13669), NMT1 (+57542) | | | | | |
| cg11814446 | -0.0778 | 0.0335 | 0.1113 | 0.0005061 | Chr12:65562996 | N_Shore | TSS1500 |  | LEMD3 | II | LEMD3 (-354) | | | | | |
| cg15444478 | 0.0865 | -0.0134 | -0.0999 | 0.0005082 | Chr12:130494697 |  |  |  |  | I | FZD10 (-152306), TMEM132D (-106486) | | | | | |
| cg01512089 | -0.1207 | 0.0288 | 0.1495 | 0.000512 | Chr17:41705043 |  |  |  |  | II | ETV4 (-81739), MEOX1 (+33887) | | | | | |
| cg08935613 | 0.0082 | -0.0441 | -0.0523 | 0.0005156 | Chr11:125753056 | N_Shelf | TSS1500 | rs73626609 | HYLS1 | II | HYLS1 (-452) | | | | | |
| cg18675610 | 0.0810 | -0.0020 | -0.0830 | 0.0005162 | Chr10:32216311 | N_Shore | 5'UTR |  | ARHGAP12 | I | ARHGAP12 (+1458), ZEB1 (+608211) | | | | | |
| cg03802272 | -0.0464 | 0.0271 | 0.0735 | 0.0005192 | Chr3:158361750 | N_Shore | TSS1500 |  | GFM1 | II | GFM1 (-566) | | | | | |
| cg24286220 | 0.0950 | -0.0008 | -0.0958 | 0.0005207 | Chr11:94290258 |  |  |  |  | II | PIWIL4 (-10215), FUT4 (+13242) | | | | | |
| cg09230442 | -0.0457 | 0.0017 | 0.0474 | 0.0005287 | Chr15:65809997 | Island | 1stExon;1stExon;5'UTR |  | DPP8 | I | DPP8 (+37) | | | | | |
| cg00953006 | 0.0894 | -0.0319 | -0.1213 | 0.0005293 | Chr16:633868 | Island | 3'UTR |  | PIGQ | II | RAB40C (-6208), PIGQ (+13901) | | | | | |
| cg19354656 | 0.1228 | -0.0013 | -0.1242 | 0.0005334 | Chr1:159916472 | S_Shore | TSS1500 |  | IGSF9 | II | IGSF9 (-1087) | | | | | |
| cg01697477 | -0.1127 | -0.0135 | 0.0992 | 0.0005451 | Chr3:25614606 |  | Body | rs1286739 | RARB | II | TOP2B (+91224), RARB (+144773) | | | | | |
| cg00162064 | -0.1463 | -0.0093 | 0.1369 | 0.0005468 | Chr1:44998151 |  | Body |  | RNF220 | II | RNF220 (+127192), TMEM53 (+141947) | | | | | |
| cg24845329 | 0.1300 | -0.0046 | -0.1346 | 0.0005562 | Chr19:376532 | S_Shore | TSS1500 |  | THEG | II | THEG (-520) | | | | | |
| cg08655330 | 0.0303 | -0.0681 | -0.0984 | 0.0005574 | Chr1:19132135 |  |  |  |  | II | TAS1R2 (+54019), PAX7 (+174636) | | | | | |
| cg01693026 | -0.0053 | 0.0050 | 0.0103 | 0.0005624 | Chr14:102026953 | Island | TSS1500;TSS200 |  | DIO3;MIR1247 | I | DIO3 (-734) | | | | | |
| cg07726288 | 0.0473 | -0.0030 | -0.0503 | 0.0005745 | Chr1:248855278 | Island |  |  |  | II | OR14I1 (-9674), LOC646627 (+47872) | | | | | |
| cg22486621 | 0.0345 | -0.0244 | -0.0589 | 0.0005752 | Chr12:2049079 |  |  |  |  | I | CACNA2D4 (-21210), DCP1B (+64597) | | | | | |
| cg21784917 | -0.0256 | 0.0204 | 0.0460 | 0.0005803 | Chr7:79084038 | S_Shore | TSS1500 |  | MAGI2 | I | MAGI2 (-1149) | | | | | |
| cg12211867 | -0.0748 | 0.0227 | 0.0976 | 0.0005845 | Chr1:108023441 | Island | Body |  | NTNG1 | II | NTNG1 (+340813), VAV3 (+484103) | | | | | |
| cg04638150 | 0.1123 | -0.0191 | -0.1314 | 0.000587 | Chr11:62273418 |  | Body |  | AHNAK | I | AHNAK (+40913), SCGB1A1 (+86912) | | | | | |
| cg24275315 | -0.0171 | 0.0532 | 0.0702 | 0.0005882 | Chr4:85417312 | N_Shore | Body |  | NKX6-1 | II | NKX6-1 (+2074), AGPAT9 (+959656) | | | | | |
| cg14149663 | -0.0447 | 0.0274 | 0.0722 | 0.0005882 | Chr9:72371073 | N_Shelf | Body |  | PTAR1 | II | APBA1 (-83799), PTAR1 (+3802) | | | | | |
| cg04506514 | 0.0245 | -0.0434 | -0.0678 | 0.0006029 | Chr2:220254248 | S_Shore |  |  |  | II | DNPEP (-1587) | | | | | |
| cg19377754 | -0.0018 | 0.0297 | 0.0315 | 0.0006066 | Chr3:71633994 | S_Shore | TSS1500 |  | FOXP1 | II | FOXP1 (-339679), EIF4E3 (+140531) | | | | | |
| cg08695418 | 0.0618 | -0.0343 | -0.0961 | 0.0006081 | Chr4:6729199 | Island |  |  |  | II | KIAA0232 (-55259), CNO (+11358) | | | | | |
| cg16928046 | -0.0493 | 0.0245 | 0.0738 | 0.0006114 | Chr17:45812243 | S_Shore | Body |  | TBX21 | II | TBX21 (+1634), OSBPL7 (+86903) | | | | | |
| cg10579986 | 0.0093 | 0.0440 | 0.0347 | 0.0006129 | Chr3:197401451 |  | TSS200;3'UTR;3'UTR |  | MIR922;KIAA0226 | II | BDH1 (-118594), KIAA0226 (+62321) | | | | | |
| cg22751627 | 0.0313 | -0.0482 | -0.0795 | 0.0006153 | Chr5:56718310 |  |  |  |  | I | ACTBL2 (+60325), GPBP1 (+208410) | | | | | |
| cg02872693 | 0.1391 | -0.0330 | -0.1721 | 0.0006245 | Chr20:18295599 |  | Body |  | ZNF133 | II | ZNF133 (+26479), DZANK1 (+152229) | | | | | |
| cg09928027 | -0.0225 | 0.0423 | 0.0648 | 0.0006549 | Chr16:88945708 | Island | Body |  | CBFA2T3 | I | PABPN1L (-12641), CBFA2T3 (+97795) | | | | | |
| cg12644072 | -0.0493 | 0.0314 | 0.0807 | 0.000656 | Chr1:118438345 |  | Body |  | GDAP2 | II | GDAP2 (+33956), MAN1A2 (+528261) | | | | | |
| cg15596666 | 0.0815 | -0.0437 | -0.1252 | 0.0006648 | Chr10:135160792 |  | TSS200 |  | PRAP1 | II | CALY (-10318), C10orf125 (+10736) | | | | | |
| cg11635053 | 0.1723 | 0.0129 | -0.1595 | 0.0006652 | Chr3:184055217 | N_Shore | 5'UTR;TSS200 |  | FAM131A | II | FAM131A (-58) | | | | | |
| cg06463800 | -0.1045 | 0.0419 | 0.1464 | 0.0006673 | Chr13:25754183 |  |  |  |  | II | PABPC3 (+83908), MTMR6 (+107520) | | | | | |
| cg07451370 | -0.0751 | 0.0298 | 0.1049 | 0.0006783 | Chr11:126848024 |  | Body |  | KIRREL3 | II | KIRREL3 (+22741), ST3GAL4 (+622485) | | | | | |
| cg01305427 | 0.0166 | -0.0455 | -0.0621 | 0.0006787 | Chr10:99397203 | N_Shelf |  |  |  | II | PI4K2A (-3239) | | | | | |
| cg00974864 | -0.1658 | -0.0179 | 0.1480 | 0.0006797 | Chr1:161601053 |  | 5'UTR;1stExon |  | FCGR3B | II | FCGR3B (+198) | | | | | |
| cg08641445 | 0.1149 | 0.0046 | -0.1102 | 0.0006814 | Chr16:1080637 | Island |  |  |  | II | SSTR5 (-42118), SOX8 (+48830) | | | | | |
| cg22521129 | -0.0531 | 0.0230 | 0.0761 | 0.0006831 | Chr5:1345308 | Island | TSS1500 |  | CLPTM1L | II | CLPTM1L (-307) | | | | | |
| cg16776035 | -0.0021 | -0.1218 | -0.1197 | 0.0006835 | Chr17:74542680 |  |  |  |  | II | PRCD (+6560), ST6GALNAC2 (+39464) | | | | | |
| cg26276650 | -0.0220 | 0.0336 | 0.0556 | 0.0006859 | Chr2:10182878 | Island | TSS1500 |  | KLF11 | I | KLF11 (-803) | | | | | |
| cg27162882 | -0.1383 | -0.0204 | 0.1180 | 0.0006874 | Chr7:27258886 | N_Shore |  |  |  | II | EVX1 (-23277), HOXA13 (-19162) | | | | | |
| cg24295658 | 0.0124 | -0.0413 | -0.0537 | 0.0006955 | Chr19:7285146 |  | Body |  | INSR | II | INSR (+8864), ZNF557 (+215676) | | | | | |
| cg26937778 | 0.1552 | 0.0050 | -0.1502 | 0.0006966 | Chr14:96562667 |  |  |  |  | II | BDKRB2 (-108467), C14orf132 (+57007) | | | | | |
| cg13490352 | -0.1275 | 0.0394 | 0.1669 | 0.0006968 | Chr7:55072042 | N_Shore |  |  |  | I | SEC61G (-245104), EGFR (-14682) | | | | | |
| cg13871708 | -0.0800 | 0.0500 | 0.1300 | 0.0007004 | Chr6:30921007 | N_Shelf | 3'UTR |  | DPCR1 | II | MUC21 (-30477), DPCR1 (+12231) | | | | | |
| cg11330740 | -0.0527 | 0.0073 | 0.0601 | 0.0007019 | Chr5:172660884 | Island | Body |  | NKX2-5 | II | NKX2-5 (+1430), BNIP1 (+89440) | | | | | |
| cg03591753 | -0.0602 | -0.0089 | 0.0513 | 0.0007021 | Chr6:35659141 | S_Shelf | 5'UTR |  | FKBP5 | II | FKBP5 (-2423) | | | | | |
| cg24556382 | 0.1160 | 0.0005 | -0.1155 | 0.0007167 | Chr4:174173455 |  | Body |  | GALNT7 | II | HMGB2 (+81464), GALNT7 (+83552) | | | | | |
| cg05522011 | 0.1367 | 0.0233 | -0.1134 | 0.0007181 | Chr4:81122726 | N_Shore | Body |  | PRDM8 | I | FGF5 (-65015), PRDM8 (+4070) | | | | | |
| cg14862981 | 0.0282 | -0.0144 | -0.0426 | 0.0007221 | Chr5:180617100 | Island |  |  |  | I | TRIM7 (+15076), OR2V2 (+35158) | | | | | |
| cg22877024 | 0.2061 | 0.0696 | -0.1365 | 0.0007264 | Chr5:143021669 |  |  |  |  | II | NR3C1 (-237625), YIPF5 (+528608) | | | | | |
| cg14319569 | 0.0515 | -0.0324 | -0.0839 | 0.0007268 | Chr19:41769282 | Island | TSS1500;5'UTR |  | HNRNPUL1 | I | HNRNPUL1 (-837) | | | | | |
| cg02147681 | 0.1209 | -0.0595 | -0.1803 | 0.0007315 | Chr17:17603837 | Island | 5'UTR |  | RAI1 | I | RAI1 (+19051), SREBF1 (+136487) | | | | | |
| cg02479986 | -0.0746 | 0.0277 | 0.1023 | 0.0007455 | Chr1:203027445 |  | Body |  | PPFIA4 | II | PPFIA4 (+23834), MYOG (+27931) | | | | | |
| cg17435965 | -0.0327 | 0.0719 | 0.1047 | 0.0007458 | Chr17:79278193 |  | 3'UTR |  | C17orf55 | II | SLC38A10 (-9098), TMEM105 (+26280) | | | | | |
| cg06419476 | -0.0774 | 0.0024 | 0.0798 | 0.000754 | Chr19:42636978 | N_Shore | TSS1500 |  | POU2F2 | II | POU2F2 (-354) | | | | | |
| cg12145289 | 0.0174 | -0.0156 | -0.0330 | 0.0007599 | Chr11:65385911 | S_Shelf | Body |  | PCNXL3 | I | MAP3K11 (-4192), PCNXL3 (+2129) | | | | | |
| cg12692682 | 0.1138 | 0.0165 | -0.0973 | 0.00076 | Chr1:212202711 |  | Body |  | INTS7 | II | LPGAT1 (-198598), DTL (-6207) | | | | | |
| cg25185881 | -0.0298 | 0.0051 | 0.0349 | 0.0007629 | Chr4:156681047 | Island | Body |  | GUCY1B3 | II | GUCY1B3 (+922) | | | | | |
| cg08110337 | 0.0626 | -0.0315 | -0.0941 | 0.0007868 | Chr2:39493603 |  | Body |  | MAP4K3 | II | CDKL4 (-36931), MAP4K3 (+170615) | | | | | |
| cg09362366 | 0.0887 | -0.0020 | -0.0908 | 0.0007869 | Chr3:159710706 | S_Shelf | Body |  | IL12A | II | C3orf80 (-232716), IL12A (+4084) | | | | | |
| cg16377880 | 0.1405 | 0.0191 | -0.1215 | 0.0008045 | Chr19:15751167 |  | TSS1500 |  | CYP4F3 | II | CYP4F3 (-539) | | | | | |
| cg02330507 | 0.1642 | 0.0370 | -0.1272 | 0.0008062 | Chr12:56333313 | S_Shelf | Body |  | DGKA | II | DGKA (+7502), PMEL (+27182) | | | | | |
| cg08946601 | -0.1037 | -0.0087 | 0.0950 | 0.0008106 | Chr6:108489064 | Island | Body |  | NR2E1 | II | NR2E1 (+1850), SNX3 (+93399) | | | | | |
| cg02057917 | 0.0644 | -0.0103 | -0.0747 | 0.0008137 | Chr6:29323269 |  | 1stExon |  | OR5V1 | II | OR5V1 (+784) | | | | | |
| cg07134316 | 0.1653 | 0.0055 | -0.1598 | 0.0008183 | Chr12:57488112 |  | Body |  | NAB2 | I | NAB2 (+5436), STAT6 (+17083) | | | | | |
| cg12177793 | -0.0553 | 0.0285 | 0.0838 | 0.0008238 | Chr14:24837892 | Island | Body |  | NFATC4 | I | NYNRIN (-30099), NFATC4 (+1748) | | | | | |
| cg02246002 | 0.1136 | -0.0301 | -0.1437 | 0.0008245 | Chr13:37101457 |  |  |  |  | II | SERTM1 (-146591), CCNA1 (+95049) | | | | | |
| cg00460983 | -0.0713 | 0.0330 | 0.1043 | 0.0008348 | Chr10:91133941 |  |  |  |  | II | IFIT1B (-3871) | | | | | |
| cg18528696 | 0.1531 | 0.0530 | -0.1000 | 0.0008355 | Chr12:64925934 |  |  |  |  | I | RASSF3 (-78358), TBK1 (+80095) | | | | | |
| cg26856369 | -0.0241 | 0.0382 | 0.0622 | 0.0008386 | Chr5:150324958 | N_Shore | Body |  | LOC134466 | II | ZNF300P1 (-1420) | | | | | |
| cg05645982 | -0.1327 | -0.0225 | 0.1102 | 0.0008406 | Chr8:124219077 | N_Shore | Body |  | FAM83A | II | C8orf76 (+34560), WDR67 (+134158) | | | | | |
| cg05537387 | 0.1207 | 0.0515 | -0.0692 | 0.0008438 | Chr7:55174999 |  | Body |  | EGFR | II | LANCL2 (-258141), EGFR (+88275) | | | | | |
| cg21606036 | 0.1153 | 0.0136 | -0.1017 | 0.0008565 | Chr5:78985900 |  | Body |  | CMYA5 | II | CMYA5 (+242) | | | | | |
| cg08747717 | -0.0446 | 0.0421 | 0.0866 | 0.0008575 | Chr16:50186775 |  | TSS200 |  | PAPD5 | I | PAPD5 (-53) | | | | | |
| cg01780164 | -0.1376 | -0.0149 | 0.1228 | 0.0008608 | Chr14:70343734 | N_Shelf |  |  |  | II | SMOC1 (-2379) | | | | | |
| cg12570419 | 0.0697 | -0.0960 | -0.1657 | 0.0008617 | Chr9:138636968 | Island | Body |  | KCNT1 | II | KCNT1 (+42938), CAMSAP1 (+162036) | | | | | |
| cg10628098 | -0.0368 | 0.0191 | 0.0559 | 0.0008689 | Chr11:65292300 | N_Shore | TSS1500 |  | SCYL1 | II | SCYL1 (-247) | | | | | |
| cg21518208 | -0.0384 | 0.0335 | 0.0719 | 0.000872 | Chr12:52761416 | S_Shore | TSS200 |  | KRT85 | II | KRT85 (-108) | | | | | |
| cg09415754 | -0.1015 | 0.0117 | 0.1131 | 0.0008736 | Chr6:10385392 | Island |  |  |  | I | OFCC1 (-445811), TFAP2A (+30077) | | | | | |
| cg17133774 | -0.1161 | 0.0055 | 0.1216 | 0.0008779 | Chr1:6198667 | N_Shelf | Body |  | CHD5 | II | CHD5 (+41526), KCNAB2 (+92687) | | | | | |
| cg13756251 | 0.0388 | -0.0308 | -0.0697 | 0.0008869 | Chr9:137675723 |  | Body |  | COL5A1 | I | FCN2 (-96934), COL5A1 (+142072) | | | | | |
| cg04777348 | -0.1129 | -0.0043 | 0.1086 | 0.0008883 | Chr1:92952897 | S_Shore | TSS1500 |  | GFI1 | II | GFI1 (-1270) | | | | | |
| cg18788664 | 0.2137 | -0.0314 | -0.2451 | 0.0008917 | Chr1:221068171 | Island |  |  |  | I | HLX (+15429), DUSP10 (+847344) | | | | | |
| cg21052873 | -0.1010 | 0.0140 | 0.1150 | 0.0008924 | Chr12:124938573 | N_Shelf | Body |  | NCOR2 | II | NCOR2 (+113436), ZNF664 (+480812) | | | | | |
| cg07751331 | 0.1033 | -0.0049 | -0.1082 | 0.000895 | Chr2:26205865 | S_Shore | TSS1500 |  | KIF3C | I | KIF3C (-423) | | | | | |
| cg05878101 | 0.0401 | -0.0442 | -0.0843 | 0.0008959 | Chr7:150936167 | S_Shore | 3'UTR;3'UTR;3'UTR |  | SMARCD3 | II | CHPF2 (+6583), SMARCD3 (+9581) | | | | | |
| cg22638766 | 0.0997 | -0.0205 | -0.1202 | 0.0009007 | Chr7:99691486 |  | TSS1500;Body;TSS200 |  | MIR25;MCM7;MIR93 | II | AP4M1 (-7643), COPS6 (+4904) | | | | | |
| cg24459563 | 0.1222 | -0.0274 | -0.1496 | 0.000901 | Chr17:65040609 | N_Shore | TSS200 |  | CACNG1 | II | CACNG1 (-42) | | | | | |
| cg11212451 | 0.1747 | 0.0394 | -0.1354 | 0.0009041 | Chr5:173198508 |  |  |  |  | I | BOD1 (-154843), CPEB4 (-116822) | | | | | |
| cg19277154 | 0.0988 | -0.0660 | -0.1649 | 0.0009043 | Chr22:32438993 | N_Shore | TSS200 |  | SLC5A1 | II | SLC5A1 (-25) | | | | | |
| cg12563839 | -0.0033 | 0.0087 | 0.0120 | 0.0009044 | Chr14:91580507 |  | TSS1500;1stExon;5'UTR |  | C14orf159 | II | C14orf159 (-471) | | | | | |
| cg25456020 | -0.0993 | 0.0068 | 0.1061 | 0.0009051 | Chr17:159130 | S_Shore | Body |  | RPH3AL | II | DOC2B (-127711), RPH3AL (+43502) | | | | | |
| cg16352830 | 0.1648 | 0.0160 | -0.1488 | 0.0009062 | Chr1:208221036 |  | Body |  | PLXNA2 | II | CD34 (-136354), PLXNA2 (+196628) | | | | | |
| cg08845486 | 0.0181 | 0.0002 | -0.0179 | 0.0009146 | Chr16:67217724 | Island | 1stExon;5'UTR |  | KIAA0895L | I | EXOC3L1 (+6382), NOL3 (+9960) | | | | | |
| cg08642285 | -0.0447 | 0.0263 | 0.0710 | 0.0009164 | Chr15:68498589 |  | TSS200 |  | CALML4 | II | CALML4 (-142) | | | | | |
| cg20206437 | -0.0150 | 0.0052 | 0.0202 | 0.0009196 | Chr6:167276359 | Island | TSS1500 |  | RPS6KA2 | I | RPS6KA2 (-589) | | | | | |
| cg17883742 | 0.0248 | -0.0312 | -0.0560 | 0.0009216 | Chr19:57702371 | N_Shore | TSS1500 |  | ZNF264 | II | ZNF264 (-496) | | | | | |
| cg02957057 | 0.0017 | -0.0079 | -0.0096 | 0.0009272 | Chr1:236228598 | Island | TSS200 |  | NID1 | I | NID1 (-118) | | | | | |
| cg11504805 | -0.0211 | 0.0307 | 0.0518 | 0.0009308 | Chr13:114815119 | S_Shore | Body |  | RASA3 | II | GAS6 (-248074), RASA3 (+82975) | | | | | |
| cg27195771 | -0.0833 | 0.0782 | 0.1615 | 0.0009381 | Chr12:108605281 |  | Body |  | WSCD2 | II | WSCD2 (+81771), CMKLR1 (+109157) | | | | | |
| cg23531340 | -0.0774 | -0.0092 | 0.0682 | 0.0009389 | Chr2:18058900 | N_Shore | TSS1500 |  | KCNS3 | II | KCNS3 (-1044) | | | | | |
| cg12412079 | -0.0196 | 0.0081 | 0.0277 | 0.0009522 | Chr3:68981877 | Island | TSS200 |  | FAM19A4 | I | FAM19A4 (-117) | | | | | |
| cg07735220 | -0.1129 | 0.0243 | 0.1372 | 0.0009535 | Chr2:65280591 | N_Shelf |  |  |  | II | CEP68 (-2903) | | | | | |
| cg04554033 | -0.0198 | 0.0128 | 0.0326 | 0.0009546 | Chr17:77020041 | Island | TSS1500 |  | C1QTNF1 | I | C1QTNF1 (-209) | | | | | |
| cg03555325 | 0.1772 | 0.0223 | -0.1548 | 0.0009582 | Chr8:22458673 | S_Shore | Body |  | C8orf58 | II | KIAA1967 (-3865) | | | | | |
| cg18069403 | 0.0329 | -0.0407 | -0.0736 | 0.0009598 | Chr3:112565651 |  | TSS1500 |  | CD200R1L | II | CD200R1L (-855) | | | | | |
| cg23228341 | 0.0569 | -0.0189 | -0.0758 | 0.0009624 | Chr6:32818477 | N_Shelf | Body |  | TAP1 | II | PSMB9 (-3460), TAP1 (+3270) | | | | | |
| cg08853304 | -0.0636 | 0.0687 | 0.1323 | 0.0009626 | Chr5:172030548 |  |  |  |  | II | SH3PXD2B (-149022), NEURL1B (-37727) | | | | | |
| cg03986574 | 0.0464 | -0.0197 | -0.0662 | 0.0009653 | Chr11:64544080 | N_Shore | Body |  | SF1 | II | PYGM (-15894), SF1 (+1857) | | | | | |
| cg22917346 | -0.0207 | 0.0256 | 0.0463 | 0.0009716 | Chr13:44453235 | Island | 5'UTR;TSS200;TSS1500 |  | CCDC122;C13orf31 | II | SERP2 (-494742), ENOX1 (-249623) | | | | | |
| cg22651493 | -0.0389 | 0.0396 | 0.0785 | 0.0009719 | Chr17:35242447 |  |  |  |  | II | LHX1 (-52324), MRM1 (+284423) | | | | | |
| cg04840702 | -0.0289 | 0.0457 | 0.0746 | 0.0009723 | Chr4:174422626 | S_Shore |  |  |  | II | SCRG1 (-102010), HAND2 (+28751) | | | | | |
| cg07143125 | -0.1468 | -0.0014 | 0.1454 | 0.0009729 | Chr11:48110045 |  | Body |  | PTPRJ | II | OR4B1 (-128316), PTPRJ (+107936) | | | | | |
| cg01946784 | 0.0324 | -0.0224 | -0.0548 | 0.000983 | Chr2:121991815 |  | Body |  | TFCP2L1 | II | TFCP2L1 (+50962), GLI2 (+436949) | | | | | |
| cg05874355 | 0.1614 | 0.0484 | -0.1130 | 0.0009845 | Chr11:71724677 |  | Body |  | NUMA1 | II | LRTOMT (-66699), IL18BP (+14720) | | | | | |
| cg20925233 | -0.1703 | -0.0162 | 0.1541 | 0.0009852 | Chr17:47661136 |  | 3'UTR |  | NXPH3 | II | NXPH3 (+7839), SPOP (+94388) | | | | | |
| cg12192566 | 0.1253 | -0.0503 | -0.1757 | 0.0009867 | Chr5:140011377 | N_Shore | 3'UTR |  | CD14 | II | CD14 (+1657), SLC35A4 (+66958) | | | | | |
| cg16872841 | -0.1601 | -0.0104 | 0.1497 | 0.000988 | Chr13:110993558 |  | Body |  | COL4A2 | I | COL4A2 (+33928), RAB20 (+220512) | | | | | |
| cg08451978 | 0.0914 | -0.0243 | -0.1156 | 0.0009975 | Chr3:111821058 |  | Body |  | C3orf52 | II | C3orf52 (+15877), GCET2 (+31093) | | | | | |
| cg09171931 | 0.1751 | 0.0593 | -0.1158 | 0.0009996 | Chr11:34876740 |  |  |  |  | II | PDHX (-60936), EHF (+222730) | | | | | |
| Ranked by P value. DMPs, differentially methylated positions; MZ, monozygotic; GREAT, Genomic Regions Enrichment of Annotations Tool; TSS, transcription start site. | | | | | | | | | | | | | | |  |  |

| **Supplementary Table S6. Gene Ontology enrichment analysis of longitudinal DMPs** **between 5-10 years of age** | | | | |  |  | |
| --- | --- | --- | --- | --- | --- | --- | --- |
| **GO Accession ID** | | **GO Function** | **Ontology** | **P value** | | |  |
| GO:0032926 | negative regulation of activin receptor signaling pathway | | BP | 0.00060 | | |  |
| GO:0060429 | epithelium development | | BP | 0.00061 | | |  |
| GO:0043954 | cellular component maintenance | | BP | 0.00120 | | |  |
| GO:0009411 | response to UV | | BP | 0.00158 | | |  |
| GO:0071364 | cellular response to epidermal growth factor stimulus | | BP | 0.00398 | | |  |
| GO:0032925 | regulation of activin receptor signaling pathway | | BP | 0.00495 | | |  |
| GO:0030126 | COPI vesicle coat | | CC | 0.00547 | | |  |
| GO:0070849 | response to epidermal growth factor | | BP | 0.00590 | | |  |
| GO:0060850 | regulation of transcription involved in cell fate commitment | | BP | 0.00653 | | |  |
| GO:0045666 | positive regulation of neuron differentiation | | BP | 0.00678 | | |  |
| GO:0030663 | COPI-coated vesicle membrane | | CC | 0.00693 | | |  |
| GO:0030120 | vesicle coat | | CC | 0.00755 | | |  |
| GO:0045686 | negative regulation of glial cell differentiation | | BP | 0.00789 | | |  |
| GO:0035272 | exocrine system development | | BP | 0.00903 | | |  |
| GO:0021517 | ventral spinal cord development | | BP | 0.00969 | | |  |
| GO:0006071 | glycerol metabolic process | | BP | 0.00993 | | |  |
| GO:0010008 | endosome membrane | | CC | 0.00999 | | |  |
| GO:0032835 | glomerulus development | | BP | 0.01062 | | |  |
| GO:0017069 | snRNA binding | | MF | 0.01142 | | |  |
| GO:0044440 | endosomal part | | CC | 0.01143 | | |  |
| GO:0030137 | COPI-coated vesicle | | CC | 0.01198 | | |  |
| GO:0019400 | alditol metabolic process | | BP | 0.01256 | | |  |
| GO:0030238 | male sex determination | | BP | 0.01286 | | |  |
| GO:1901385 | regulation of voltage-gated calcium channel activity | | BP | 0.01335 | | |  |
| GO:0030855 | epithelial cell differentiation | | BP | 0.01380 | | |  |
| GO:0042219 | cellular modified amino acid catabolic process | | BP | 0.01381 | | |  |
| GO:0002070 | epithelial cell maturation | | BP | 0.01405 | | |  |
| GO:0045665 | negative regulation of neuron differentiation | | BP | 0.01636 | | |  |
| GO:0003211 | cardiac ventricle formation | | BP | 0.01666 | | |  |
| GO:0001738 | morphogenesis of a polarized epithelium | | BP | 0.01685 | | |  |
| GO:0072006 | nephron development | | BP | 0.01789 | | |  |
| GO:0048665 | neuron fate specification | | BP | 0.01793 | | |  |
| GO:0032924 | activin receptor signaling pathway | | BP | 0.01827 | | |  |
| GO:0014014 | negative regulation of gliogenesis | | BP | 0.01932 | | |  |
| GO:0051896 | regulation of protein kinase B signaling | | BP | 0.01973 | | |  |
| GO:0010977 | negative regulation of neuron projection development | | BP | 0.01985 | | |  |
| GO:0008026 | ATP-dependent helicase activity | | MF | 0.02026 | | |  |
| GO:0070035 | purine NTP-dependent helicase activity | | MF | 0.02026 | | |  |
| GO:0051897 | positive regulation of protein kinase B signaling | | BP | 0.02032 | | |  |
| GO:0007431 | salivary gland development | | BP | 0.02120 | | |  |
| GO:0009888 | tissue development | | BP | 0.02164 | | |  |
| GO:0005161 | platelet-derived growth factor receptor binding | | MF | 0.02273 | | |  |
| GO:0003207 | cardiac chamber formation | | BP | 0.02313 | | |  |
| GO:0048712 | negative regulation of astrocyte differentiation | | BP | 0.02357 | | |  |
| GO:0021522 | spinal cord motor neuron differentiation | | BP | 0.02358 | | |  |
| GO:0050714 | positive regulation of protein secretion | | BP | 0.02525 | | |  |
| GO:0004386 | helicase activity | | MF | 0.02566 | | |  |
| GO:0021535 | cell migration in hindbrain | | BP | 0.02779 | | |  |
| GO:0003148 | outflow tract septum morphogenesis | | BP | 0.02908 | | |  |
| GO:0010657 | muscle cell apoptotic process | | BP | 0.02957 | | |  |
| GO:0045664 | regulation of neuron differentiation | | BP | 0.02963 | | |  |
| GO:0043034 | costamere | | CC | 0.03021 | | |  |
| GO:0060037 | pharyngeal system development | | BP | 0.03037 | | |  |
| GO:0007160 | cell-matrix adhesion | | BP | 0.03043 | | |  |
| GO:0021795 | cerebral cortex cell migration | | BP | 0.03151 | | |  |
| GO:0048016 | inositol phosphate-mediated signaling | | BP | 0.03190 | | |  |
| GO:0002009 | morphogenesis of an epithelium | | BP | 0.03203 | | |  |
| GO:0043588 | skin development | | BP | 0.03346 | | |  |
| GO:0043491 | protein kinase B signaling | | BP | 0.03367 | | |  |
| GO:0000083 | regulation of transcription involved in G1/S transition of mitotic cell cycle | | BP | 0.03460 | | |  |
| GO:0001822 | kidney development | | BP | 0.03466 | | |  |
| GO:0045177 | apical part of cell | | CC | 0.03608 | | |  |
| GO:0050715 | positive regulation of cytokine secretion | | BP | 0.03723 | | |  |
| GO:0004714 | transmembrane receptor protein tyrosine kinase activity | | MF | 0.03787 | | |  |
| GO:0021520 | spinal cord motor neuron cell fate specification | | BP | 0.03968 | | |  |
| GO:0001708 | cell fate specification | | BP | 0.03986 | | |  |
| GO:0003714 | transcription corepressor activity | | MF | 0.04013 | | |  |
| GO:0019864 | IgG binding | | MF | 0.04024 | | |  |
| GO:0032760 | positive regulation of tumor necrosis factor production | | BP | 0.04034 | | |  |
| GO:0021895 | cerebral cortex neuron differentiation | | BP | 0.04082 | | |  |
| GO:0030334 | regulation of cell migration | | BP | 0.04086 | | |  |
| GO:0051239 | regulation of multicellular organismal process | | BP | 0.04130 | | |  |
| GO:0008285 | negative regulation of cell proliferation | | BP | 0.04157 | | |  |
| GO:0070851 | growth factor receptor binding | | MF | 0.04194 | | |  |
| GO:0051291 | protein heterooligomerization | | BP | 0.04262 | | |  |
| GO:0003215 | cardiac right ventricle morphogenesis | | BP | 0.04281 | | |  |
| GO:0043666 | regulation of phosphoprotein phosphatase activity | | BP | 0.04495 | | |  |
| GO:0008283 | cell proliferation | | BP | 0.04540 | | |  |
| GO:0010001 | glial cell differentiation | | BP | 0.04569 | | |  |
| GO:0007530 | sex determination | | BP | 0.04655 | | |  |
| GO:0021801 | cerebral cortex radial glia guided migration | | BP | 0.04705 | | |  |
| GO:0001976 | neurological system process involved in regulation of systemic arterial blood pressure | | BP | 0.04725 | | |  |
| GO:0019751 | polyol metabolic process | | BP | 0.04738 | | |  |
| GO:0048708 | astrocyte differentiation | | BP | 0.04775 | | |  |
| GO:0016266 | O-glycan processing | | BP | 0.04812 | | |  |
| GO:0001772 | immunological synapse | | CC | 0.04860 | | |  |
| GO:0048662 | negative regulation of smooth muscle cell proliferation | | BP | 0.04883 | | |  |
| GO:0005605 | basal lamina | | CC | 0.04883 | | |  |
| GO:0045685 | regulation of glial cell differentiation | | BP | 0.04930 | | |  |
| GO:0022029 | telencephalon cell migration | | BP | 0.04955 | | |  |
| Ranked by P value. GO: Gene Ontology, DMPs: Differentially Methylated Positions; BP: Biological process | | | | | | |  |
| MF: Molecular Function; CC: Cellular | | |  |  | | |  |

| **Supplementary Table S7.** Monozygotic twin Group Details | |  | | |  |
| --- | --- | --- | --- | --- | --- |
| **MZ twin Group** | | | **Number of Twin pairs** | **Males n (%)** | |
| Asthma Discordant at age 10 | | | 37 | 18/37 (48.6) | |
| Persistent Asthma Discordant sub-group (10-18 years) | | | 13 | 5/13 (38.5) | |
| Remission Asthma sub-group (discordant at age 10 but both unaffected at age 18) | | | 20 | 23/40 (57.5) | |
| Concordant Asthma unaffected (both twins unaffected at ages 10 and 18) | | | 19 | 12/19 (63.2) | |
| MZ: Monozygotic |  | | | |  |
